# VISFATIN: a novel modulator of placental endocrinology under physiological and pathological conditions—in vitro studies involving INSR, ERK1/2, and PKA signalling pathways

**DOI:** 10.1038/s41598-026-52422-w

**Published:** 2026-05-24

**Authors:** Monika Dawid, Wiktoria Gieras, Tomasz Milewicz, Agnieszka Rak

**Affiliations:** 1 Laboratory of Physiology and Toxicology of Reproduction, Faculty of Biology, Institute of Zoology and Biomedical Research, Jagiellonian University, Gronostajowa 9, 30-387 Kraków, Poland; 2 Doctoral School of Exact and Natural Sciences, Jagiellonian University, Profesora Stanisława Łojasiewicza 11, 30-348 Kraków, Poland; 3 Department of Gynaecological Endocrinology, Faculty of Medicine, Jagiellonian University Medical College, Mkołaja Kopernika 23, 31-501 Kraków, Poland

**Keywords:** Trophoblasts, Placental explants, Adipokines, Hormone secretion, Pregnancy pathologies, Cell biology, Molecular biology, Diseases, Endocrinology

## Abstract

This study aimed to determine the in vitro effects of visfatin on selected endocrine mediators in the human placenta. Using placental BeWo cells and villous explants from normal pregnancies and those complicated by intrauterine growth restriction (IUGR), preeclampsia (PE), and gestational diabetes mellitus (GDM), we determined the effects of visfatin on 3β-hydroxysteroid dehydrogenase (3β-HSD), aromatase (CYP19), human chorionic gonadotropin (hCG), human placental lactogen (hPL), placental growth hormone (GH2) expression, and the secretion of progesterone (P_4_), estradiol (E_2_) and mentioned protein hormones. We also investigated the effects of visfatin on the phosphorylation of protein kinase A (PKA), and hormone secretion after the pharmacological inhibition of insulin receptor (INSR), extracellular signal-regulated kinase 1/2 (ERK1/2), and PKA. We noted that visfatin increased or decreased the mRNA and protein expression of *HSD3B1*/3β-HSD, *CYP19A1*/CYP19, *CGB3*/hCG, *CSH1*/hPL, and *GH2*/GH2 in the tested groups. Additionally, visfatin lowered the secretion of P_4_, E_2_, hCG, hPL, and GH2 in BeWo cells and normal placenta, but modulated it in placenta explants from the pathological groups. The secretion of E_2_, hPL and GH2 was mediated by INSR, ERK1/2, and PKA, while P_4_ by INSR and ERK1/2. These data indicated that visfatin acting via selected molecular pathways may be an important regulator of placental endocrine function during normal and complicated pregnancies.

**Supplementary Information:**

The online version contains supplementary material available at 10.1038/s41598-026-52422-w.

## Introduction

The placenta is an enigmatic organ that organises the complex molecular processes crucial for foetal development and pregnancy outcomes^1^. Endocrinology is one of the most important processes, as it relies on synthesis and secretion of numerous hormones. During steroidogenesis, which is controlled by enzymes such as 3β-hydroxysteroid dehydrogenase (3β-HSD) and aromatase (CYP19), the placenta specialises in producing steroids such as progesterone (P_4_) and estradiol (E_2_) and protein hormones like human chorionic gonadotropin (hCG), human placental lactogen (hPL), and placental growth hormone (GH2), which together provide this transitional foetal organ a great deal of control over the proper course of pregnancy^2^. Placental endocrinology is regulated primarily by the signalling pathways of multiple protein kinases, including protein kinase A (PKA) and extracellular signal-activated kinase (ERK1/2)^3–5,^ and many factors, including adipokines—hormones produced by white adipose tissue; for example, leptin reduces the secretion of P_4_, E_2_, and GH2, while increasing hCG in human placenta cells^6–8^. Adiponectin reduces hCG, hPL, and P_4_ levels in human trophoblastic cells^9^.

Visfatin was identified as a pre-B cell colony-enhancing factor in 1994, but its current designation, reflecting its highest expression in visceral adipose tissue, has been used since 2005^10^. Visfatin is a pleiotropic hormone that modulates processes related to the development of obesity and affects reactions related to oxidative stress, lipid or glucose metabolism, insulin resistance, and inflammation^11^. In human monocytes, visfatin has a proinflammatory effect, inducing the production of interleukins (IL-1β, IL-6), suggesting potential impact on inflammatory disorders^12^. There has been an ongoing debate regarding its potential action via the insulin receptor (INSR). Visfatin upregulates insulin levels and increases the phosphorylation of INSR and ERK1/2 in mouse pancreatic β cells^13^. Silencing the receptor blocks visfatin-induced AMP-activated protein kinase phosphorylation and glucose uptake in mouse skeletal muscle cells^14^. The levels of visfatin present in the maternal body increase during pregnancy; in the first trimester, they are lower than in the second or third trimesters and drop following delivery^15,16^. Moreover, the level of visfatin in women with pregnancy pathologies such as intrauterine growth restriction (IUGR), preeclampsia (PE), or gestational diabetes mellitus (GDM) has yet to be determined^17^. These pathologies are among the most prevalent disorders and affect around 10% of pregnancies. IUGR manifests as a smaller foetal size compared to gestational age. PE usually occurs after the 20^th^ week of pregnancy and is associated with hypertension and damage to the kidneys and liver. GDM is a metabolic disorder in which an increase in the level of glucose in the mother’s blood is observed despite the patient being previously not diagnosed with diabetes^18^. Although most studies have indicated an increase in the level of visfatin in maternal circulation in each of the above pathologies^19,20^, there are also studies that have indicated a decrease in its level^21,22^ or no differences compared to normal pregnancies, as in PE and GDM^23,24^. Our previous studies showed that visfatin has anti-proliferative and pro-apoptotic roles in human trophoblasts and placentas from IUGR, PE, and GDM pregnancies^25^. However, data on the primary function of the placenta was still lacking. This includes its role in producing hormones that play a key role in aspects such as embryo implantation, regulating the mother’s immunological tolerance, and transporting nutrients. Disturbances in the release of these hormones may be associated with the development of pregnancy pathologies^2^.

In light of these findings, we hypothesised that visfatin regulates human placental endocrinology by affecting the expression and release of key factors via specific signalling pathways. We aimed to determine the in vitro impact of visfatin on the mRNA and protein expression of 3β-HSD, CYP19, hCG, hPL, and GH2, as well as the secretion of P_4_, E_2_, hCG, hPL, and GH2 in BeWo cells and term villi from normal and complicated by IUGR, PE, and GDM pregnancies. Moreover, we aimed to examine the effect of visfatin on PKA phosphorylation and its involvement via INSR, ERK1/2, and PKA in placental hormone secretion. 

## Results

### The modulating role of visfatin on the expression of 3β-HSD, CYP19, and its inhibiting effect on P_4_ and E_***2***_ levels in BeWo cells

**Fig. 1:**
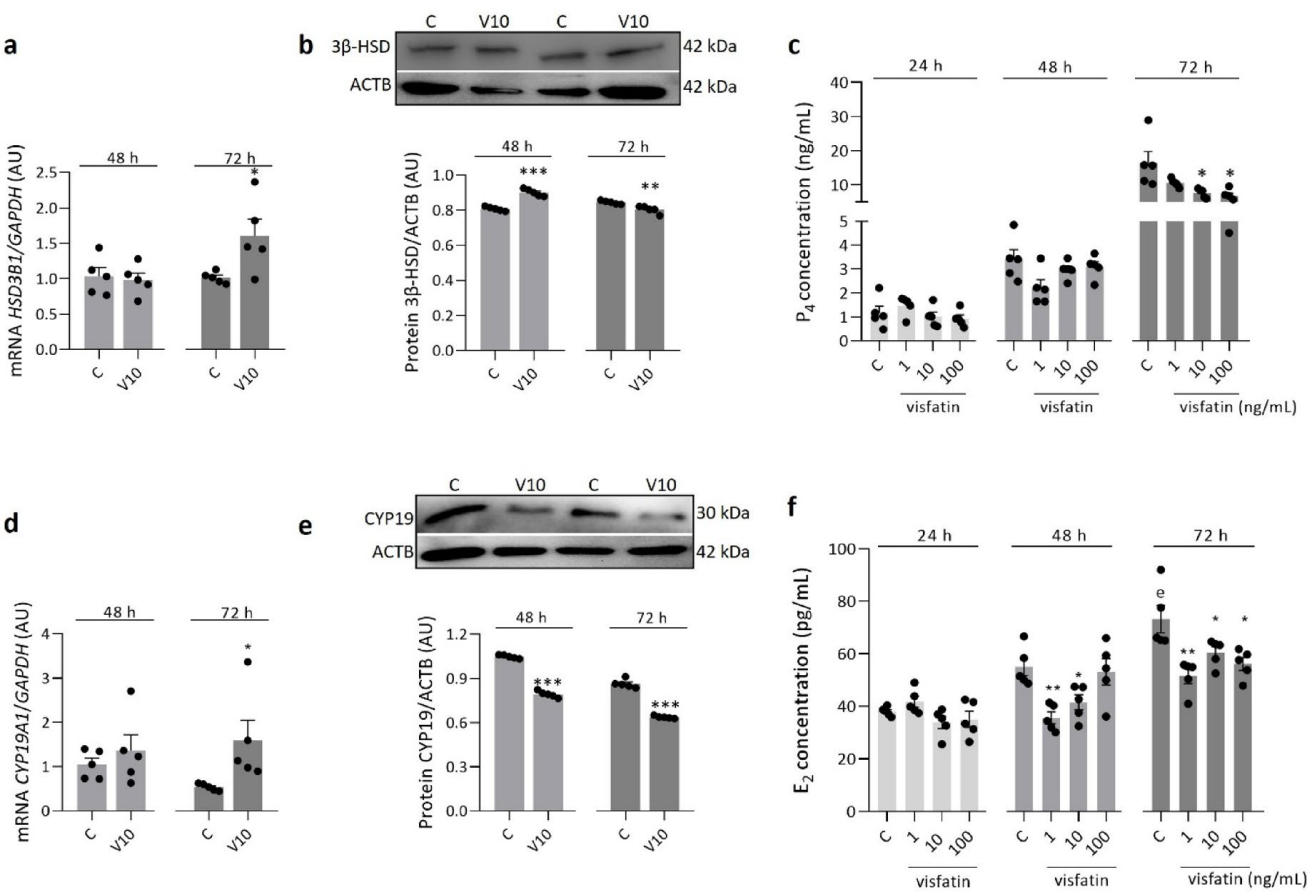
In vitro effect of visfatin on the *HSD3B1*/ 3β-HSD/ P_4_ (**a**–**c**) and *CYP19A1*/ CYP19/ E_2_ (**d**–**f**) levels in BeWo cells. Statistical analysis was performed using a two-way ANOVA followed by a followed by Sidak’s multiple comparisons test (mean ± SEM, *p* < 0.05). The mRNA and protein expressions of the studied factors were examined after 48 and 72 h of incubation, while the steroid concentrations were examined after 24, 48, and 72 h in BeWo cells. 3β-HSD, 3β-hydroxysteroid dehydrogenase; P_4_, progesterone; CYP19, aromatase; E_2_, estradiol; C, control; V, Visfatin (1, 10, 100 ng/mL); ACTB, β-actin; AU- arbitrary units.

Specifically, 10 ng/mL visfatin did not affect the *HSD3B1* mRNA expression after 48 h, but increased it after 72 h of incubation in comparison with the control group (*p* = 0.0344) (Fig. 1a). However, it increased the 3β-HSD protein expression after 48 h (*p* = 0.0001), while decreasing it after 72 h (*p* = 0.004) (Fig. 1b). Furthermore, visfatin reduced P_4_ levels at 10 ng/mL (*p* = 0.033), and 100 ng/mL (*p* = 0.024) after 72 h *vs* control (Fig. 1c).

An increase in *CYP19A1* mRNA expression after 72 h (*p* = 0.049) (Fig. 1d) and a reduction in the protein expression of CYP19 after 48 h (*p* = 0.0001) and 72 h (*p* = 0.0001) (Fig. 1e) were observed following incubation 10 ng/mL visfatin in comparison with the control. Following a 48 h of incubation, visfatin at 1 ng/mL (*p* = 0.001) and 10 ng/mL (*p* = 0.016), and all studied concentrations: 1 ng/mL (*p* = 0.006), 10 ng/mL (*p* = 0.048) and 100 ng/mL (*p* = 0.019) after 72 h significantly reduced E_2_ secretion (Fig. 1f).

### The modulating role of visfatin on the expression and its inhibiting effect on hCG, hPL, GH2 levels in BeWo cells

**Fig. 2:**
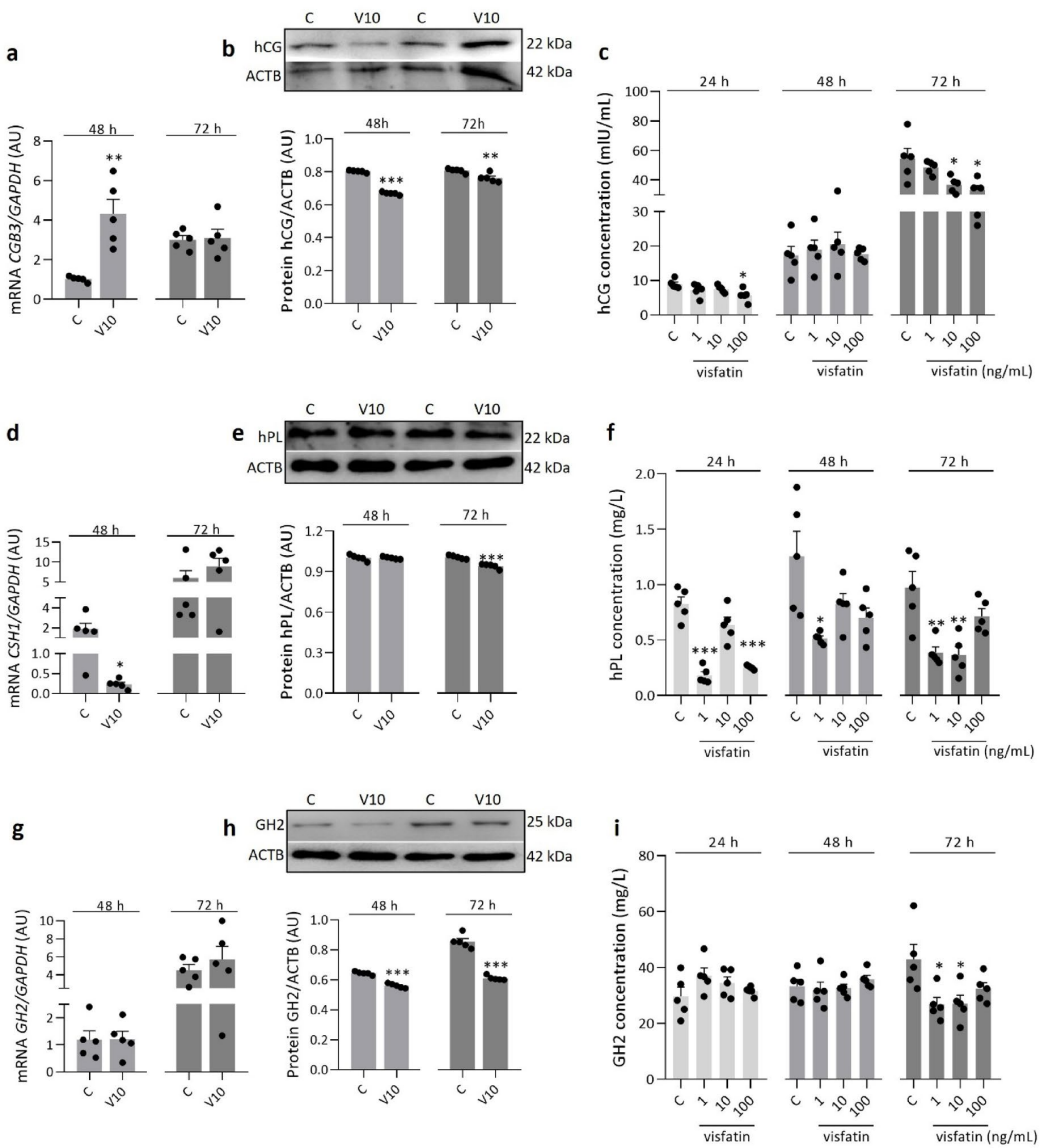
The in vitro effect of visfatin on the *CGB3*/ hCG (**a**–**c**), *CSH1*/ hPL (**d**–**f**), and *GH2*/ GH2 (**g**–**i**) levels in BeWo cells. Statistical analysis was performed using a two-way ANOVA followed by a followed by Sidak’s multiple comparisons test (mean ± SEM, *p* < 0.05). The mRNA and protein expressions of the studied factors were examined after 48 and 72 h of incubation, while protein hormone concentrations were examined after 24, 48, and 72 h in BeWo cells. hCG, human chorionic gonadotropin; hPL, human placental lactogen; GH2, human placental growth hormone; C, control; V, visfatin (1, 10, 100 ng/mL); ACTB, β-actin; AU- arbitrary units.

After 48 h of incubation, 10 ng/mL visfatin increased (*p* = 0.002) *CGB3* mRNA expression (Fig. 2a), and it decreased the protein expression of the hCG after 48 h (*p* < 0.001) and 72 h (*p* = 0.009) (Fig. 2b). Visfatin at 100 ng/mL (*p* = 0.011) reduced hCG secretion after 24 h, and a similar effect was observed at 10 ng/mL (*p* = 0.039) and 100 ng/mL (*p* = 0.021) after 72 h *vs* controls (Fig. 2c).

Visfatin at 10 ng/mL (*p* = 0.039) decreased *CSH1* mRNA expression after 48 h (Fig. 2d) and hPL (*p* = 0.0002) protein expression after 72 h (Fig. 2e). Furthermore, visfatin at 1 ng/mL (*p* = 0.0001) and 100 ng/mL (*p* = 0.0001) decreased hPL secretion after 24 h, whereas a similar effect was observed at a concentration of 1 ng/mL (*p* = 0.012) after 48 h and at 1 ng/mL (*p* = 0.005) as well as at 10 ng/mL (*p* = 0.006) after 72 h (Fig. 2f).

Finally, visfatin did not change GH2 mRNA expression after 48 and 72 h compared with time-matched controls (Fig. 2g); however, visfatin at 10 ng/mL reduced GH2 protein expression at 48 h (*p* = 0.001) and 72 h (*p* = 0.001) (Fig. 2h). Moreover, concentrations of 1 ng/mL (*p* = 0.023) and 10 ng/mL (*p* = 0.029) decreased GH2 secretion after 72 h (Fig. 2i).

### The modulating role of visfatin on the expression of 3β-HSD, CYP19, and its inhibiting effect on P_4_ and E_2_ levels in normal, IUGR, PE, and GDM placental explants

**Fig. 3:**
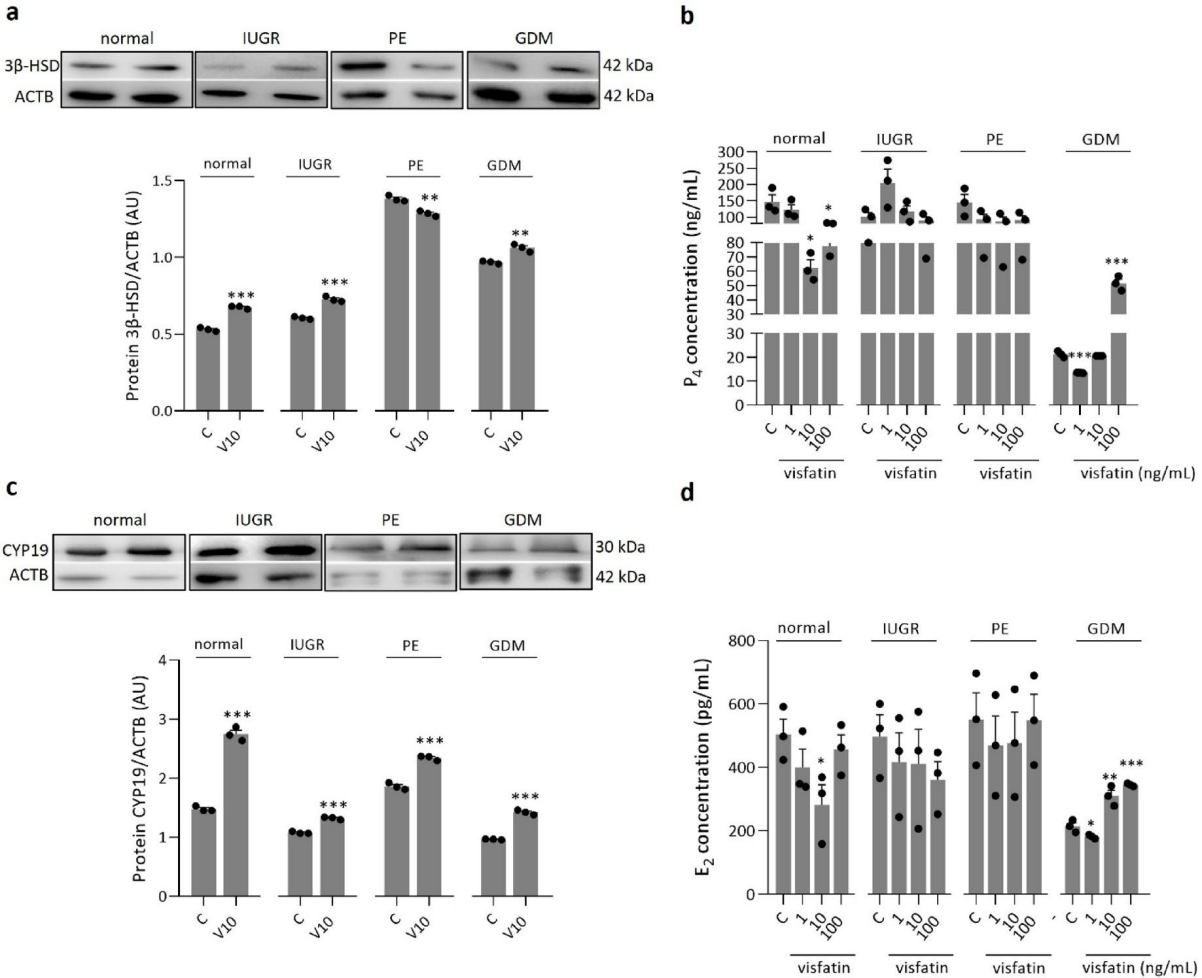
The in vitro effect of visfatin on the 3β-HSD/ P_4_ (**a**–**b**) and CYP19/ E_2_ (**c**–**d**) levels in normal, IUGR, PE, and GDM placentas. Statistical analysis was performed using a Student’s t-test (for protein expression) and a two-way ANOVA followed by a followed by Sidak’s multiple comparisons test (mean ± SEM, *p* < 0.05). The protein expressions and concentrations of the studied factors were examined after 72 h of incubation in each group. IUGR, intrauterine growth restriction; PE, preeclampsia; GDM, gestational diabetes mellitus; 3β-HSD, 3β-hydroxysteroid dehydrogenase; P_4_, progesterone; CYP19, aromatase; E_2_- estradiol; C, control; V- Visfatin (1, 10, 100 ng/mL); ACTB, β-actin; AU, arbitrary units.

Visfatin at 10 ng/mL enhanced the expression of 3β-HSD protein in normal (*p* = 0.0001), IUGR (*p* = 0.0004), and GDM (*p* = 0.004) placental explants; in the PE group, it reduced (*p* = 0.003) this expression after 72 h (Fig. 3a). Additionally, visfatin at 10 ng/mL (*p* = 0.019) and 100 ng/mL (*p* = 0.034) decreased the P_4_ levels in normal explants; at 1 ng/mL (*p* = 0.0006), P_4_ secretion decreased, whereas at 100 ng/mL (*p* = 0.0005) it increased in the GDM group compared with the control. No significant changes were noted in the IUGR and PE groups (Fig. 3b).

Furthermore, visfatin increased the CYP19 protein expression in both normal (*p* = 0.001), IUGR (*p* = 0.0002), PE (*p* = 0.0003), and GDM (*p* = 0.0001) groups in comparison to the control (Fig. 3c). E_2_ secretion decreased in the normal placental explants under the influence of visfatin at 10 ng/mL (*p* = 0.049), whereas in the GDM, visfatin at 1 ng/mL (*p* = 0.049) decreased, and 10 ng/mL (*p* = 0.011) and 100 ng/mL (*p* = 0.0003) increased hormone release. Nevertheless, no notable alterations in E_2_ secretion were observed in IUGR and PE villous explants following visfatin treatment (Fig. 3d).

### The modulating role of visfatin on the expression and its inhibiting effect on hCG, hPL, GH2 levels in normal, IUGR, PE, and GDM placental explants

**Fig. 4:**
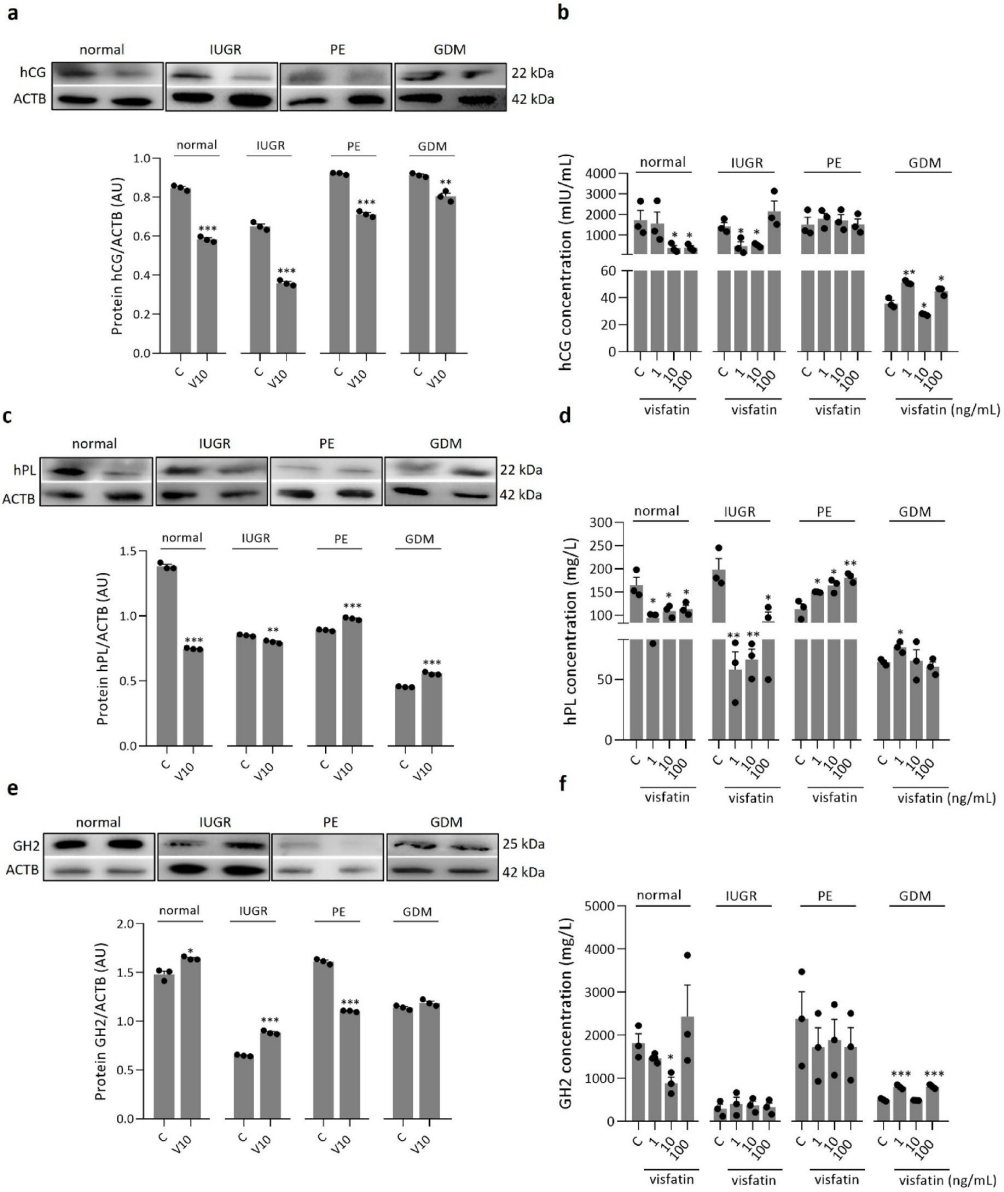
The in vitro effect of visfatin on the hCG (**a**–**b**), hPL (**c**–**d**), and GH2 (**e**–**f**) levels in normal, IUGR, PE, and GDM placentas. Statistical analysis was performed using a Student’s t-test (for protein expression) and a two-way ANOVA followed by a followed by Sidak’s multiple comparisons test (mean ± SEM, *p* < 0.05). The protein expressions and concentrations of the studied factors were examined after 72 h of incubation in each group. IUGR, intrauterine growth restriction; PE, preeclampsia; GDM, gestational diabetes mellitus; hCG, human chorionic gonadotropin; hPL, human placental lactogen; GH2, human placental growth hormone; C, control; V, visfatin (1, 10, 100 ng/mL); ACTB, β-actin; AU, arbitrary units.

After 72 h, visfatin at 10 ng/mL reduced hCG protein expression in normal (*p* = 0.0001), IUGR (*p* = 0.0001), PE (*p* = 0.0001), and GDM (*p* = 0.0029) placental explants (Fig. 4a). Moreover, decreased hCG secretion was noted in the normal explants under the influence of 10 ng/mL (*p* = 0.048) and 100 ng/mL (*p* = 0.047) visfatin, and in the IUGR after treatment with 1 ng/mL (*p* = 0.027) and 10 ng/mL (*p* = 0.0009) visfatin. In GDM, visfatin at 1 ng/mL (*p* = 0.002) and 100 ng/mL (*p* = 0.028) increased, hCG release while at 10 ng/mL (*p* = 0.017), it decreased it (Fig. 4b).

Moreover, visfatin at 10 ng/mL decreased hPL expression in normal (*p* = 0.0001) and IUGR (*p* = 0.003) placental explants but increased it in PE (*p* = 0.0003) and GDM (*p* = 0.0002) (Fig. 4c). Additionally, visfatin at the tested concentrations decreased hPL secretion in normal (*p* = 0.007, *p* = 0.039, *p* = 0.049) and IUGR (*p* = 0.018, *p* = 0.006, *p* = 0.023) explants. Conversely, visfatin in all concentrations in PE (*p* = 0.033, *p* = 0.024, *p* = 0.006) and 1 ng/mL (*p* = 0.014) in GDM increased the hPL secretion (Fig. 4d).

Visfatin at 10 ng/mL increased GH2 protein expression in normal (*p* = 0.010) and IUGR (*p* = 0.0001) placental explants but decreased it in PE (*p* = 0.0001), while it had no effect on the GDM group (Fig. 4e). Interestingly, visfatin at 10 ng/mL decreased GH2 secretion in normal explants (*p* = 0.022) but increased it at 1 ng/mL (*p* = 0.0009) and 100 ng/mL (*p* = 0.0008) in GDM (Fig. 4f).

### Effect of visfatin on the phosphorylation of PKA and the involvement of INSR, ERK1/2, and PKA in visfatin acting on placental hormone secretion in BeWo cells

**Fig. 5:**
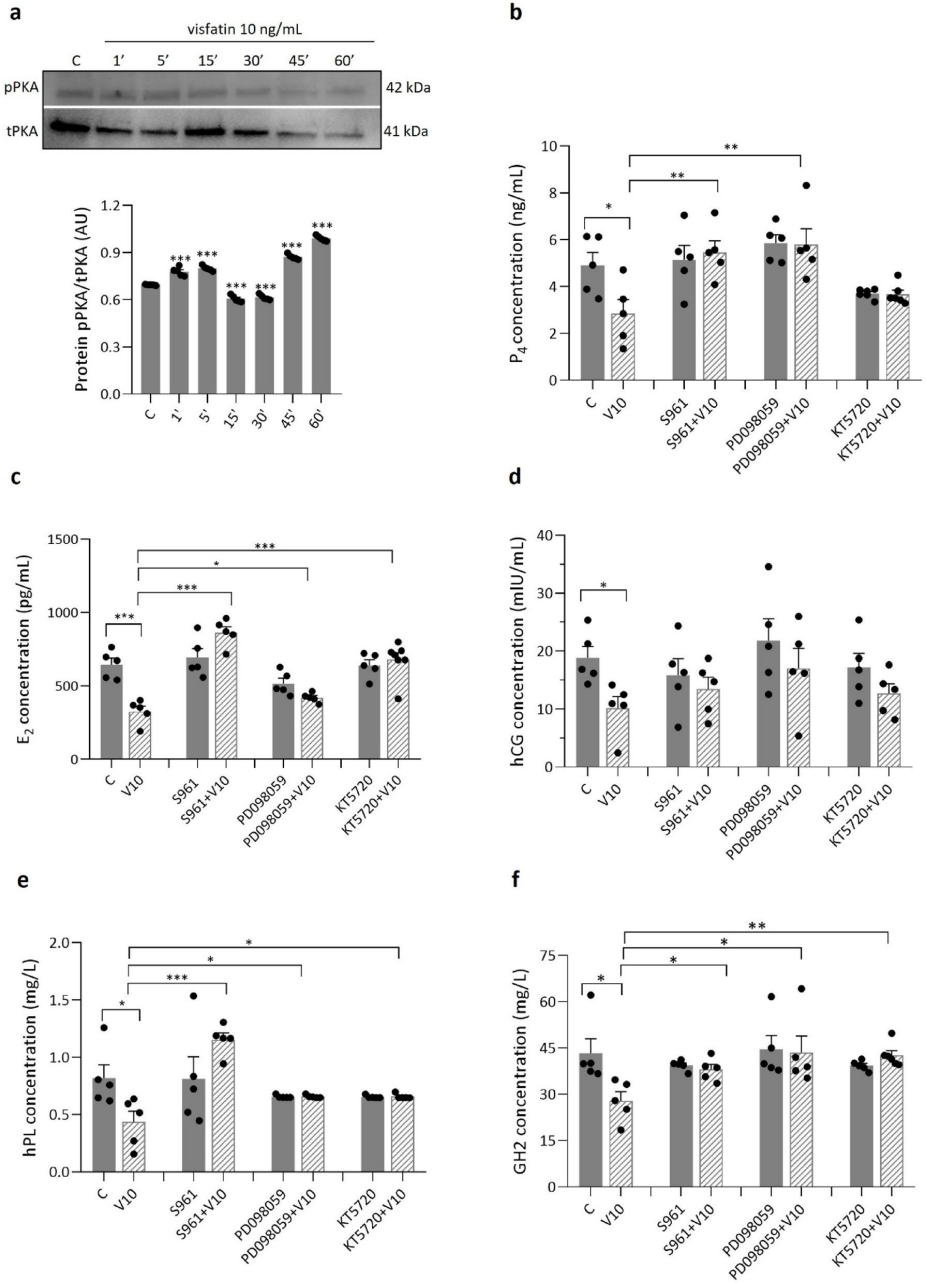
The effect of visfatin (10 ng/mL) on the PKA phosphorylation (**a**) and molecular mechanisms of P_4_ (**b**), E_2_ (**c**), hCG (**d**), hPL (**e**), and GH2 (**f**) secretion after the pharmacological inhibition of INSR, ERK1/2, and PKA in BeWo cells. Statistical analysis was performed using a two-way ANOVA followed by a followed by Sidak’s multiple comparisons test (mean ± SEM, *p* < 0.05). The protein expression of PKA was studied after 1, 5, 15, 30, 45, and 60 min, while hormone concentrations were examined after 72 h. C, control; V, visfatin (1, 10, 100 ng/mL); P_4_, progesterone; E_2_, estradiol; hCG, human chorionic gonadotropin; hPL, human placental lactogen; GH2, human placental growth hormone; INSR, insulin receptor; ERK1/2- extracellular signal-activated kinase, PKA, protein kinase A; S961, INSR antagonist, PD098059, ERK1/2 inhibitor, KT5720, PKA inhibitor, AU, arbitrary units.

Visfatin at 10 ng/mL modulated the phosphorylation of PKA in BeWo cells: it increased it after 1, 5, 45, and 60 min (*p* = 0.0001) and decreased it after 15 and 30 min (*p* = 0.0001) *vs* control (Fig. 5a).

The observed decrease in P_4_ (Fig. 5b) was reversed after incubation of the cells with visfatin at a concentration of 10 ng/mL in combination with S961 (*p* = 0.009) and PD098059 (*p* = 0.011) (Fig. 5b). Similarly, an increase in E_2_ was noted after co-treatment with visfatin together with S961 (*p* = 0.0001), PD98059 (*p* = 0.043) or KT5720 (*p* = 0.0003) (Fig. 5c) . No significant effect on hCG was observed after treatment with the inhibitors (Fig. 5d). The inhibitory effect of visfatin alone on hPL secretion was reversed by treating cells with visfatin in combination with the inhibitors S961 (*p* = 0.0002), PD098059 (*p* = 0.047), and KT5720 (*p* = 0.047) (Fig. 5e). In the case of GH2 release, a similar effect was observed due to cell co-stimulation with visfatin and both S961 (*p* = 0.016), PD098059 (*p* = 0.032), and KT5720 (*p* = 0.001) (Fig. 5f).

## Discussion

**Fig. 6:**
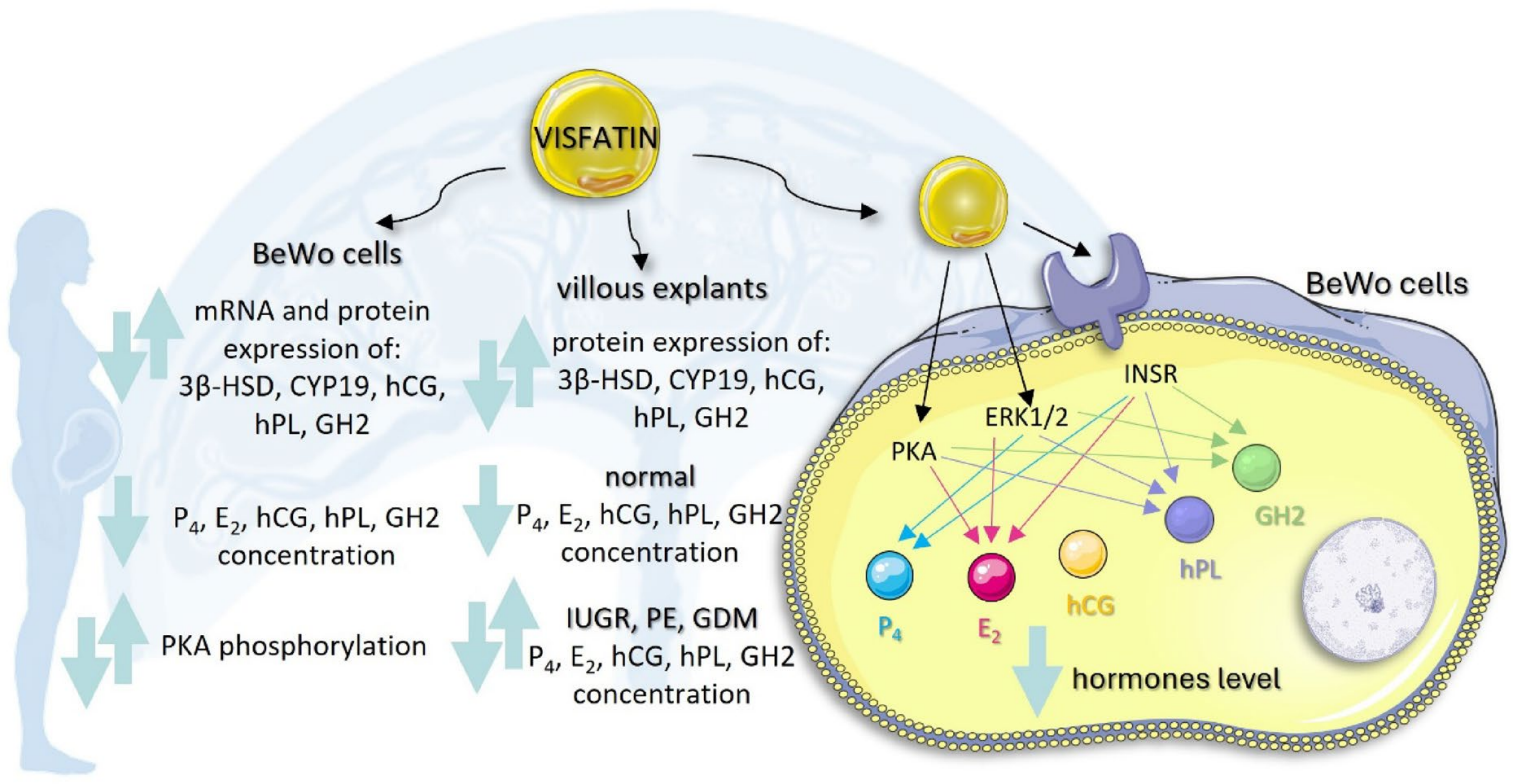
Summary of the results obtained. IUGR, intrauterine growth restriction; PE, preeclampsia; GDM, gestational diabetes mellitus; 3β-HSD, 3β-hydroxysteroid dehydrogenase; P_4_, progesterone; CYP19, aromatase; E_2_, estradiol; hCG, human chorionic gonadotropin; hPL, human placental lactogen; GH2, human placental growth hormone; INSR, insulin receptor; ERK1/2, extracellular signal-activated kinase; PKA, protein kinase A. The utilised graphics were licenced by BioRender (2025) https://BioRender.com/f09n409 and Servier Medical Art CC BY 4.0.

In this study, we demonstrated an in vitro effect of visfatin on human placental endocrinology (Fig. 6). In the BeWo cells, visfatin reduced the protein expression of 3β-HSD, CYP19, hCG, hPL, and GH2 as well as the secretion of P_4_, E_2_, hCG, hPL, and GH2 after 72 h. However, at the transcript levels of the selected factors, the results indicated an increase, decrease, or no effect following visfatin treatment. In the villi of placenta explants from normal pregnancies, similar to BeWo cells, visfatin reduced the secretion of hormones and modulated the protein expression of the studied factors after 72 h. In the placentas from IUGR, PE, and GDM pregnancies, visfatin also modulated protein expression; these results did not align with the data obtained for hormone secretion. Interestingly, visfatin increased hPL secretion in the PE and modulated it in the GDM in a dose-dependent manner. Moreover, our findings indicated that visfatin modulates the phosphorylation of PKA in BeWo cells, and different signalling pathways are involved in the visfatin inhibitory effect on hormone secretion; INSR and ERK1/2 are implicated in P_4_ secretion, INSR and PKA are involved in E_2_, and INSR, ERK1/2, and PKA are involved in hPL and GH2 release.

The literature data indicate that P_4_, E_2_, hCG, hPL, and GH2 exert a significant influence on the progression of pregnancy and foetal development. P_4_ has been demonstrated to stimulate the decidualisation of human endometrial stromal cells and embryo implantation, while inhibiting spontaneous uterine contractility^2^ and preparing the mammary glands for lactation^26^. E_2_ has been demonstrated to enhance embryo implantation and promote the differentiation and growth of the endometrium^27^. Furthermore, it regulates uteroplacental blood flow, increasing vasodilatation^28^, and elevates the proliferation of the mammary epithelium, preparing the tissue for lactation^26^. The hCG is essential for maintaining early pregnancy, promoting the production of P_4_ by the corpus luteum before being produced by the placenta^29^. Its action via the hCG receptor is vital for the differentiation of cytotrophoblasts into syncytiotrophoblasts^30^. It has been demonstrated that hCG also suppresses the maternal immunological system, resulting in an increase in regulatory T-cell numbers at the foetal-maternal interface^31^. Finally, hPL and GH2 demonstrate a substantial homogeneity^32^, and both are implicated in the enhancement of lipolysis in adipose tissue^33^. Considering this data with our results, it can be seen that visfatin plays a crucial regulatory role in many important functions throughout pregnancy by decreasing the levels of the aforementioned hormones. However, the visfatin’s effect on endocrine functions depends on the specific tissue and species in question. For example, visfatin has been demonstrated to significantly increased IGF-1-induced P_4_ and E_2_ secretion in human ovary (both primary human granulosa and KGN cells^34^. Moreover, it also enhanced the release of P_4_, E_2_ secretion, and 3β-HSD activity in bovine granulosa cells^35^. Furthermore, as demonstrated by Annie et al.^36^, the inhibition of visfatin by a specific inhibitor, FK866, resulted in increased estrogen secretion and the up-regulation of the expression of CYP19 in murine ovaries. Additionally, the research conducted by our team on the pig model has indicated that the effect of visfatin is contingent upon the phase of the estrous cycle: on days 2–3 and 14–16 of the estrous cycle , visfatin has been observed to reduce P_4_, while on days 10–12 of the estrous cycle, it has been shown to stimulate P_4_. Moreover, an increase in E_2_ secretion was observed on days 2–3, along with stimulation of steroidogenic marker expression on days 10–12 of the estrous cycle^37^. Consequently, it can be deduced that the divergent effects of visfatin on ovarian and placental steroidogenesis may be attributable to discrepancies in the mechanisms of these hormones’ production. Within the ovary, the production of steroid hormones is subject to rigorous regulation by gonadotropins and cyclical changes^38^. In contrast, placental steroidogenesis occurs continuously, independently of gonadotropins but dependent on maternal and foetal substrates^39^. However, returning to the data we obtained, it is also worth emphasizing that the observed discrepancies in the gene and protein expression of the examined endocrine factors, such as 3β-HSD, CYP19, hCG, hPL, and GH2, may be attributed to the fact that mRNA levels account for approximately 40% of the variability in protein levels, which are linked to transcription rates^40^.

In view of the results obtained from visfatin responses in the BeWo cell line, it was decided to verify whether the research model selected was adequate for observing changes actually occurring in the human placenta. The decision was made to expand the analyses to include placental explants from normal pregnancies as well as those complicated by IUGR, PE, and GDM. This observation is of particular significance as it suggests that the differential responses to visfatin observed among placental explants from various pregnancy complications may reflect distinct pathophysiological mechanisms underlying these conditions. For instance, normal pregnancy and IUGR are primarily associated with differences in placental growth and nutrient transport^41^, whereas PE and GDM are characterised by profound alterations in placental endocrine, inflammatory, and metabolic signalling^42^. In particular, PE and GDM have been linked to dysregulated adipokine signalling, oxidative stress, and altered insulin sensitivity^43,44^, which may modify placental responsiveness to visfatin. Returning to the data we obtained, the results of reduced hormone secretion in normal placentas are consistent with those obtained for BeWo, thereby reinforcing our conviction and corroborating previous reports indicating that this cell line can be employed to assess the physiological processes occurring in the organ^45,46^. Following treatment with visfatin, we observed dose-dependent increased secretions of hPL in PE and P_2_, E_2_, hCG, hPL, and GH2 in GDM placentas compared to normal pregnancies. The effect of the tested adipokine may depend on the dose used in the endocrine process, which was also proven earlier in our studies on the impact of visfatin on estradiol secretion and the expression of the 3β-HSD enzyme by the corpus luteum on the 10^th^–12^th^ days of the estrous cycle^37^. Moreover, previous studies have estimated appropriate hormone levels at variuous stages of pregnancy. In brief, in maternal blood, an increase was observed in E_2_ and P_4_, while a decrease was noted in hCG during the third trimester of normal pregnancy compared to previous stages^47^. The concentrations of hPL increased throughout pregnancy^48^, while the maternal plasma of placental GH levels increased after 24–25 weeks of gestation^49^. The levels in question vary depending on the pathological conditions present during pregnancy. Previous studies have demonstrated that women with PE exhibit lower mean plasma hPL levels compared with patients with uncomplicated pregnancies. In severe PE, these values were typically lower than in mild cases of PE^49^. The results of the present study suggest that visfatin may play a protective role in PE by regulating hPL levels during pregnancy. Maternal blood P_4_ levels are not associated with the risk of GDM^50^. Conversely, the cord blood E_2_ levels in the GDM group were significantly lower than in the control group, indicating its involvement in the pathophysiology of GDM^51^. Higher hCG levels in early pregnancy were associated with a lower risk of GDM^52^. Placental GH2 causes insulin resistance manifested by postprandial hyperinsulinemia and minimal glucose lowering in response to insulin injection. Thus, it may be a key factor in mediating the increase in insulin resistance during pregnancy^53^. Data from a systematic review and meta-analysis indicate that hPL levels in early pregnancy complicated by type 1 diabetes are lower than in the control group, and this is associated with delayed placental development. Conversely, in later pregnancy, the levels are higher due to the placental mass being larger^54^. Moreover, the observed selectivity of the response in placental villous explants derived from GDM pregnancies likely reflects the distinct metabolic and endocrine environment characteristic of GDM. This pathology is associated with altered glucose metabolism, insulin resistance, chronic low-grade inflammation, and dysregulated adipokine signalling^42^. These metabolic disturbances may lead to adaptive changes in placental endocrine function, resulting in altered baseline hormone production as well as increased sensitivity to visfatin stimulation^55^. Therefore, it is impossible to clearly state whether visfatin plays a protective or non-protective role in GDM. However, it undoubtedly has a regulatory effect on the hormones secreted by the placenta. Consequently, the modulation of its levels could be considered during individual trimesters of pregnancy to reduce the risk or eliminate the effects of this pregnancy pathology.

The diverse results obtained regarding the response of cells and villi to visfatin treatment suggested that the secretion of the placental hormones under study may be controlled by multiple signalling pathways. Therefore, based on the available literature, a decision was made to investigate the molecular pathways that most frequently control steroidogenesis and protein hormone secretion in the placenta^2,56^. In our studies, we observed that visfatin modulates the phosphorylation of PKA and that selected signalling pathways are involved in the molecular mechanisms of each placental hormone reduction in BeWo cells. In the case of P_4_, this process occurs through INSR and ERK1/2. The secretion of E_2_ decreases via INSR and PKA, while hPL and GH2 by INSR, ERK1/2, and PKA. In hCG, it does not occur in any of the tested paths. Our previous study demonstrated that visfatin elevates the expression of the INSR gene and protein and co-localizes with this receptor in the cytoplasmic compartment of BeWo cells. Moreover, it modulates the phosphorylation of ERK1/2 kinase, which, together with INSR, is implicated in the molecular mechanism of placental cell apoptosis in BeWo cells^25^. In turn, the PKA in human placental mitochondria may play a pivotal role in the cascade of steroidogenesis transduction, either directly through phosphorylating its substrates or indirectly through regulating other kinases and phosphatases^57^. One study demonstrated that the synthesis of P_4_ by human placental mitochondria is sensitive to PKA inhibition by H89^3^. The activation of the PKA pathway in human trophoblasts up-regulated the expression of 3β-HSD^58^. Therefore, why did the molecular mechanism of the release of P_4_ not occur via PKA in our studies? The data of Nemer et al.^59^ showed that in immortalised rat granulosa cells, protein kinase C (PKC) activators do not increase P_4_ secretion by themselves and, interestingly, they inhibit PKA-induced P_4_ secretion. In a study of corticotropin-releasing hormone, which reduces P_4_ secretion, like visfatin in the present study, the treatment of placental cells with a PKC inhibitor resulted in an increase in P_4_^60^. The lack of effect of visfatin on PKA that we observed may be a consequence of not blocking PKC in BeWo cells. Therefore, future studies should consider using a blocker of this kinase to re-examine the potential regulation of P_4_ by PKA-PKC signalling. In line with our studies on E_2_ secretion, PKA played a significant role in estrogen synthesis via the cooperative regulation of the organic anion transporter 4 and CYP19 in the human placental JEG-3 cells^4^. It has also been shown that placental hCG secretion is stimulated by PKA^61^ and MAPK (p38 and ERK 1/2)^5^; however, this does not apply to our studies regarding visfatin. Therefore, future studies should focus on other signalling pathways to elucidate this mechanism. One proposed kinase for future studies may consider protein kinase B, the inhibition of which, for example, reduced CG-induced metalloproteinase-2 expression, which is involved in placental cell invasion^62^. In line with our report, one study showed that MAPK signalling is engaged in the intracellular mechanism of apolipoprotein A-I-induced hPL release^63^. Interestingly, other adipokines also may regulate the molecular mechanisms of hormone release; for example, the stimulation of BeWo cells with adiponectin in the presence of the PKA pathway inhibitor H89 resulted in the inhibition of the positive effect of adiponectin on hCG secretion^64^. Apelin acting by the ERK1/2 pathway decreased the production of each P_4_, E_2_, hCG, hPL, and GH2; in contrast, affecting PKA only lowered the levels of steroids and GH2 in BeWo cells^65^. These results, together with those obtained in this study, show that adipokines play a key role in regulating placental endocrinology and act individually.

Before summarizing our results, we would like to point out that despite the significant data our work provides, we are unfortunately aware of a number of limitations that we were unable to avoid. The main limitation of this study was the availability of placental explants, which resulted in a reduced number of analyses compared with those on the BeWo cells. However, the literature indicates that the number of repetitions we performed (n = 3) is sufficient for placenta studies^66,67^. Despite our efforts, it was not possible to investigate the potential pathway of hCG secretion by visfatin; hence, future studies should focus on explaining this process. Furthermore, future studies should consider the use of syncytialized BeWo cells to better reflect placental hormone secretion under physiological conditions^45^. Finally, it is difficult to discuss potential therapeutic implications in the context of pregnancy pathologies.

In conclusion, visfatin, through various signalling pathways, reduces the secretion of selected hormones in BeWo cells and placentas from normal pregnancies, while having an opposite effect on hPL in PE placentas. Moreover, depending on the dose, visfatin changes the profile of tested hormones in GDM placentas. Additionally, the regulatory role of visfatin on the placental endocrinology that we have demonstrated undoubtedly increases our knowledge about the adipokine itself, its function in reproduction, and the physiology and pathophysiology of the placenta.

## Methods

### In vitro culture

**Fig. 7:**
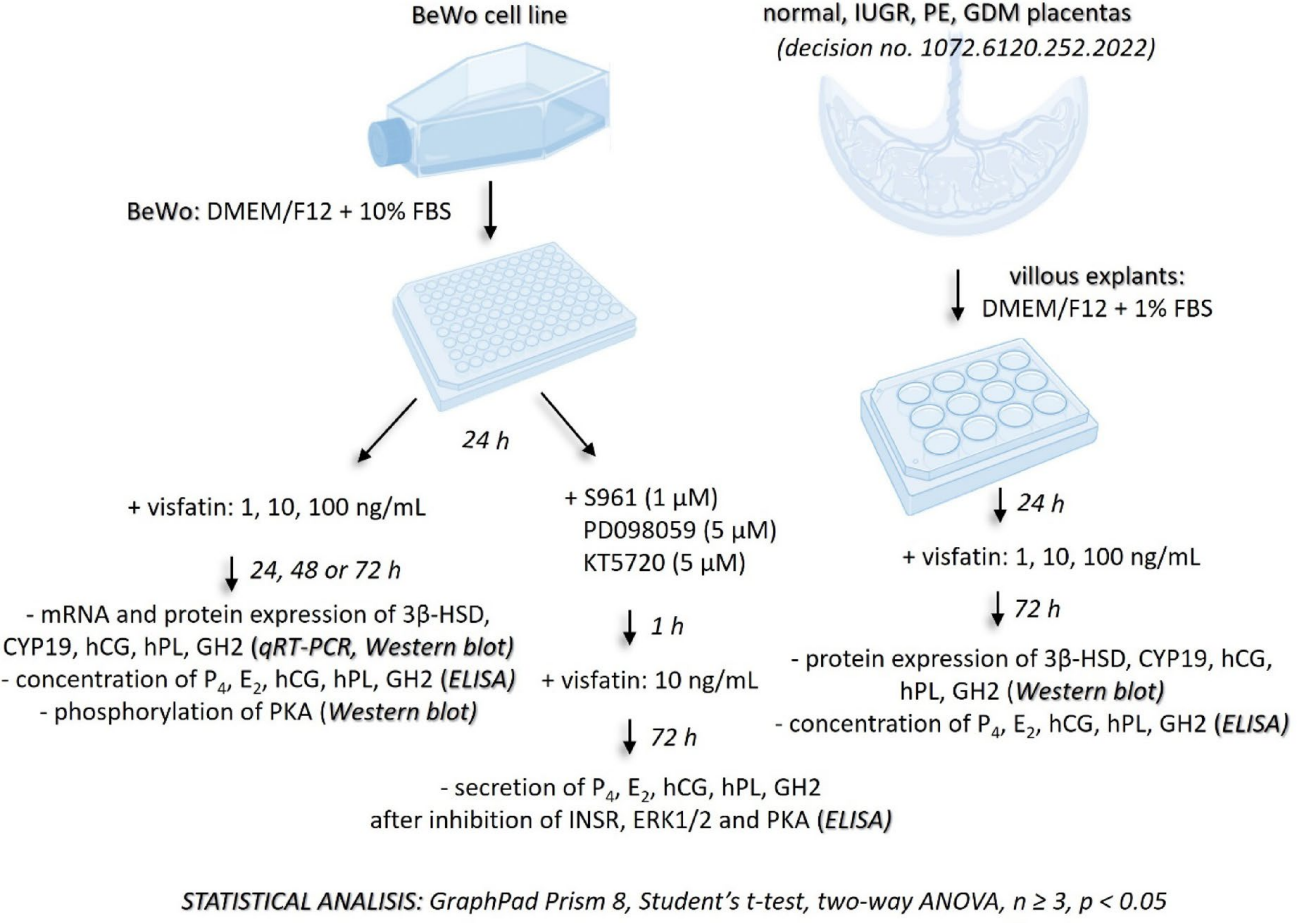
The course of the experiments carried out. IUGR, intrauterine growth restriction; PE, preeclampsia; GDM, gestational diabetes mellitus; DMEM/F12, Dulbecco’s Modified Eagle Medium/Nutrient Mixture F-12; FBS, foetal bovine serum; 3β-HSD, 3β-hydroxysteroid dehydrogenase; P_4_, progesterone; CYP19, aromatase; E_2_, estradiol; hCG, human chorionic gonadotropin; hPL, human placental lactogen; GH2, human placental growth hormone; INSR, insulin receptor; ERK1/2, extracellular signal-activated kinase; PKA, protein kinase A; S961, INSR antagonist; PD098059, ERK1/2 inhibitor, KT5720- PKA inhibitor. The utilised graphics were licenced by BioRender (2025) https://BioRender.com/f09n409.

The BeWo choriocarcinoma cell line (no. CCL-98; American Type Culture Collection, USA, RRID: CVCL_0044) is an optimal model for testing placental endocrinology^65^. The BeWo cells (n = 5) were cultured in Dulbecco’s Modified Eagle Medium/Nutrient Mixture F-12 (DMEM/F12) supplemented with 10% foetal bovine serum (FBS) and 1% L-glutamine until 80–85% confluency was reached. The cells were seeded at a density of 4 × 10^3^ cells/well on 96-well plates. After 24 h, the medium was replaced with DMEM/F12 containing 10% FBS and visfatin at 1, 10, or 100 ng/mL for 24–72 h (Fig. 7). To evaluate the mRNA and protein expression of *HSD3B1/*3β-HSD, *CYP19A1*CYP19*/*, *CGB3/*hCG, *CSH1/*hPL, and *GH2/*GH2, BeWo cells were treated with visfatin (10 ng/mL) for 48 and 72 h. After the treatment with visfatin (1, 10, and 100 ng/mL) for 24, 48, or 72 h, the culture media from the BeWo cells were used to assess the secretion of P_4_, E_2_, hCG, hPL, and GH2. Next, to study the phosphorylation of PKA, BeWo cells were incubated with visfatin (10 ng/mL) for 1, 5, 15, 30, 45, and 60 min. To measure the involvement of INSR, ERK1/2, and PKA in the action of visfatin on placental endocrinology, BeWo cells were treated for 1 h with a pharmacological antagonist of INSR (1 µM, S961), ERK1/2 (5 µM, PD98059), and PKA (5 µM, KT5720); then, visfatin (10 ng/mL) was added for 72 h. The reagent concentrations were selected experimentally and based on^25^.

The placentas (38–40 weeks of gestation) of normal (n = 3) and pathological pregnancies affected by IUGR (n = 3), PE (n = 3), and GDM (n = 3) were obtained from the Clinical Department of Gynaecological Endocrinology, Kraków (Bioethics Committee no. 1072.6120.252.2022) with the consent of the Director of the University Hospital. Each pregnant woman provided informed consent to participate in the study and was selected based on inclusion and exclusion criteria (Table 1). Patients were informed in detail about the aims and scope of the study, including the type of biological material collected and its use for future analysis. The subjects were informed of all potential risks associated with participation in the study, as well as their right to withdraw from it at any stage. They were also informed of the manner in which their personal data would be processed and who would have access to it. The documentation provided to participants was approved by the Bioethics Committee and included the following: (i) information regarding the aforementioned participation, (ii) an informed consent form for participation in the medical experiment, (iii) information regarding the processing of personal data, and (iv) a declaration of acceptance of the terms of civil liability. The documentation encompassed the study’s objectives, methodologies, the utilisation of biological materials, data protection protocols, and the rights of the study participants. The patients were covered by health insurance under contract no. 141.2711.9.2022. The written consent of the partner was not required. Material collection was conducted between 2023 and 2024 by qualified medical personnel in accordance with the protocol that was approved by the Bioethics Committee. The placental samples were classified into control and pathological groups (IUGR, PE, and GDM) by clinicians. The authors received fully anonymised placental tissues with clinical diagnoses subsequent to conducting the necessary tests (Table 1). Immediately following collection, placental villous explants were processed without being stored in a biobank for future use. A priori sample size calculation was not performed. The number of placentas was determined based on the findings of previous similar in vitro studies using human placental explants^66,67^. The placentas were transported to the laboratory within 30 min after gestation in phosphate-buffered saline (PBS) supplemented with 100 IU/mL penicillin and 100 μg/mL streptomycin. Subsequently, explants of placental villi (15 mg wet weight) were isolated and cultured in DMEM/F12 with 10% FBS on 12-well plates^25^. After 24 h of incubation, the medium was replaced with DMEM/F12 containing 1% FBS. Visfatin at doses of 1, 10, or 100 ng/mL was added for 72 h to estimate the protein expression of steroidogenic enzymes (3β-HSD, CYP19), protein hormones (hCG, hPL, GH2), and the secretion of P_4_, E_2_, hCG, hPL, GH2. After the experiments, the waste was disposed of according to Jagiellonian University regulations and an agreement on biomedical waste collection, transport, and disposal.

**Table 1:** List of the criteria for inclusion and exclusion of patients from the study.

Inclusion criteria	Exclusion criteria
Informed consent to participate in the study Patients with a diagnosed normal pregnancy Patients with a diagnosed IUGR Patients with a diagnosed PE Patients with a diagnosed GDM BMI < 25 kg/m^2^ Age between 20 and 40 years	Lack of consent to participate in the study Not pregnant Patients with a history of surgical treatment of ovarian lesions Patients with a history of chemotherapy for oncological indications Patients with a history of using drugs used in oncological chemotherapy for other indications Patients with a history of radiotherapy for oncological indications Patients with a history of radiation for non-oncological indications Patients with a history of immunosuppressive treatment Patients treated in the fertility protection procedure “oncofertility” PATIENTS with a history of treatment or currently treated with systemically administered steroids Neoplastic disease Liver function disorders Kidney function disorders Blood coagulation disorders Dyslipidemia Acute inflammatory processes Psychiatric diseases Type I diabetes or type II diabetes

IUGR, Intrauterine growth restriction; PE, Preeclampsia; GDM, Gestational diabetes mellitus; BMI, Body mass index. Each patient underwent a detailed biochemical and hormonal profile, encompassing a complete blood count, creatinine, lipid profile (total cholesterol, LDL cholesterol, HDL cholesterol, triglycerides). Levels of insulin, follicle-stimulating hormone, luteinizing hormone, prolactin, estradiol, testosterone, sex hormone binding globulin, dehydroepiandrosterone sulfate, 17-hydroxyprogesterone, and cortisol rhythm was studied. Furthermore, the BMI was calculated, and gynaecological and ultrasound examinations were performed to assess placental status. Following a thorough evaluation of the available data, patients were categorised into distinct pathological pregnancy groups, namely IUGR, PE, GDM, or else assigned to the control group, which comprised patients with physiological pregnancies.

BeWo cells and villous explants were cultured using standard incubator conditions: 5% CO2, 95% humidity, at 37 °C. Then, the cells or placental villi were stored at − 70ºC and − 20ºC to analyse mRNA and protein expression, respectively, while the culture media were stored at − 20ºC. Reagents used in these studies were described in Supplementary Table S1.

### Reverse transcription-qPCR

The total isolation of RNA, followed by reverse transcription (1 h, 37 °C), was conducted using the TaqMan Gene Expression Cells-to-CT kit^25^. Subsequently, the cDNA was analysed using the StepOnePlus Real-Time PCR System (Applied Biosystems, Thermo Fisher Scientific) and the TaqMan Gene Expression Assay (Supplementary Table S2) in conjunction with the TaqMan Gene Expression Master Mix containing the reference dye ROX. The reaction was conducted at 50 °C for 2 min, 95 °C for 10 min, and then 40 cycles of 95 °C for 15 s and 60 °C for 60 s. The gene expression was normalized using the reference glyceraldehyde-3-phosphate dehydrogenase (GAPDH) by the 2^−ΔΔ^Ct method^68^.

### Western blot

Protein-equalized lysates (20–40 μg of protein per lane) were loaded and separated using hand-casting 10% polyacrylamide gels^69^. Subsequently, the proteins were transferred to polyvinylidene fluoride (PVDF) membranes using Trans-Blot Turbo Mini PVDF Transfer Packs (BioRad, Hercules, CA, USA). Next, the membranes were blocked for 1 h at room temperature in 0.02 M Tris-buffered saline with 5% bovine serum albumin (BSA) or 5% non-fat dry milk dissolved in 0.1% Tween-20. They were then incubated overnight at 4 °C with the primary antibodies for 3β-HSD, CYP19, hCG, hPL, GH2, pPKA, and tPKA (Supplementary Table S2). Next, the membranes were rinsed in Tris-buffered saline containing 0.1% Tween-20 and incubated with horseradish peroxidase (HRP)—conjugated secondary anti-rabbit or anti-mouse antibodies at room temperature for 1 h. β-actin (ACTB) was employed as a loading control, as it is extensively utilised in studies examining steroidogenic enzymes and placental hormones in trophoblast cells^70,71^. The chemiluminescence signal was visualized using an HRP substrate and the Chemidoc™ XRS + System (BioRad). The densitometry was conducted using the ImageJ software (version 1.51, National Institutes of Health, USA). Representative original blots are presented in Supplementary Fig. S1.

### ELISA

The concentrations of hormones were examined using commercial ELISA kits for P_4_, E_2_, hCG, and hPL, sourced from DRG, and the GH2 was sourced from Fine Test (Supplementary Table S2), following the manufacturer’s protocol. The absorbance was measured in a Varioskan LUX Multimode Microplate Reader using SkanIt software 6.1.1 (Thermo Fisher Scientific) at a wavelength of 450 nm.

### Statistical analysis

The experiments were performed at least three times (BeWo cell line: n = 5; terminal placentas: n = 3), and the results were subjected to statistical analysis using Student’s t-test, two-way ANOVA, and followed by Sidak’s multiple comparisons test in GraphPad Prism 8 (GraphPad Software, USA). The normality of the data distribution was determined using the Shapiro–Wilk test. Figures present the results as the mean ± standard error of the mean (SEM). Statistical significance was shown using or asterisks * (*p* < 0.05), ** (*p* < 0.01), and *** (*p* < 0.001). The absence of asterisks indicates the absence of significant differences.

## Supplementary Information

Below is the link to the electronic supplementary material.

Supplementary Material 1

## Data Availability

All the data generated or analyzed during this study are included in this published article.
