# Supplementary material for: VISFATIN: a novel modulator of placental endocrinology under physiological and pathological conditions—in vitro studies involving INSR, ERK1/2, and PKA signalling pathways
Source: Sci Rep. 2026 May 24;16:23781. doi: 10.1038/s41598-026-52422-w (PMC13434048; doi:10.1038/s41598-026-52422-w)

Supplementary Table 1. List of reagents used in studies, along with catalog numbers and concerns.

***IN VITRO* CULTURE**		
**Name**	**Catalog numbers**	**Concerns**
Dulbecco’s Modified Eagle Medium/Nutrient Mixture F-12 (DMEM/F12)	no. 21041025	Thermo Fisher Scientific (Waltham, MA, USA)
phosphate-buffered saline (PBS)	no. 14190250	
trypsin	no. 26616	
PD98059	no. 1213	Sigma-Aldrich (Saint Louis, MO, USA)
KT5720	no. K3761	
foetal bovine serum (FBS)	no. F9665	BioWest (Nuaillé, France)
Visfatin	no. 8424-VF-050	Bio-Techne (Minneapolis, MN, USA)
S961	no. 051-86	TargetMol Chemicals (Wellesley Hills, MA, USA)
**RT qPCR**		
**Name**	**Catalog numbers**	**Concerns**
TaqMan Gene Expression Cells-to-CT kit	no. AM1728	Thermo Fisher Scientific (Waltham, MA, USA)
TaqMan Gene Expression Master Mix containing the reference dye ROX	no. 399002	
**WESTERN BLOTTING**		
**Name**	**Catalog numbers**	**Concerns**
Laemmli buffer	no. 38733	Sigma-Aldrich (Saint Louis, MO, USA)
Immobilon Western Chemiluminescent Horseradish Peroxidase (HRP) Substrate	no. WBKLS0500	
polyvinylidene fluoride (PVDF) membranes	no. IPVH00010	
glycine	no. GLN001	BioShop (Burlington, Canada)
Tris	no. TRS001	
Tween 20	no. TWN510.500	
bovine serum albumin (BSA)	no. ALB001.500	

Supplementary Table 2. Specifications of the TaqMan's used in the RT-qPCR (A), primary and secondary antibodies used in the Western blotting (B), and ELISA kits (C). *HSD3B1*/3β-HSD- 3β-hydroxysteroid dehydrogenase, *CYP19A1*/CYP19- aromatase, *CGB3*/hCG- human chorionic gonadotropin, *CSH1*/hPL- human placental lactogen, *GH2*/GH2- human placental growth hormone, pPKA/tPKA- phosphorylated/total protein kinase A, P_4- progesterone, E_2- estradiol.

**(a) SPECIFICATIONS OF THE TAQMAN'S USED IN THE RT-qPCR ANALYSIS**			
**Genes**	**Name**	**Assay sequences**	**Reference sequences**
HSD3B1	3β-hydroxysteroid dehydrogenase	Hs04194787_g1	NM_000862.2
CYP19A1	aromatase	Hs00903411_m1	NM_000103.3
CGB3	human chorionic gonadotropin	Hs00361224_gH	NM_000737.3
CSH1	human placental lactogen	Hs04190926_gH	NM_001317.5
GH2	human placental growth hormone	Hs07291772_g1	NM_002059.4
GAPDH	**glyceraldehyde 3-phosphate dehydrogenase**	Hs02786624_g1	NM_001256799.2

**(b) SPECIFICATIONS OF THE ANTIBODIES USED IN THE WESTERN BLOTTING ANALYSIS**					
**Name**	**Host species**	**Antibody type**	**Catalog numbers and concerns**	**Research Resource Identifiers**	**Dilutions**
PRIMARY ANTIBODIES					
anti-3β-HSD	mouse	monoclonal	no. ab75710, Abcam	AB_2631971	1:1000
anti-CYP19	rabbit	polyclonal	no. PA1-21398, Thermo Fisher Scientific	AB_2088676	1:200
anti-hCG	rabbit	polyclonal	no. ab53087, Abcam	AB_870731	1:500
anti-hPL	rabbit	polyclonal	no. ab15554, Abcam	AB_301963	1:500

anti-GH2	mouse	monoclonal	no. ab9822, Abcam	AB_296648	1:500
anti-p-PKA	rabbit	polyclonal	no. ab5815, Abcam	AB_305132	1:500
anti-t-PKA	rabbit	polyclonal	no. ab187515, Abcam	AB_3665519	1:500
anti-ACTB	mouse	monoclonal	no. A5316, Sigma-Aldrich	AB_476743	1:1000
SECONDARY ANTIBODIES					
anti-rabbit	goat	polyclonal	no. 7074, Cell Signaling	AB_2099233	1:1000
anti-mouse	horse	polyclonal	no. 7076, Cell Signaling	AB_330924	1:1000

(c) SPECIFICATION OF THE ELISA KITS USED IN THE ANALYSIS

**Hormone**	**Catalog numbers and concerns**	**Sensitivity of assay**	**Ranges of assay**	**Intra-experiment coefficients of variation**	**Inter-experiment coefficients of variation**
P_4	EIA-1516, DRG	0.045 ng/mL	0-40 ng/mL	6.99%	4.34%
E_2	EIA-2693, DRG	9.714 pg/mL	0-2000 pg/mL	2.71%	6.72%
hCG	EIA-1911, DRG	< 1 mIU/mL	0- 200 mIU/ml	4.00%	7.30%
hPL	EIA-1283, DRG	0.043 mg/L	0-20 mg/L	5.55%	7.14%
GH2	EH3133, Fine Test	46.875 pg/mL	78.125-5000 pg/mL	4.85%	4.74%

Supplementary Figure S1. Representative original blots.

Representative original blots to protein expression of 3β-HSD, CYP19, and ACTB in BeWo cells (Fig. 1a, 1b).

Fig. 1b

3β-HSD: 42 kDa

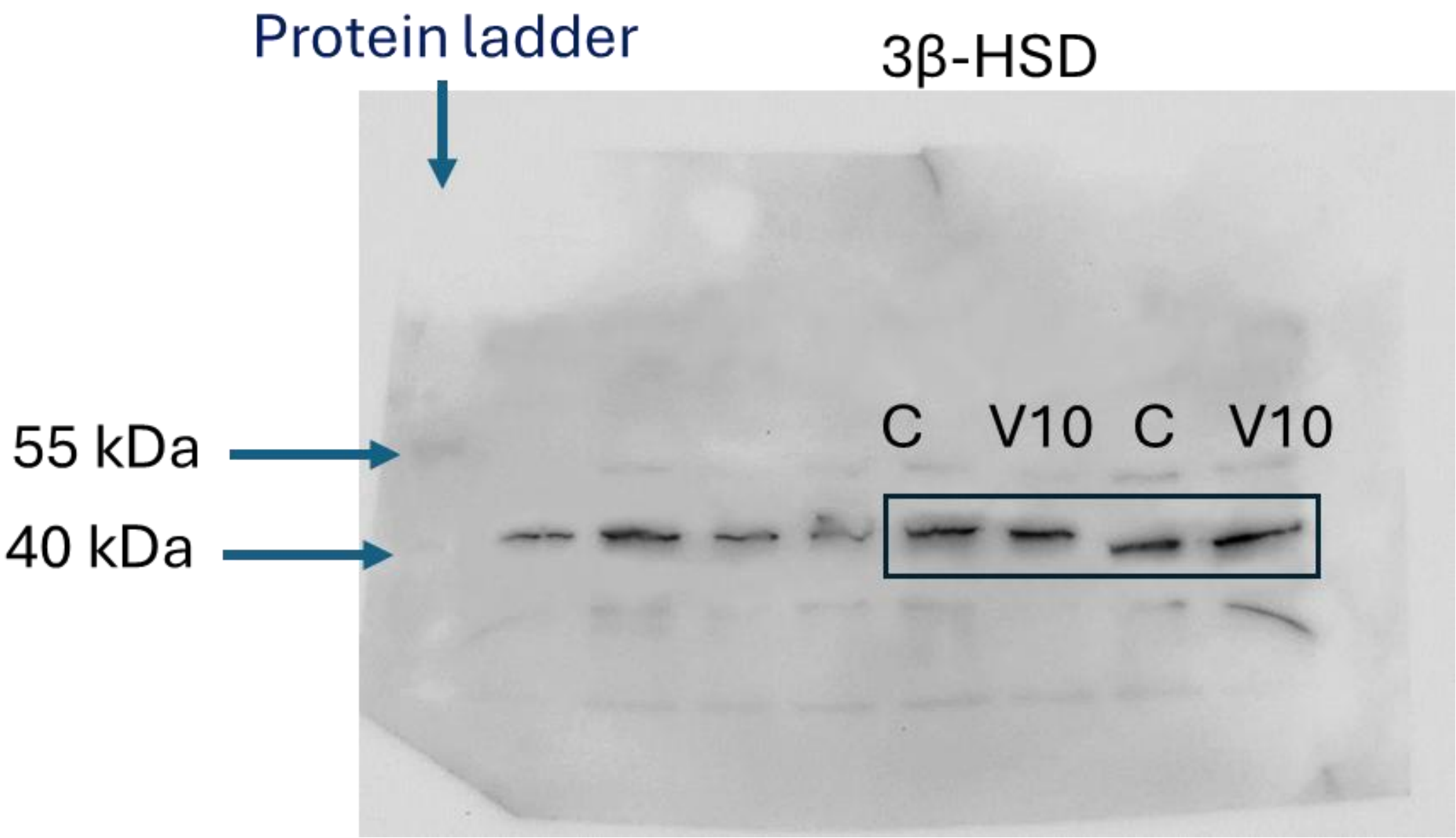

ACTB: 42 kDa

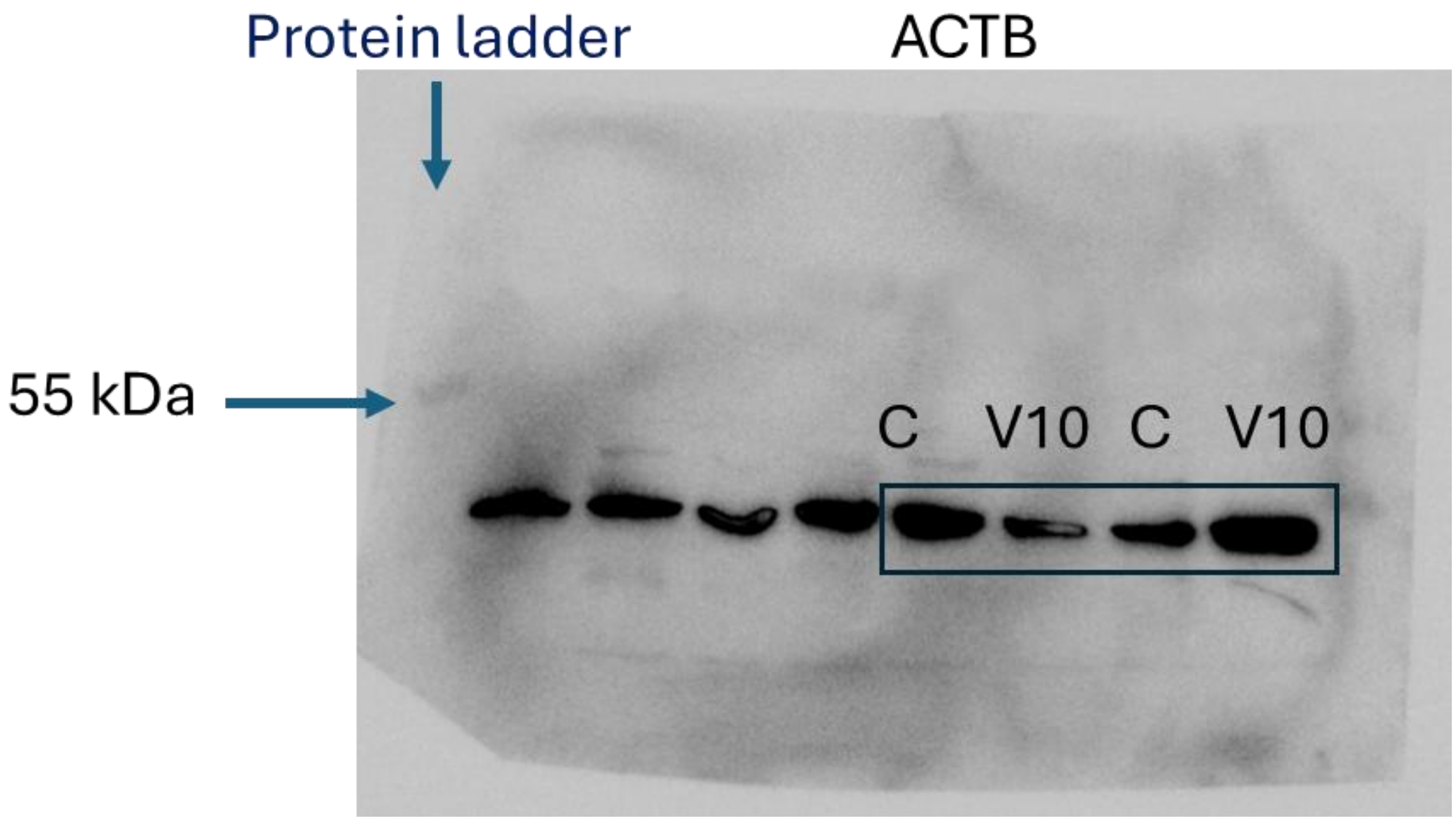

Fig. 1e

CYP19: 30 kDa

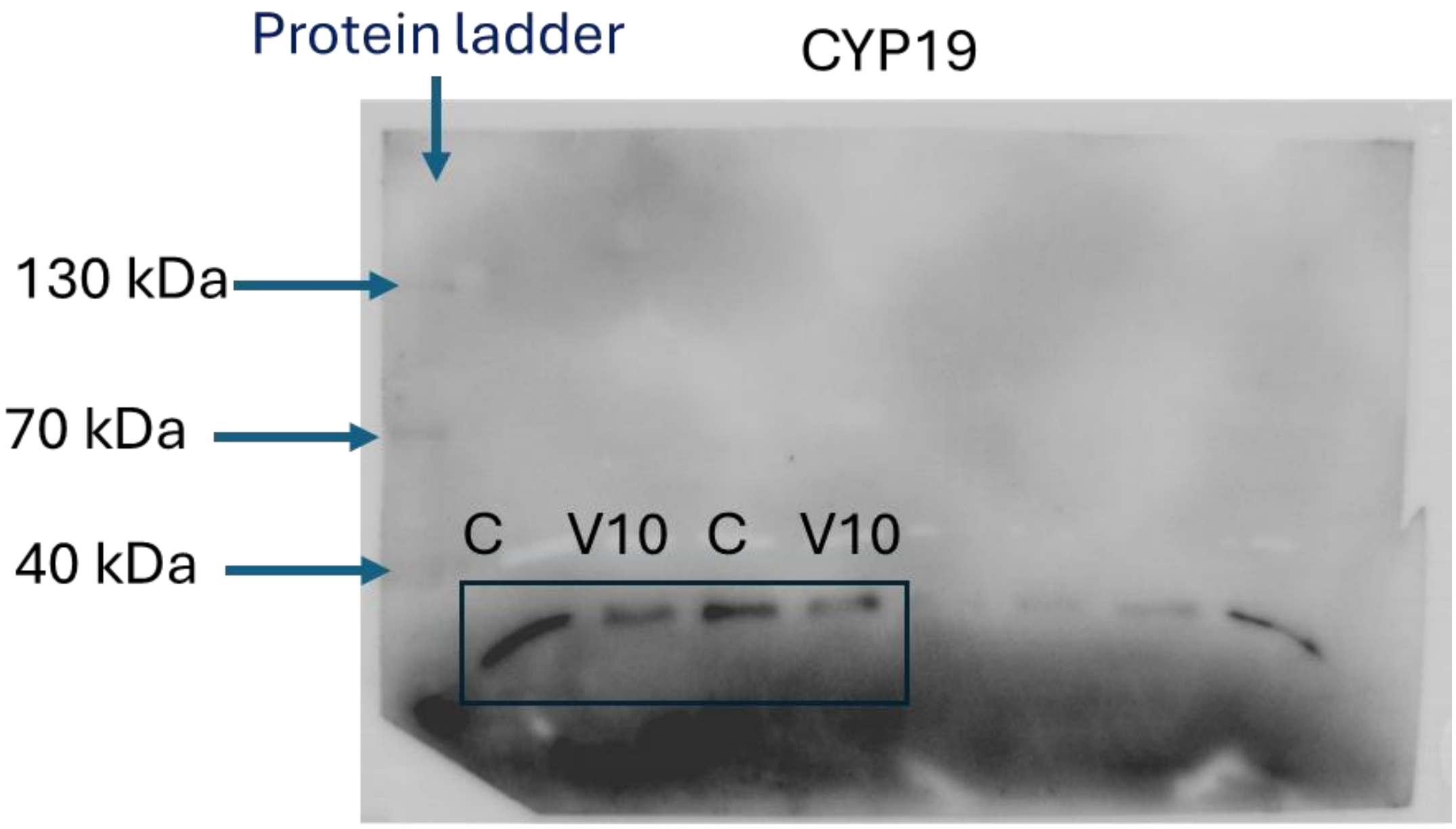

ACTB: 42 kDa

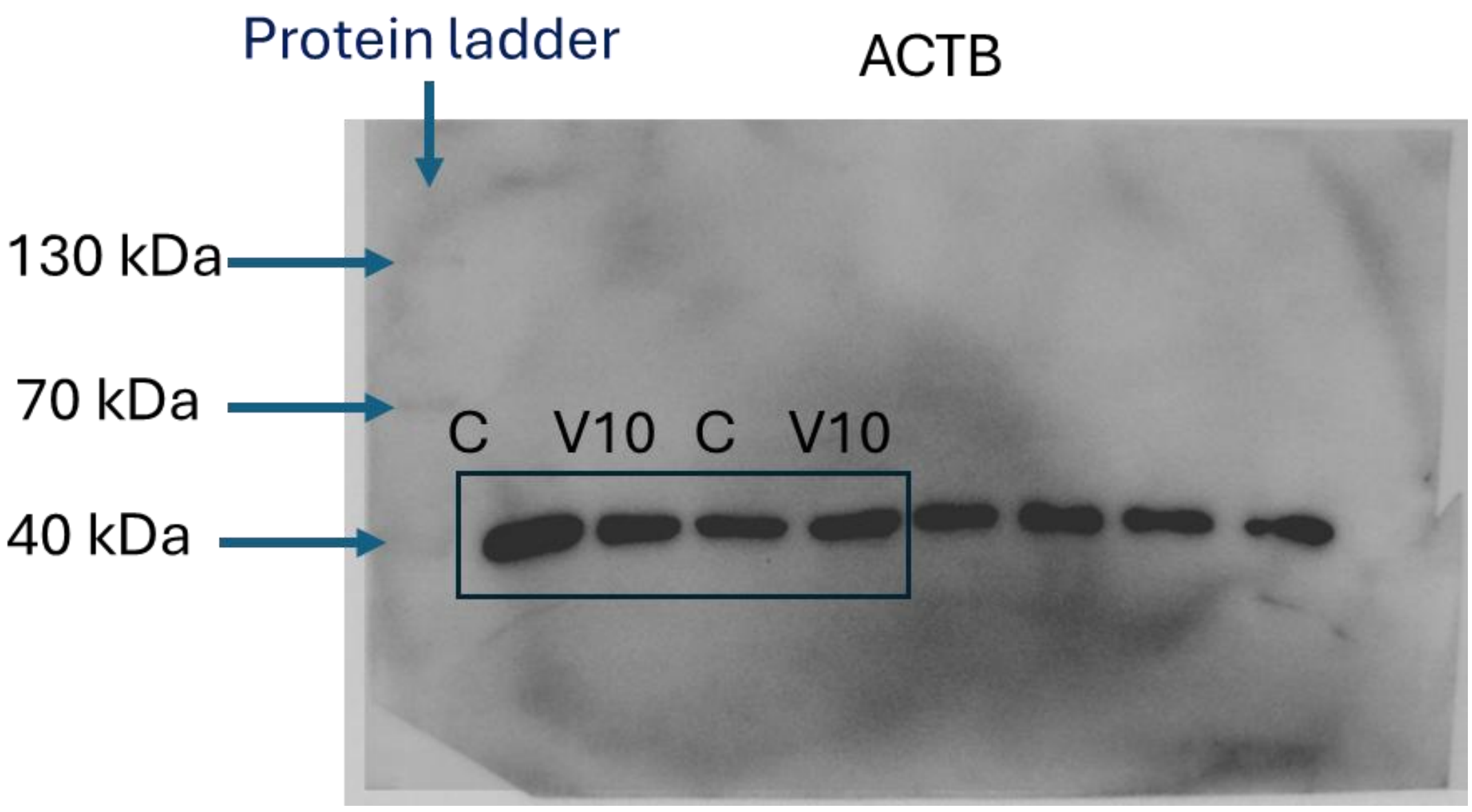

Representative original blots to protein expression of hCG, hPL, GH2 and ACTB in BeWo cells (Fig. 2a, 2a, 2c).

Fig. 2b

hCG: 22 kDa

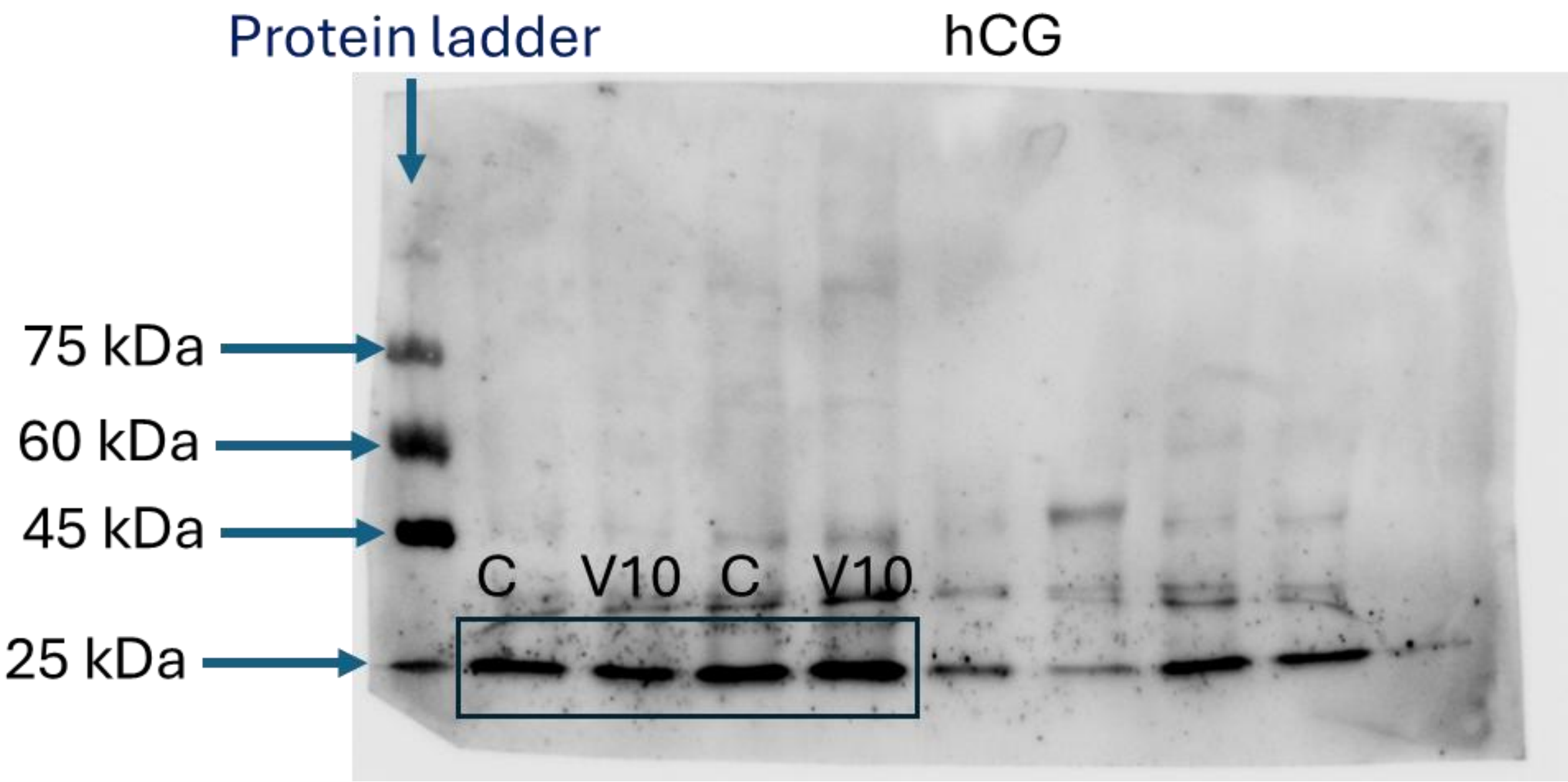

ACTB: 42 kDa

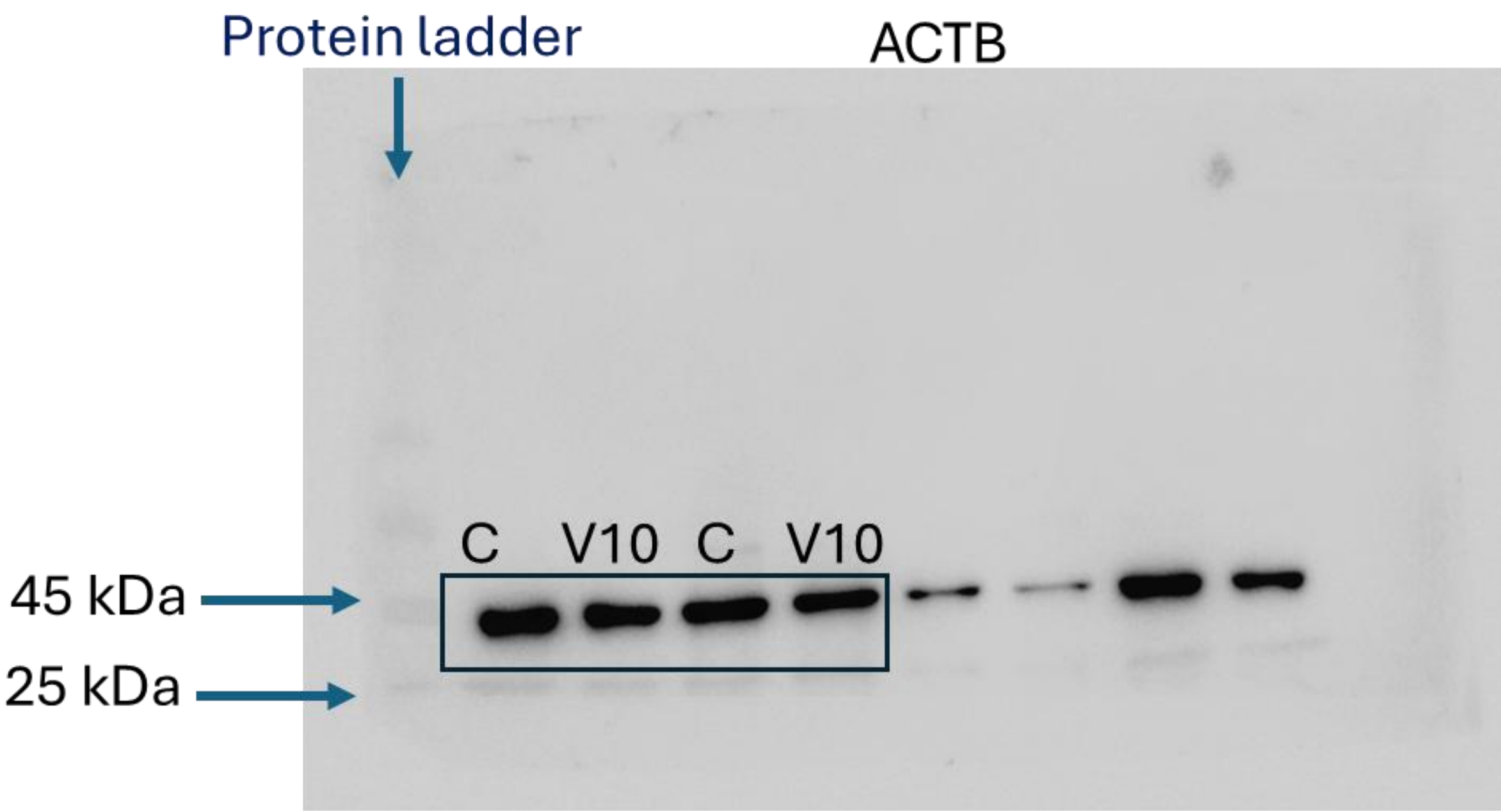

Fig. 2e

hPL: 42 kDa

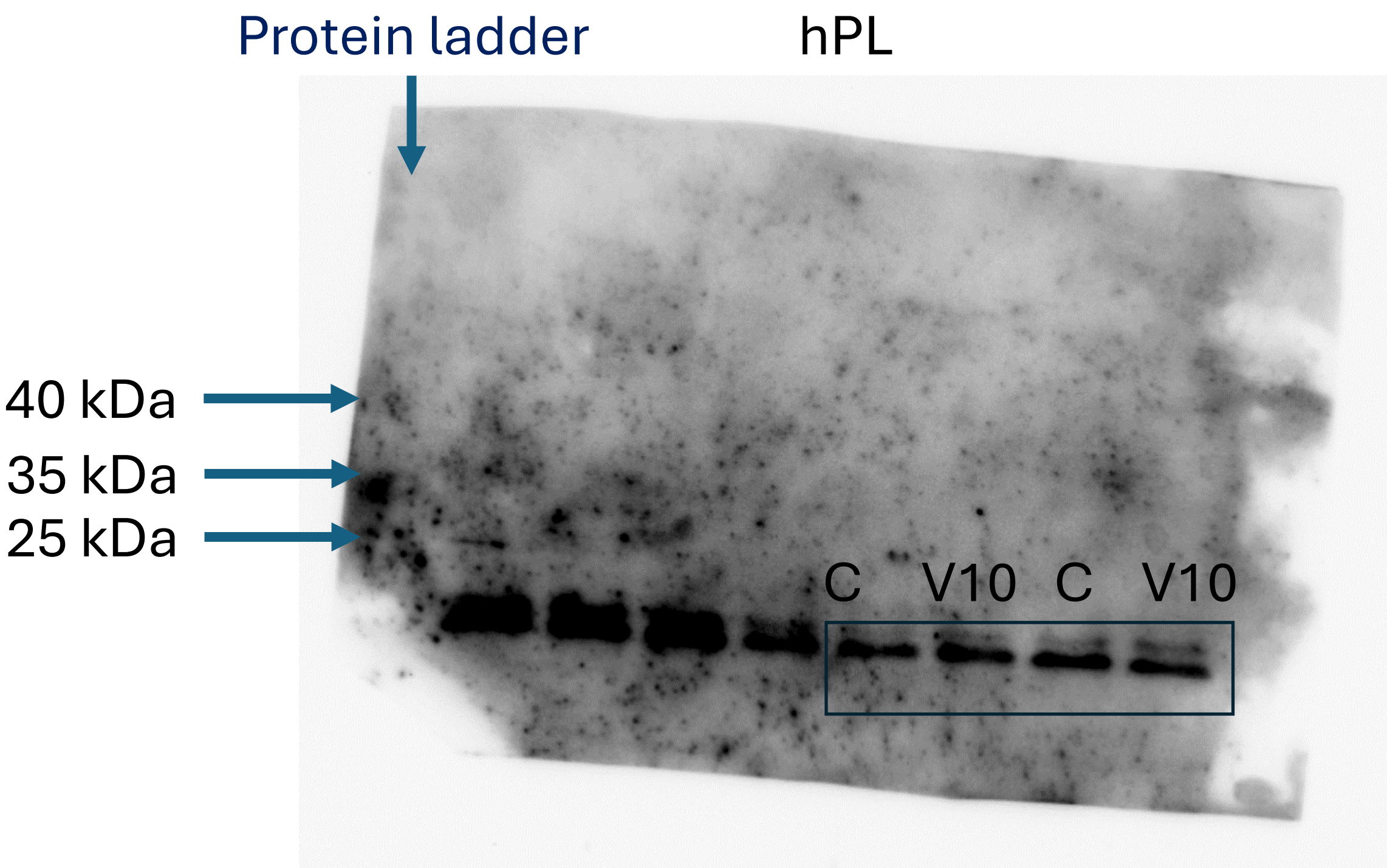

ACTB: 42 kDa

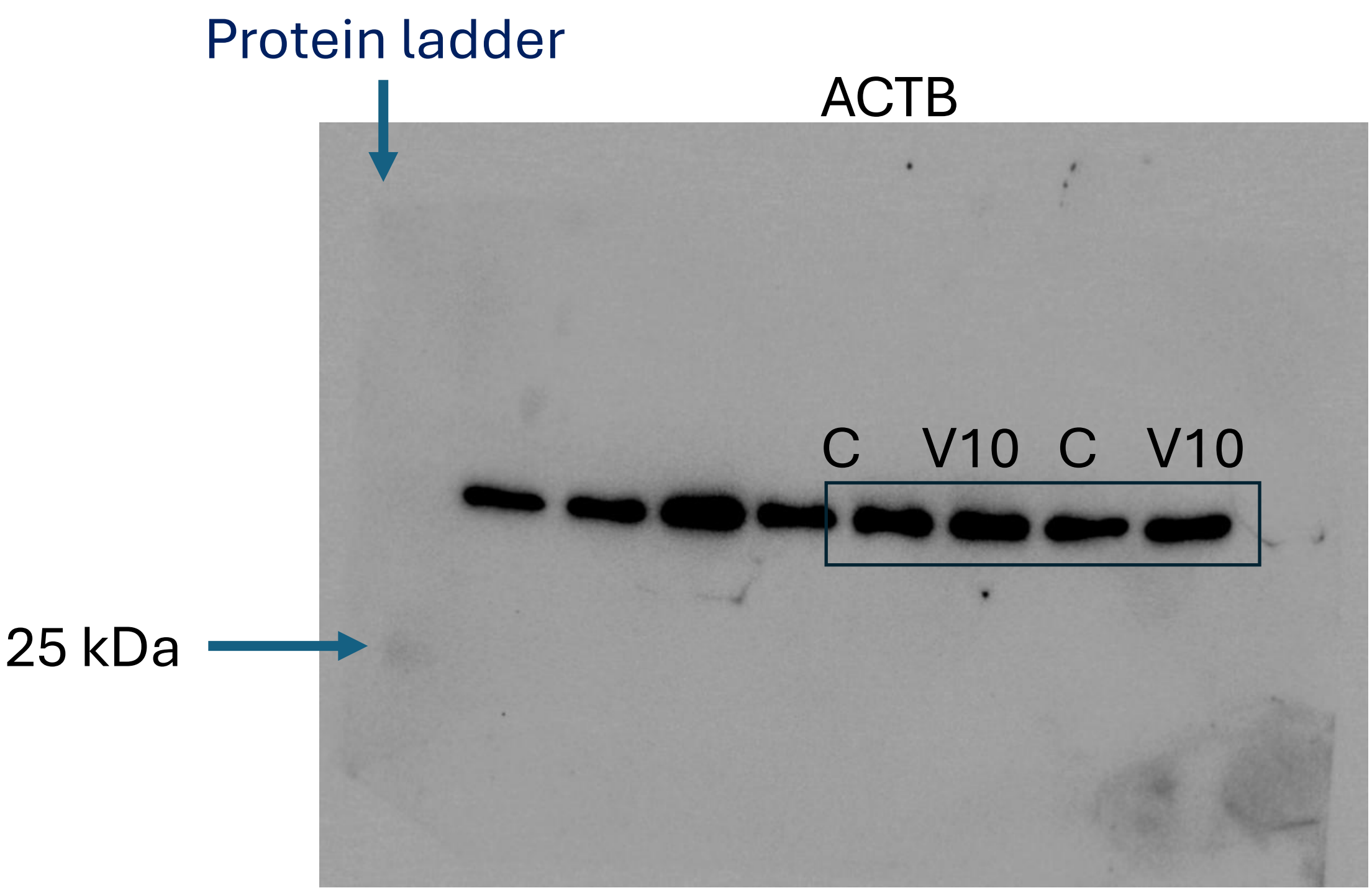

Fig. 2h

GH2: 25 kDa

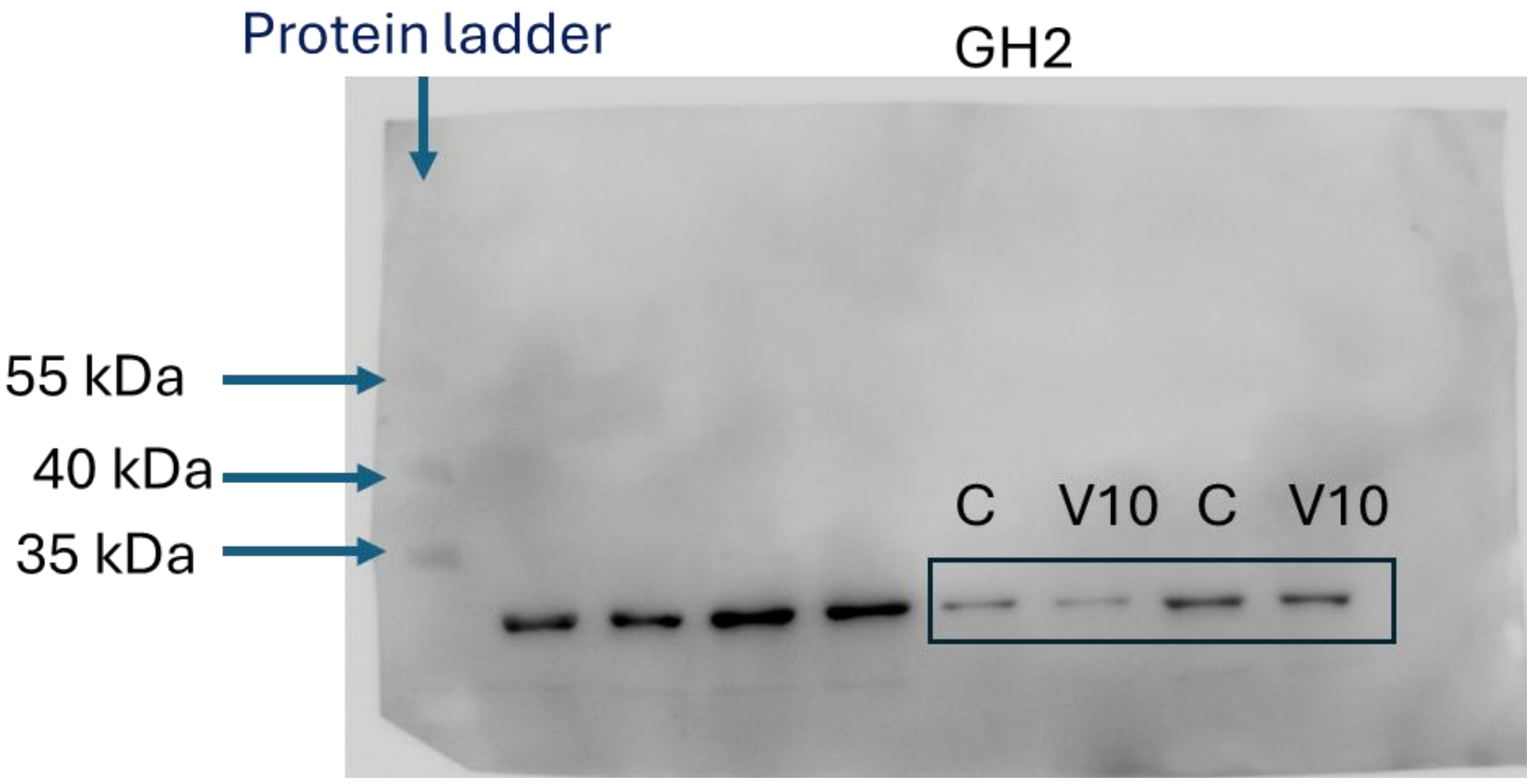

ACTB: 42 kDa

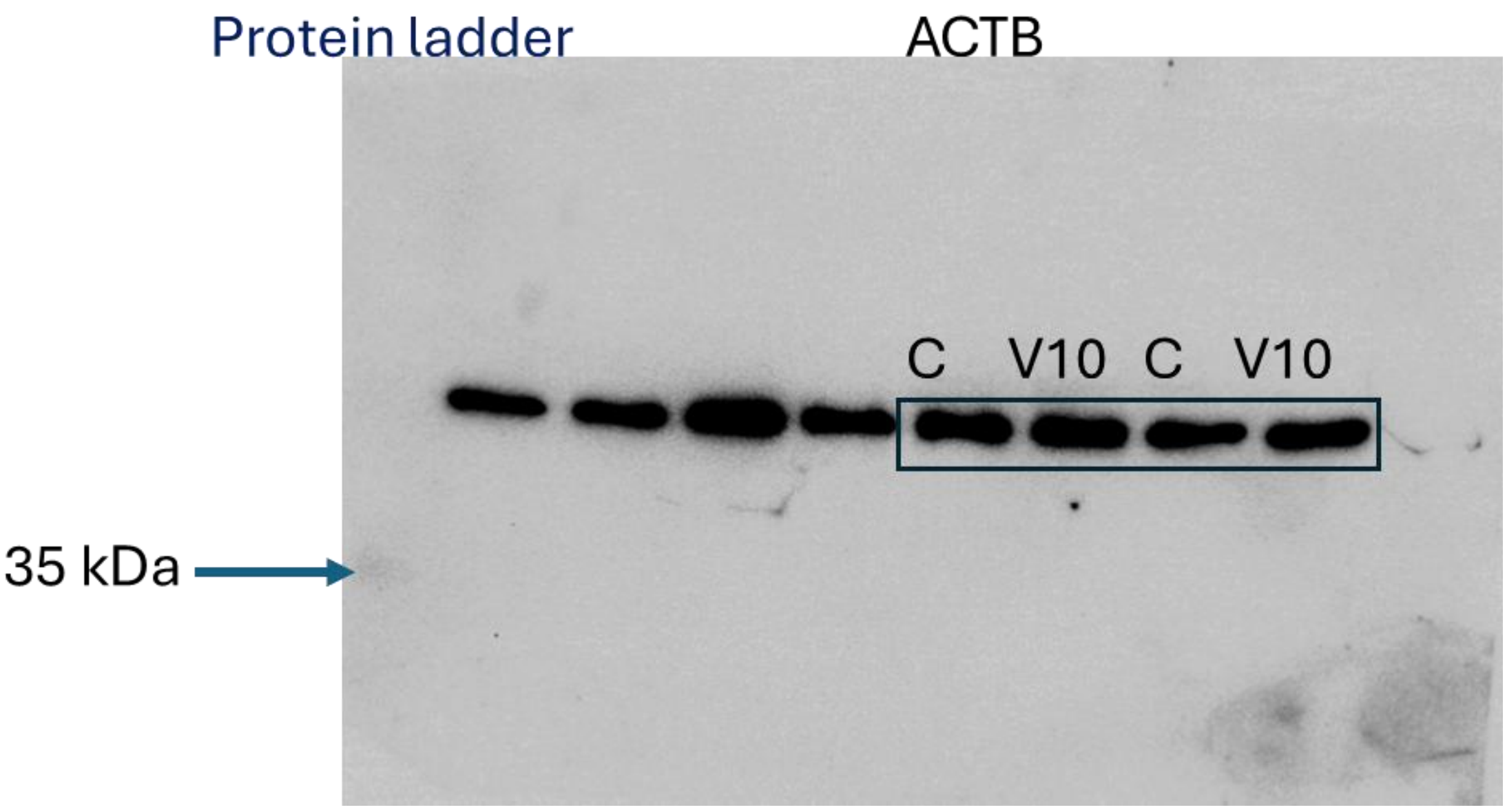

Representative original blots to protein expression of 3β-HSD, CYP19, and ACTB in human placenta (Fig. 3a, 3b).

Fig. 3a

3β-HSD: 42 kDa

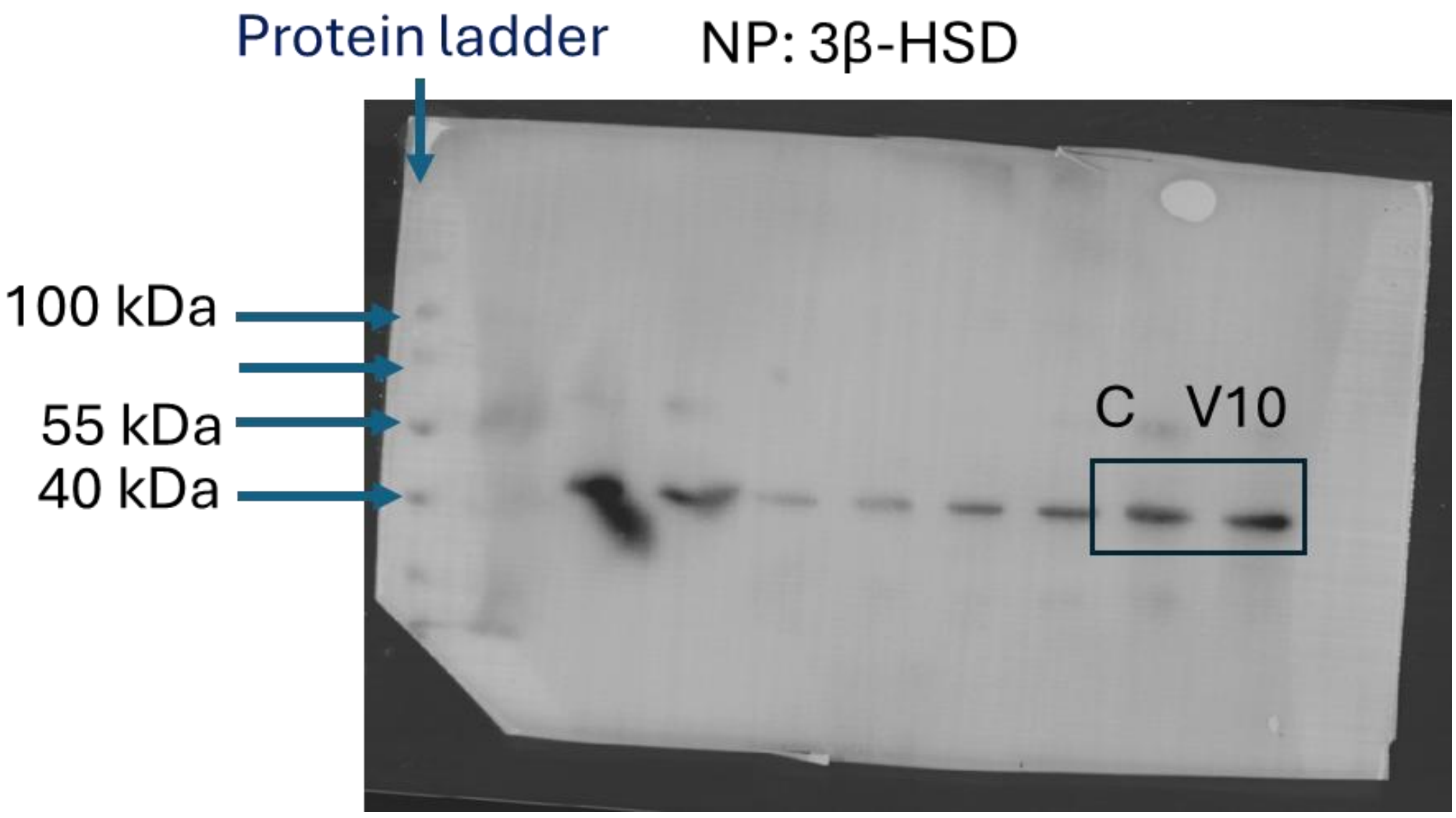

ACTB: 42 kDa

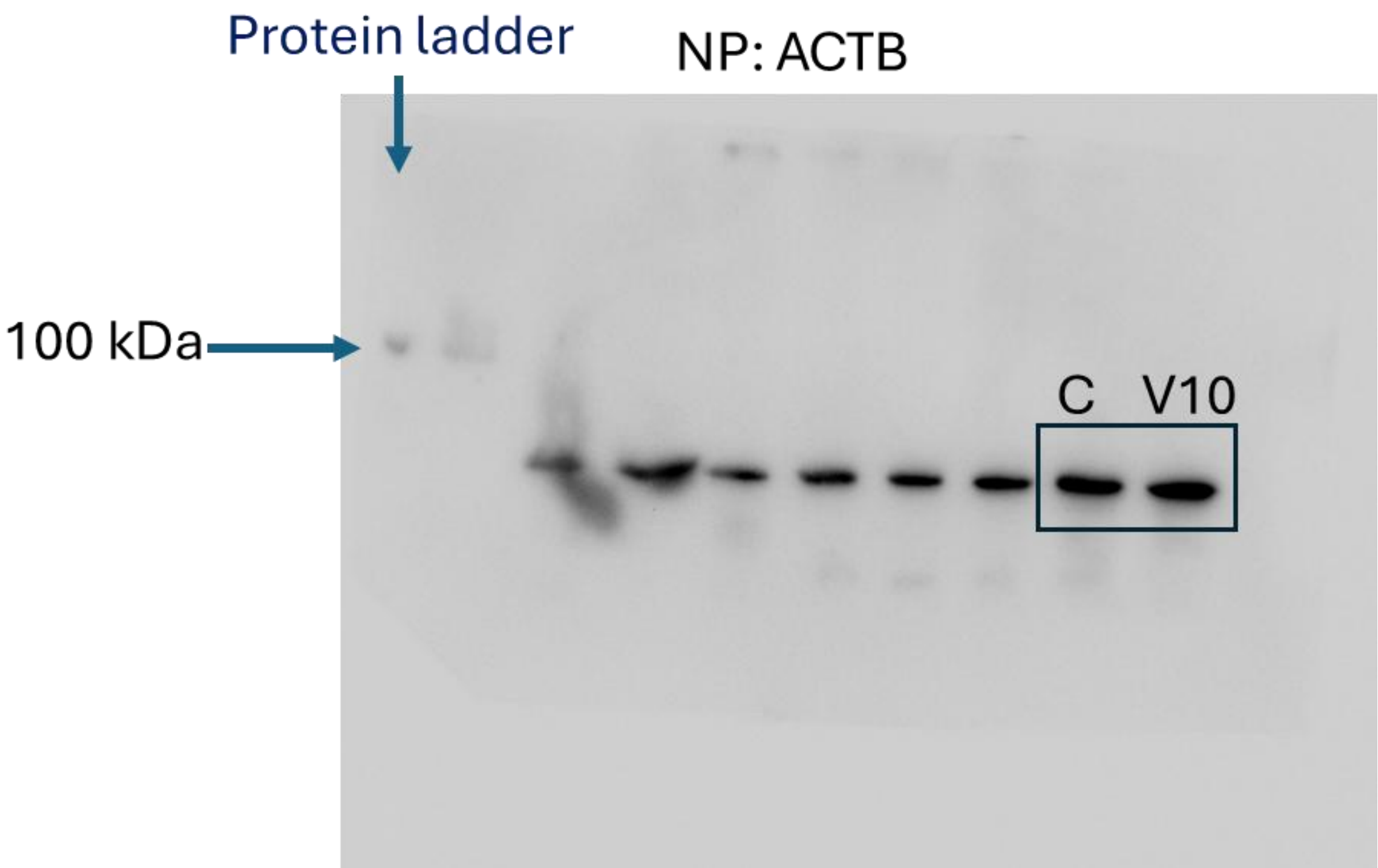

3β-HSD: 42 kDa

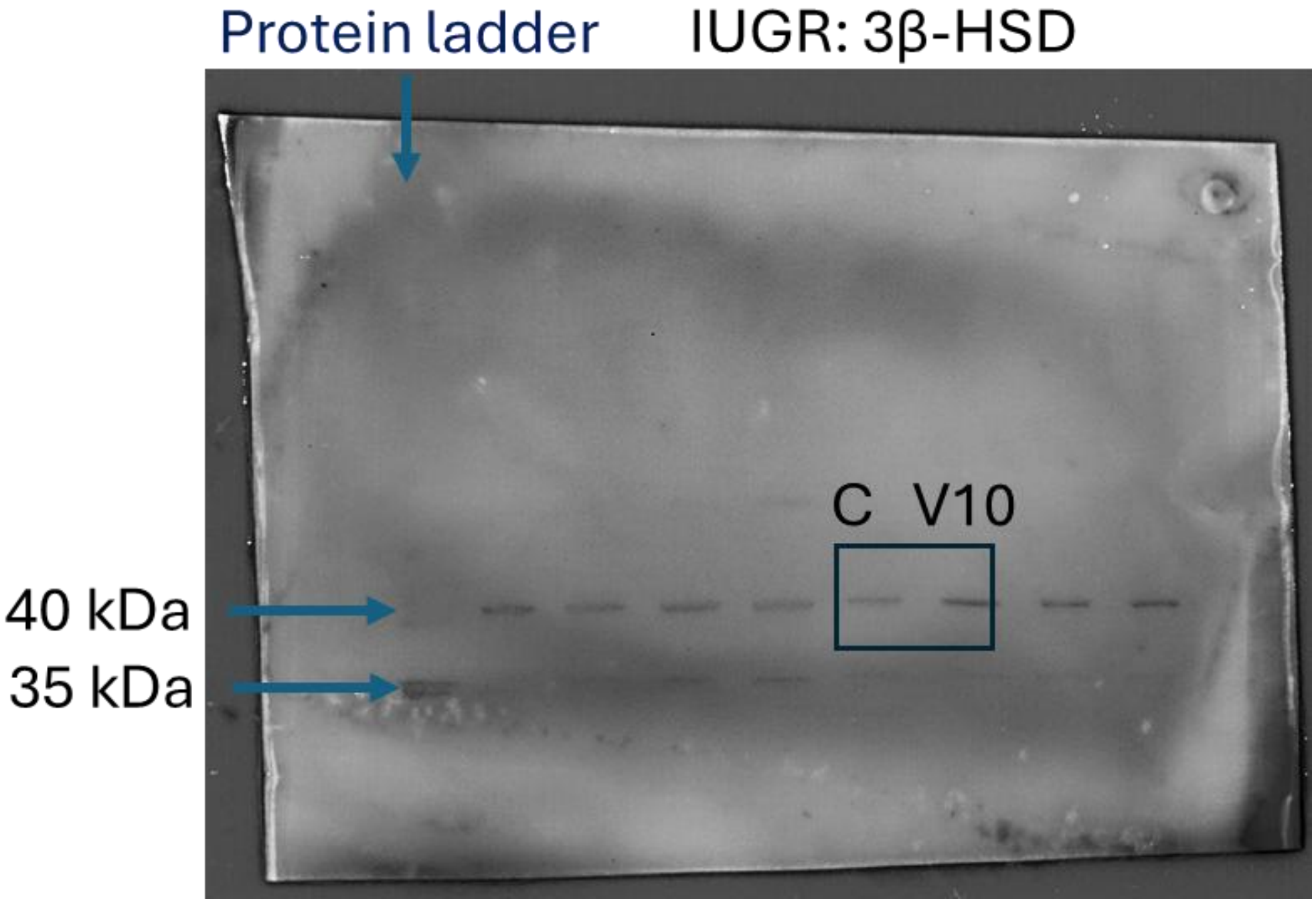

ACTB: 42 kDa

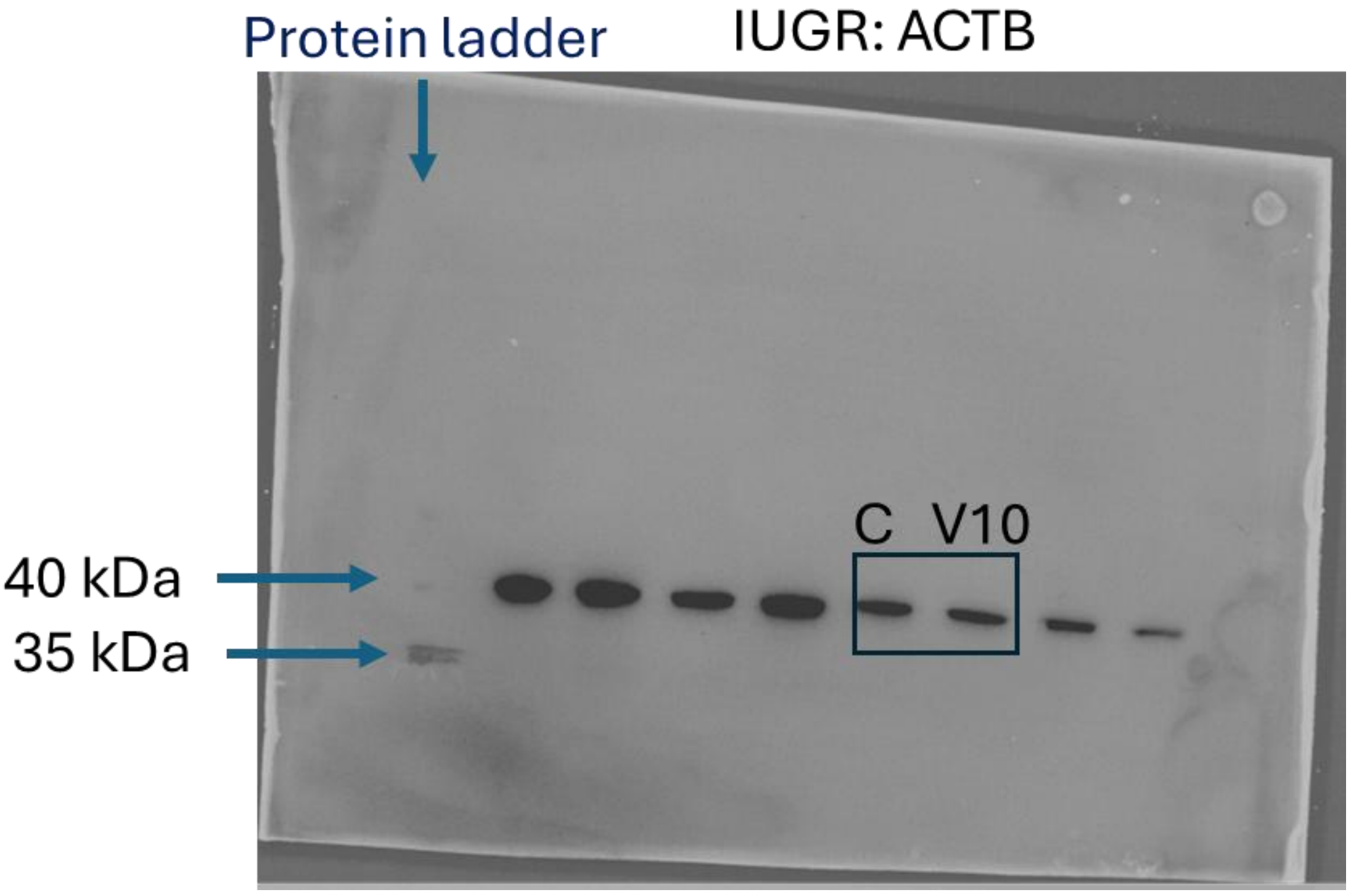

3β-HSD: 42 kDa

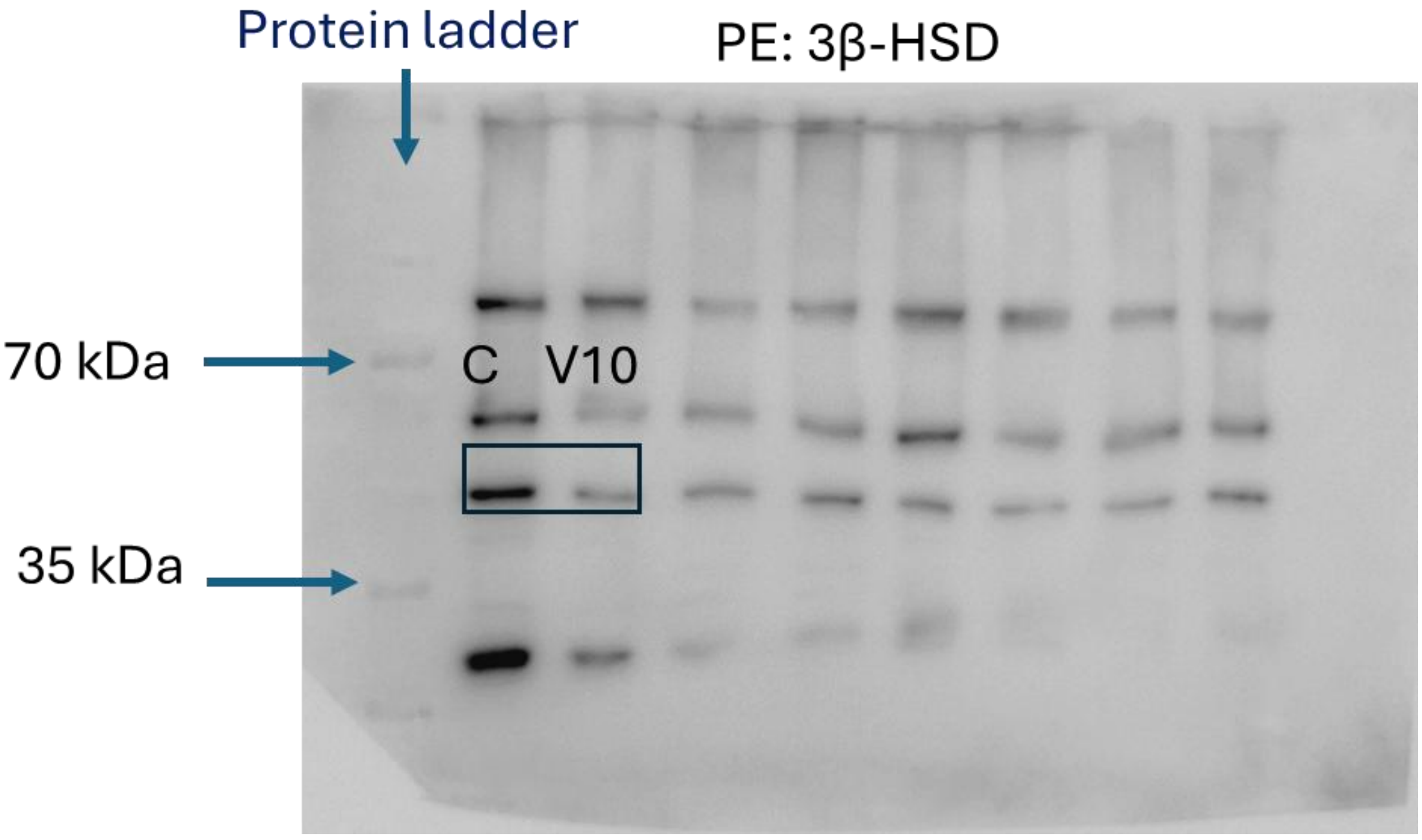

ACTB: 42 kDa

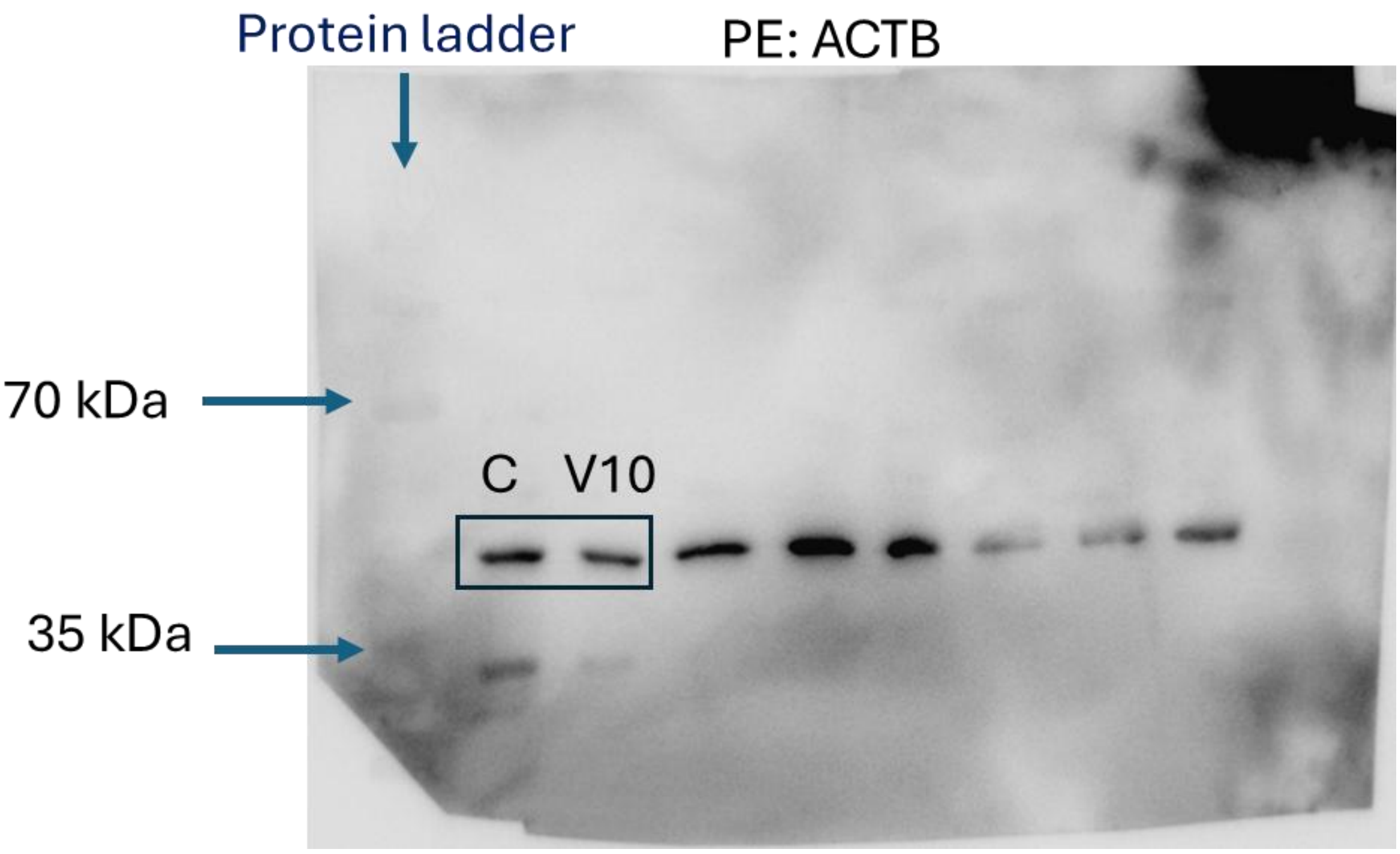

3β-HSD: 42 kDa

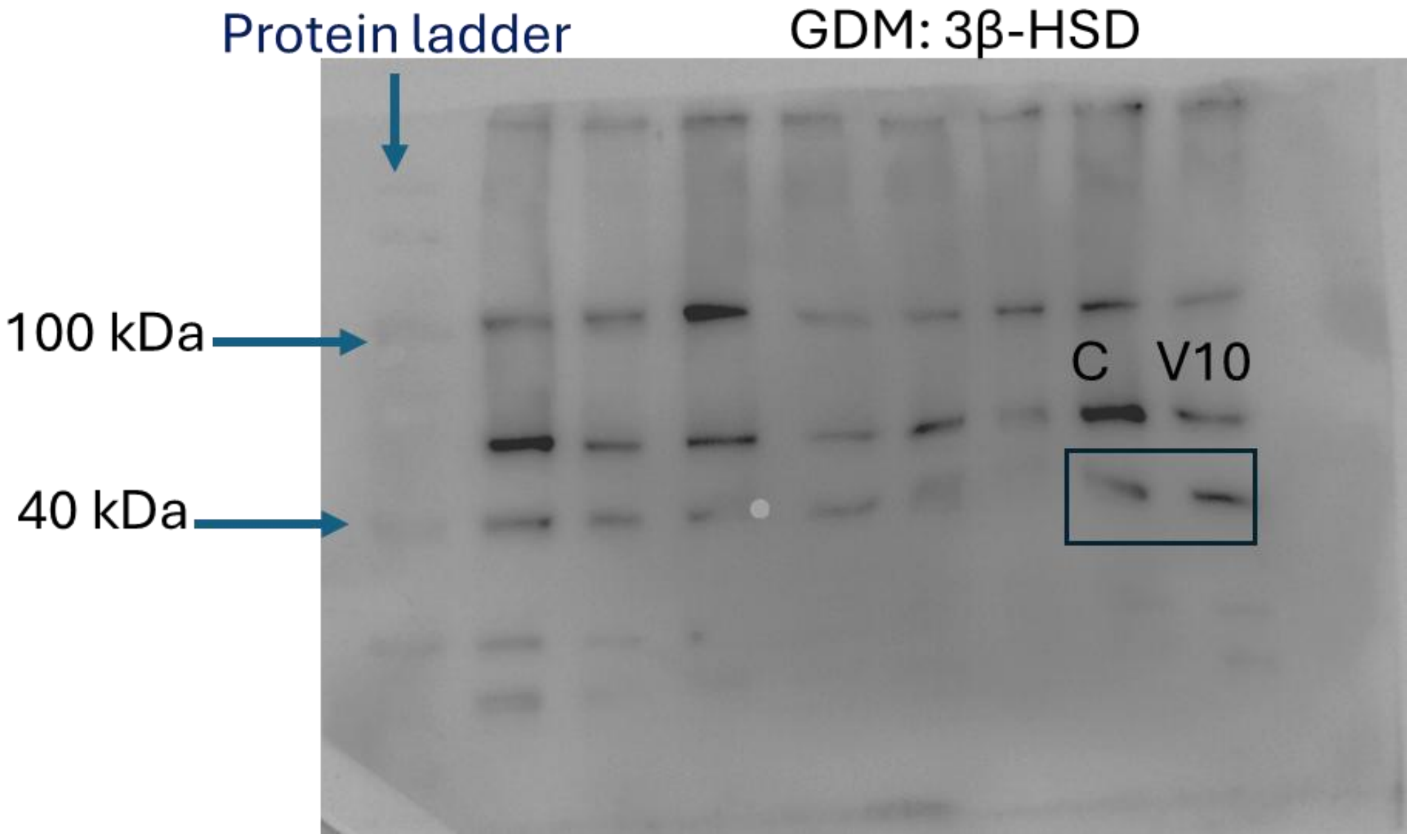

ACTB: 42 kDa

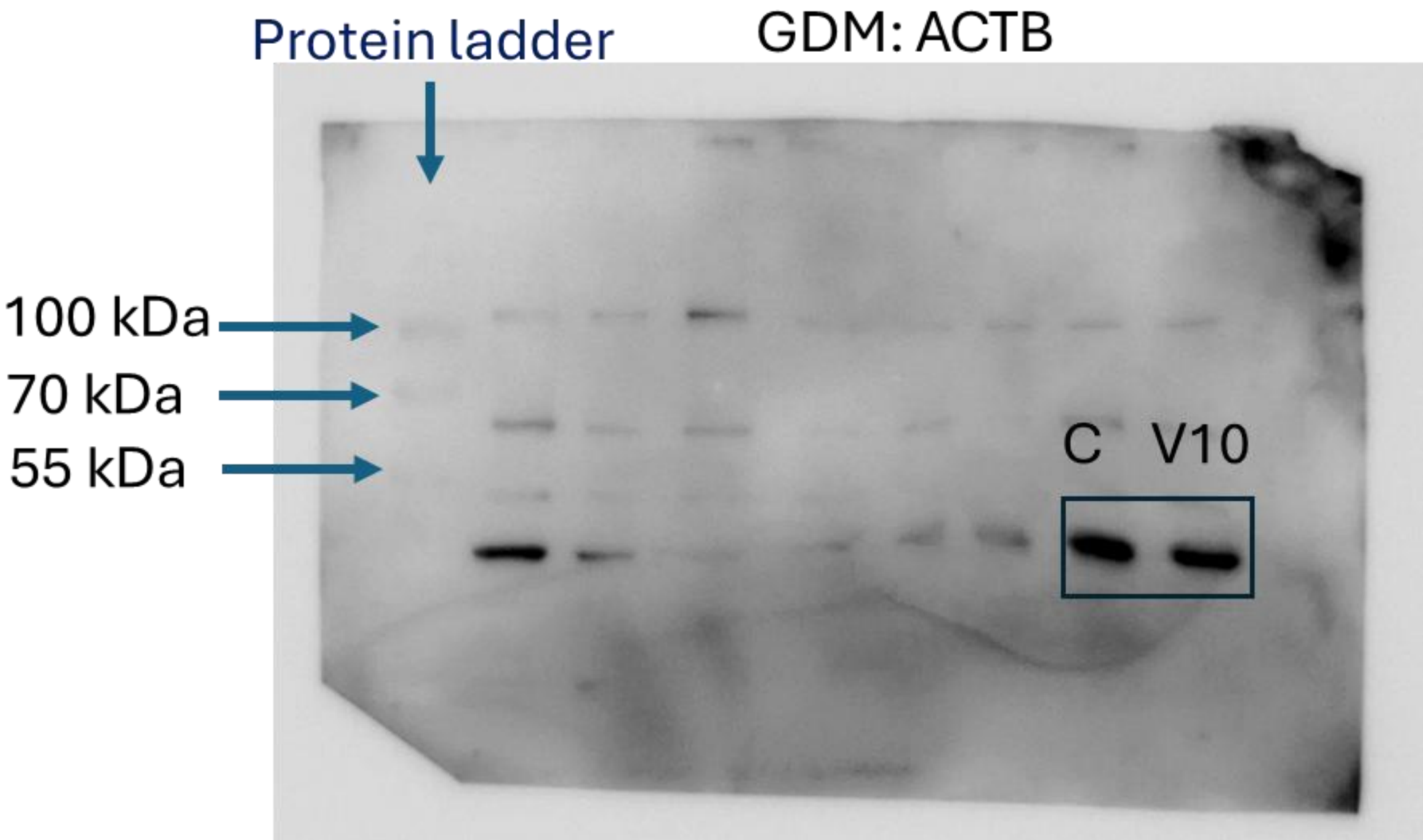

Fig. 3c

CYP19: 30 kDa

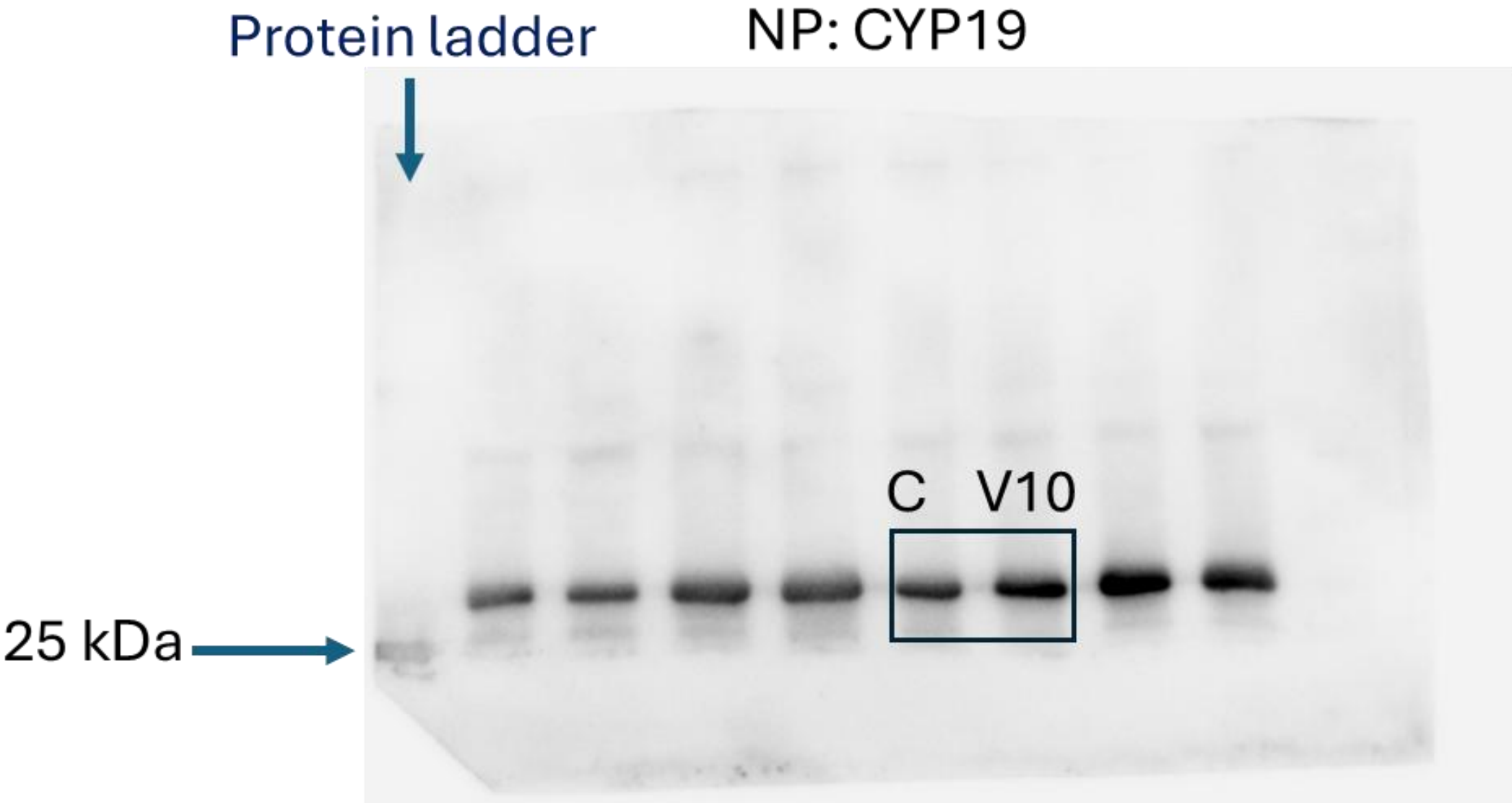

ACTB: 42 kDa

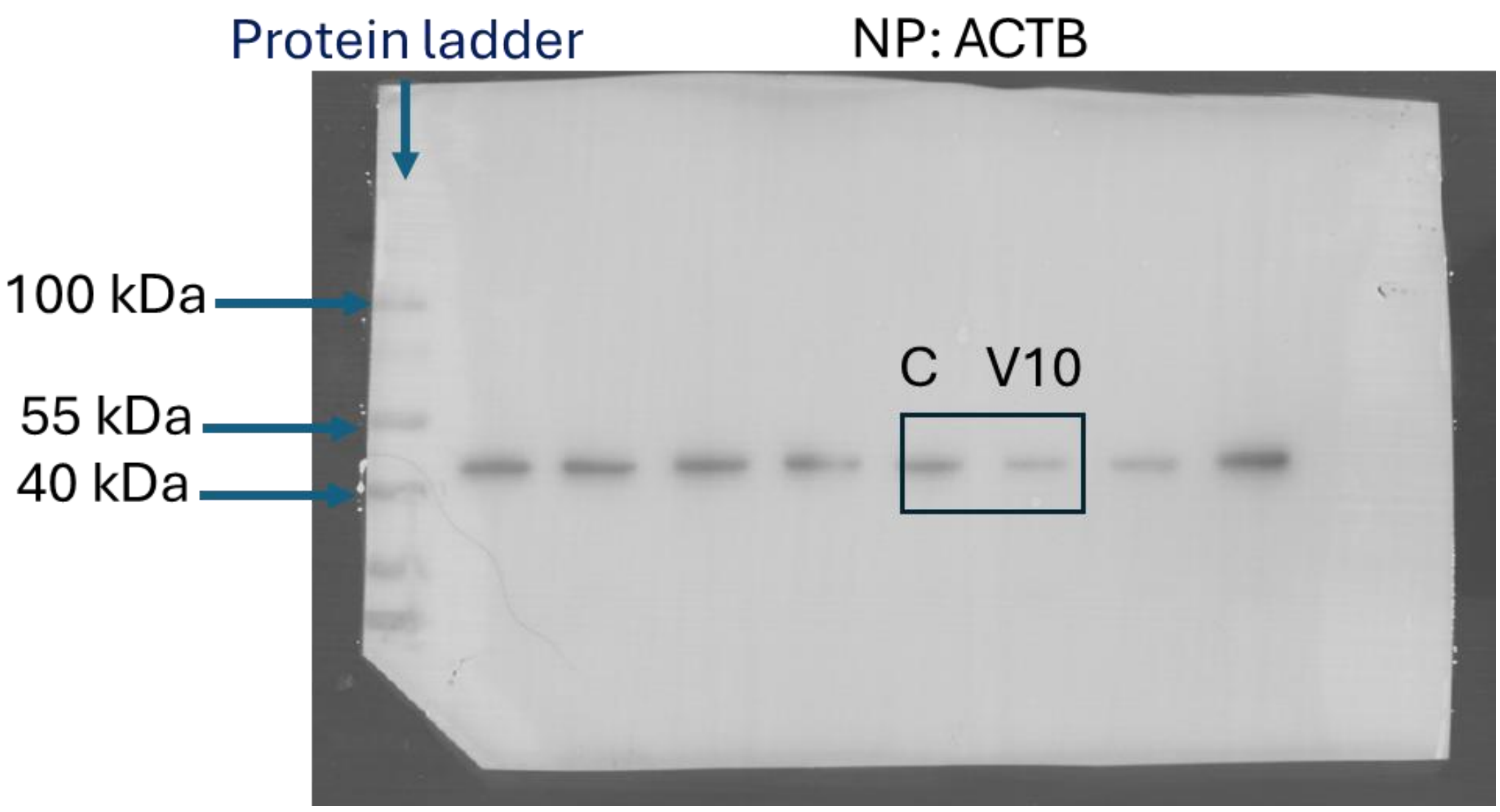

CYP19: 30 kDa

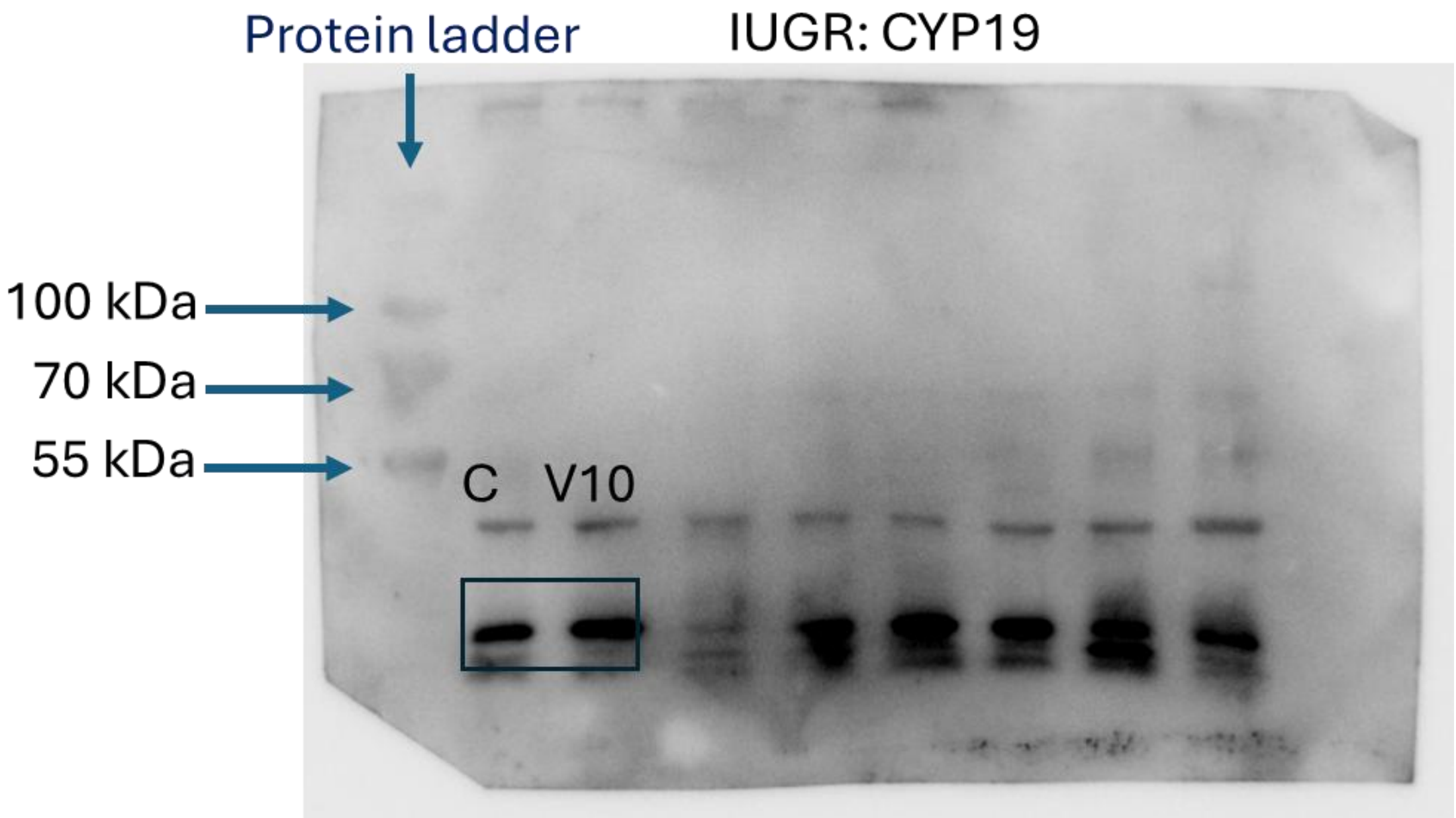

ACTB: 42 kDa

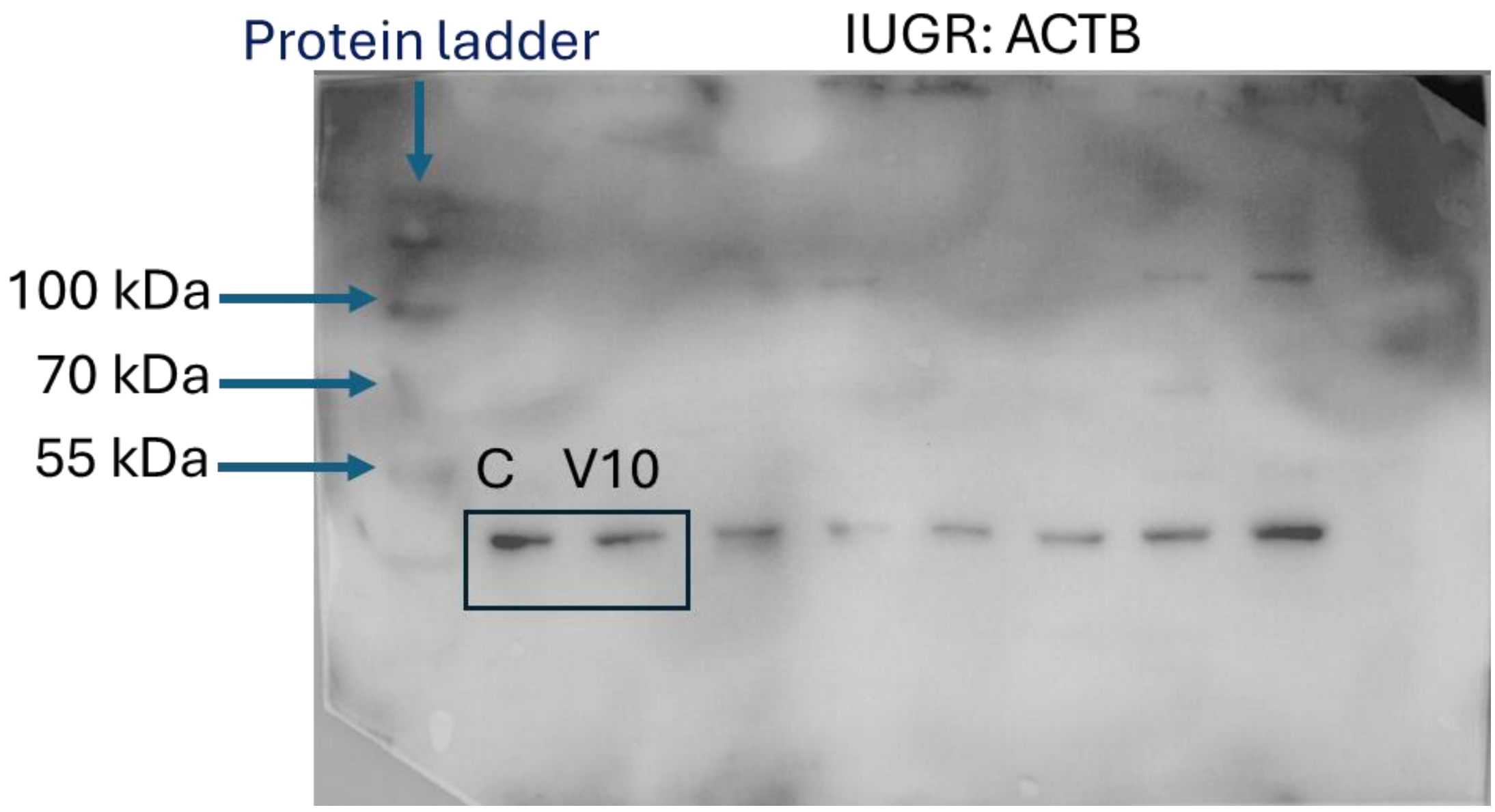

CYP19: 30 kDa

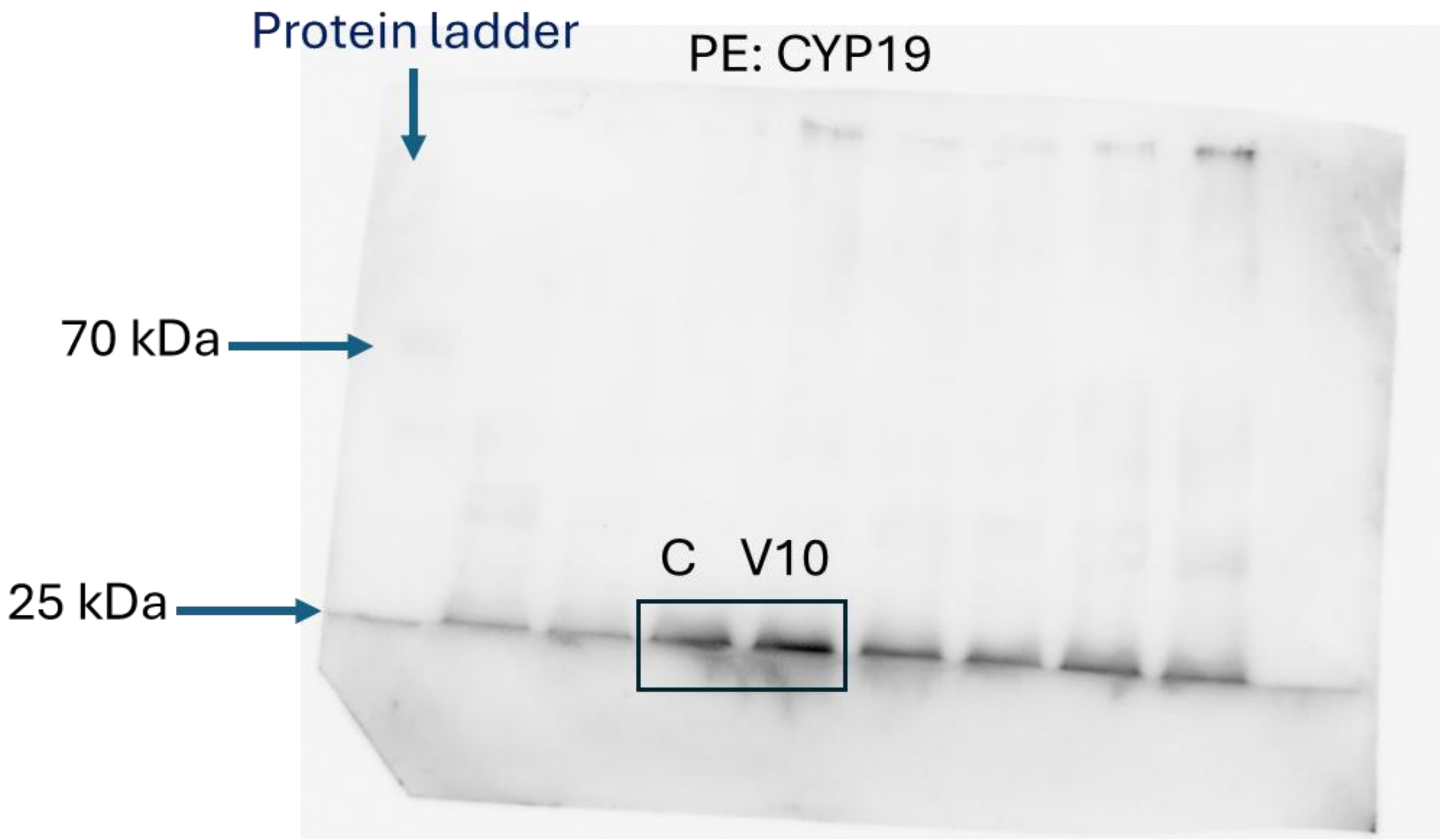

ACTB: 42 kDa

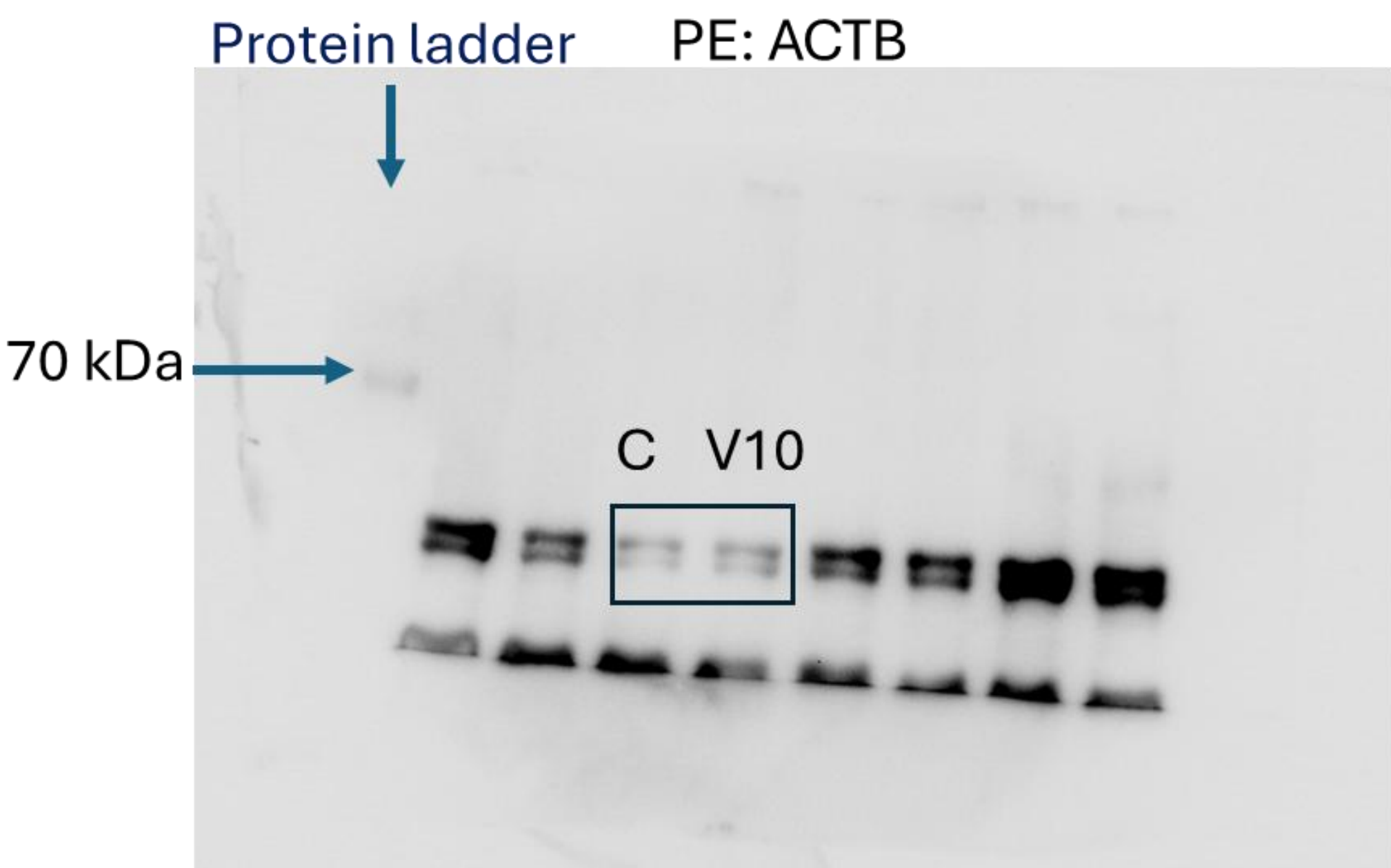

CYP19: 30 kDa

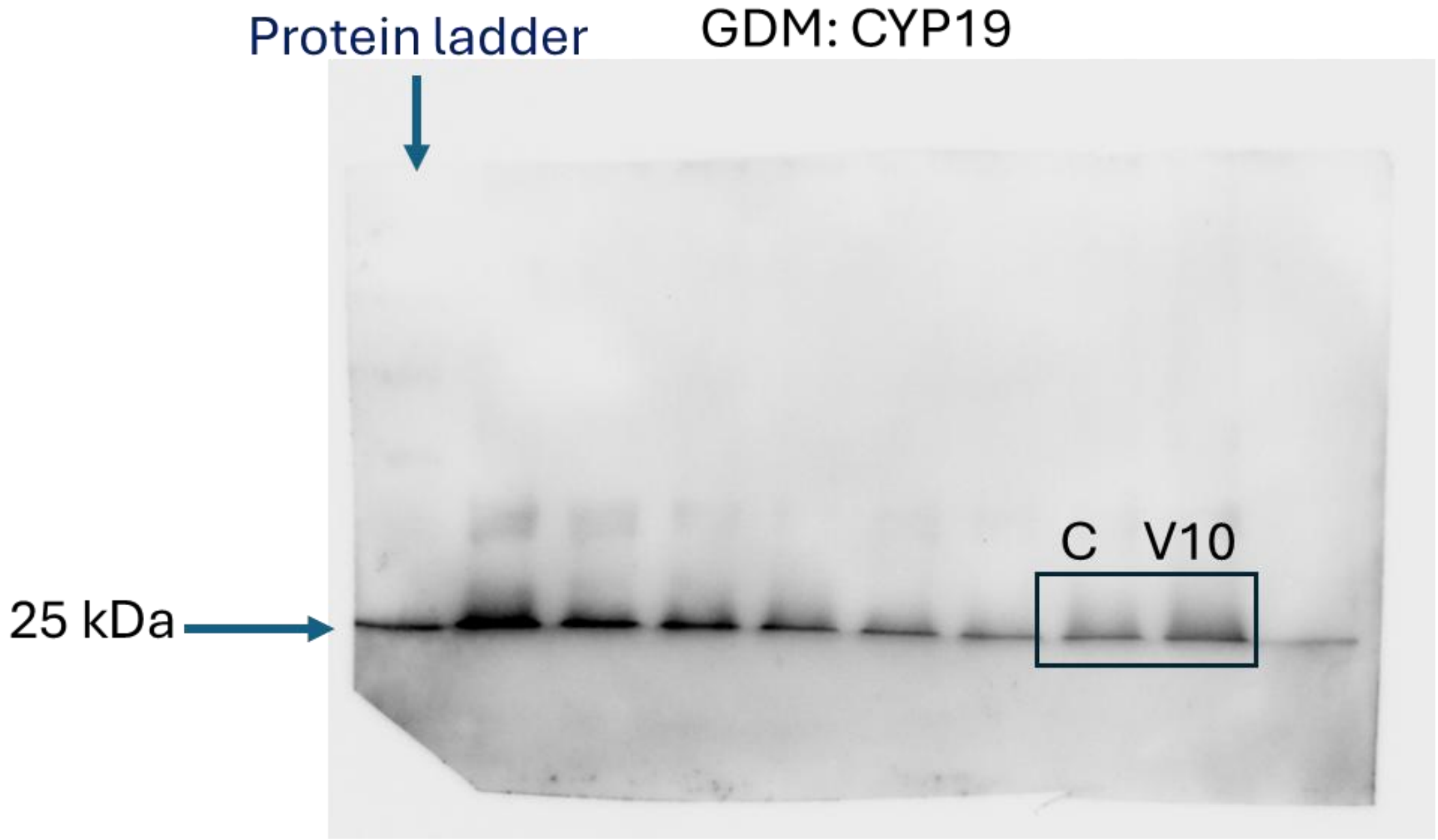

ACTB: 42 kDa

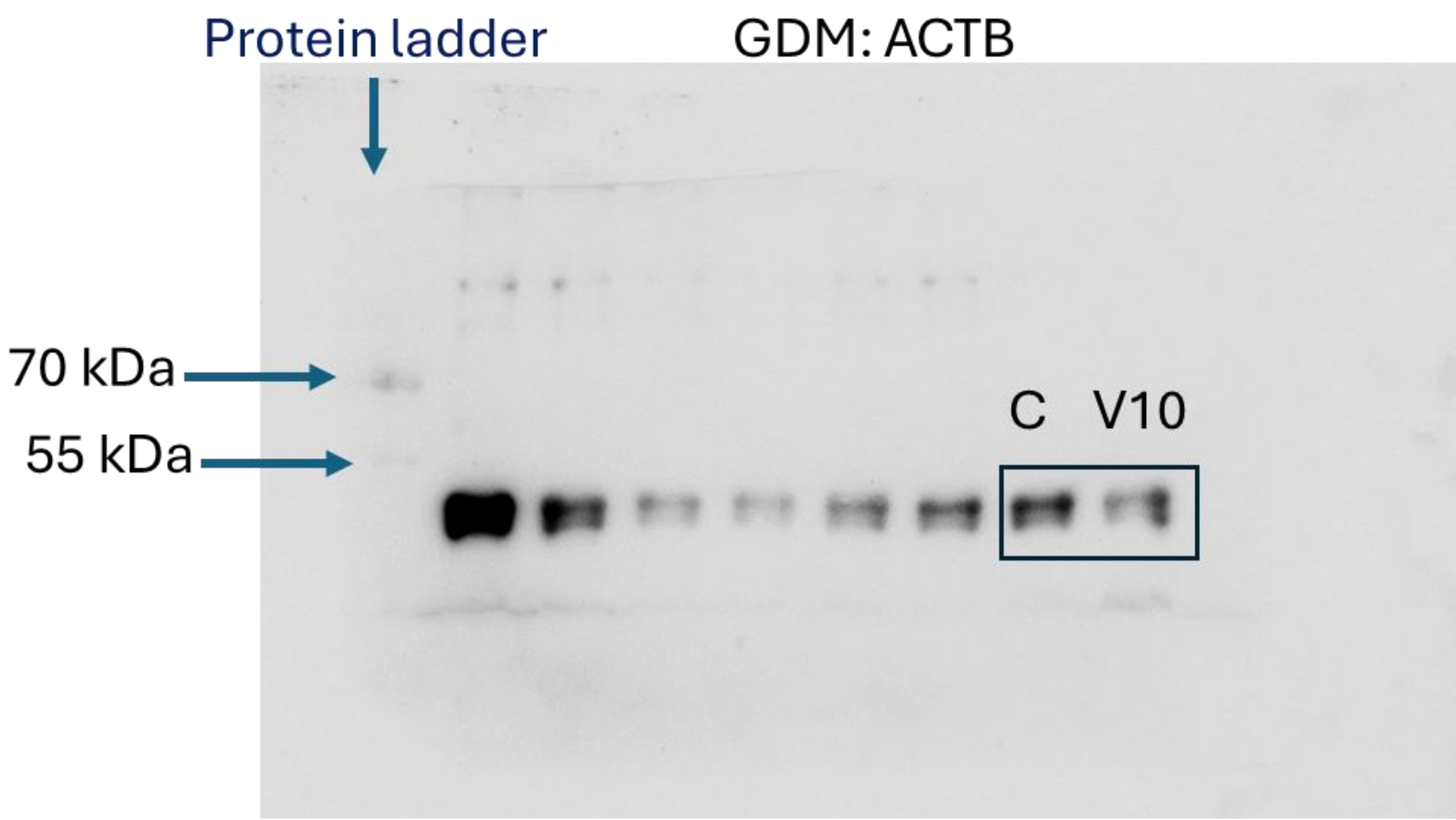

Representative original blots to protein expression of hCG, hPL, GH2 and ACTB in human placenta (Fig. 4a, 4b, 4c).

Fig 4a

hCG: 22 kDa

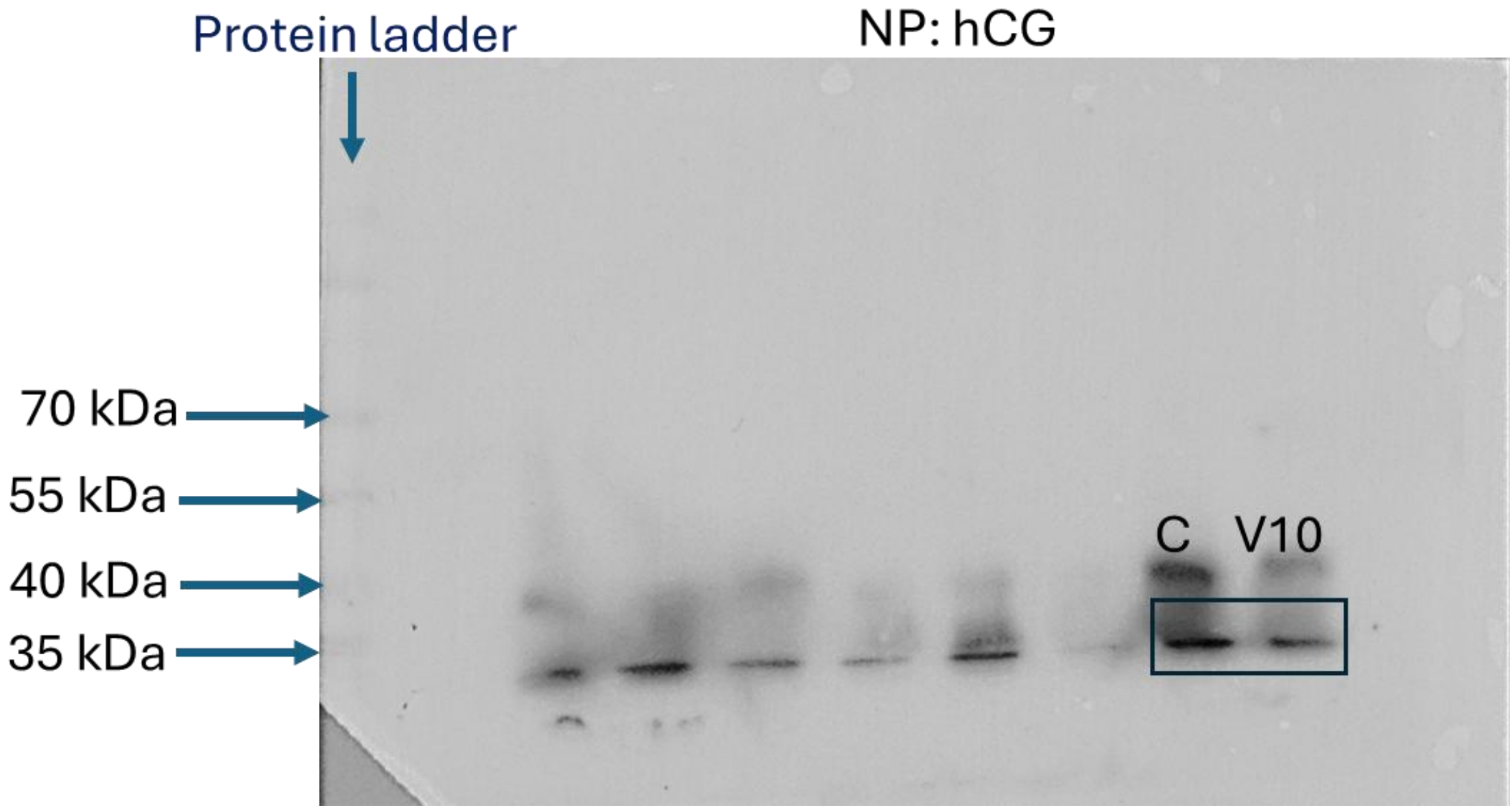

ACTB: 42 kDa

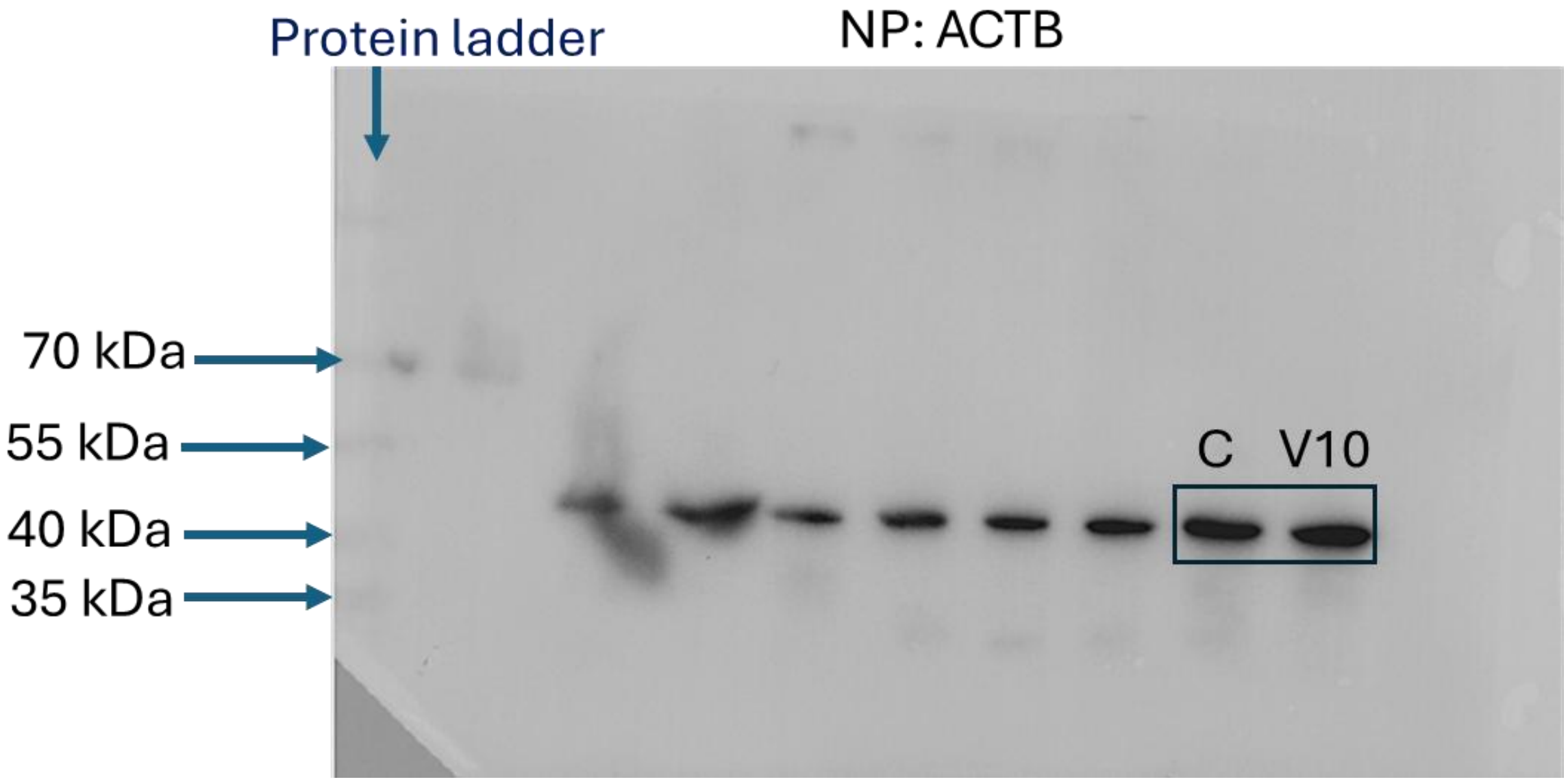

hCG: 22 kDa

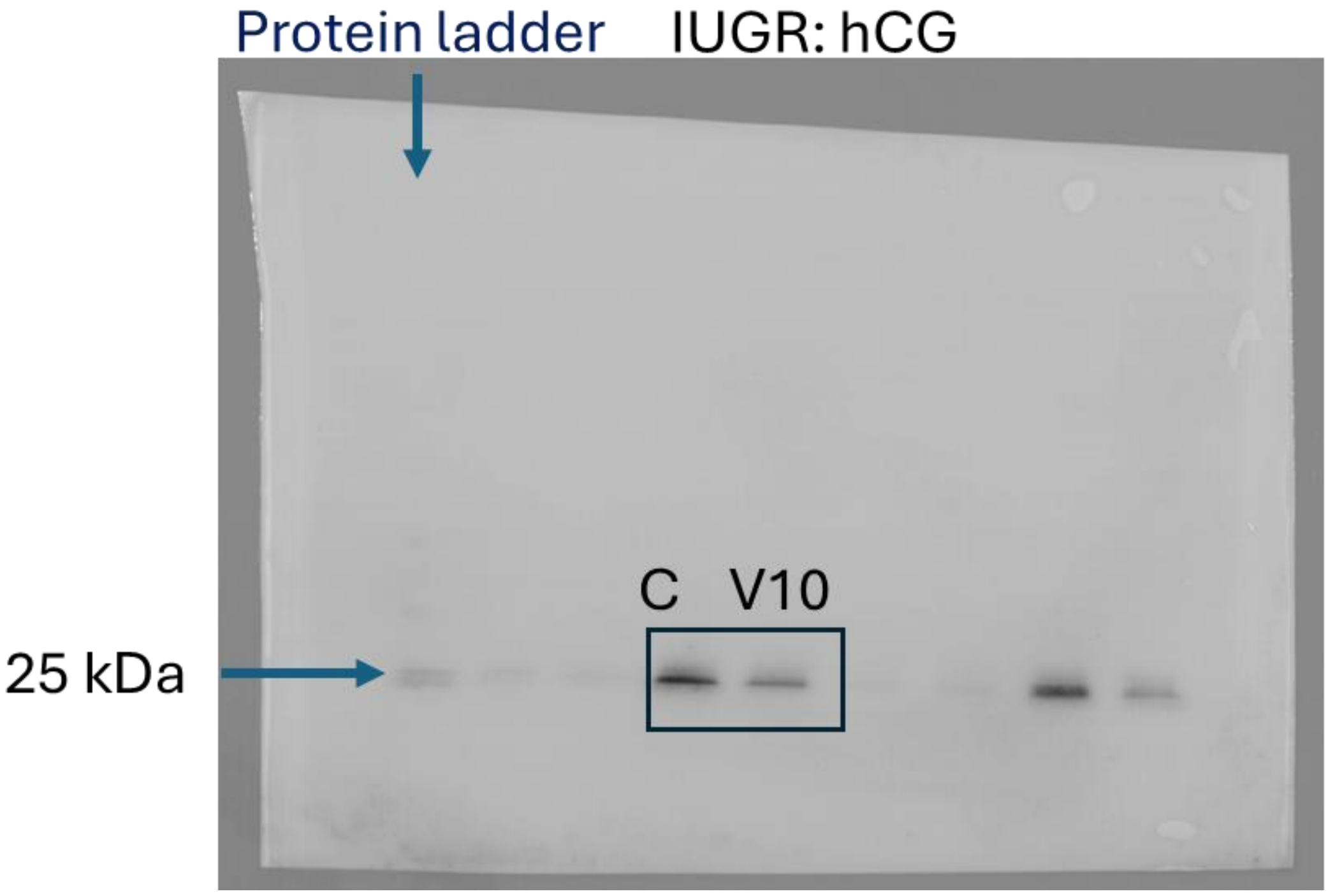

ACTB: 42 kDa

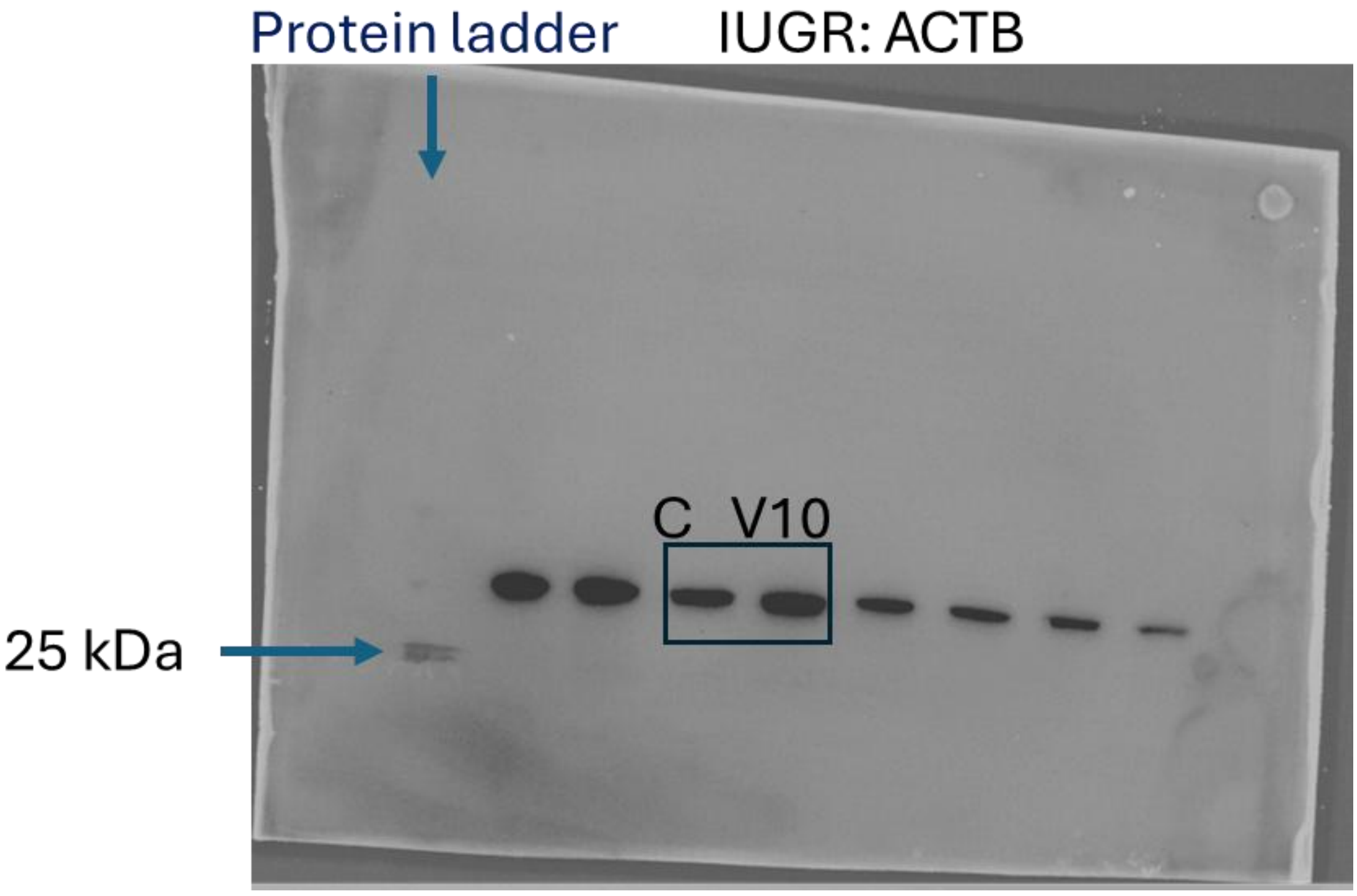

hCG: 22 kDa

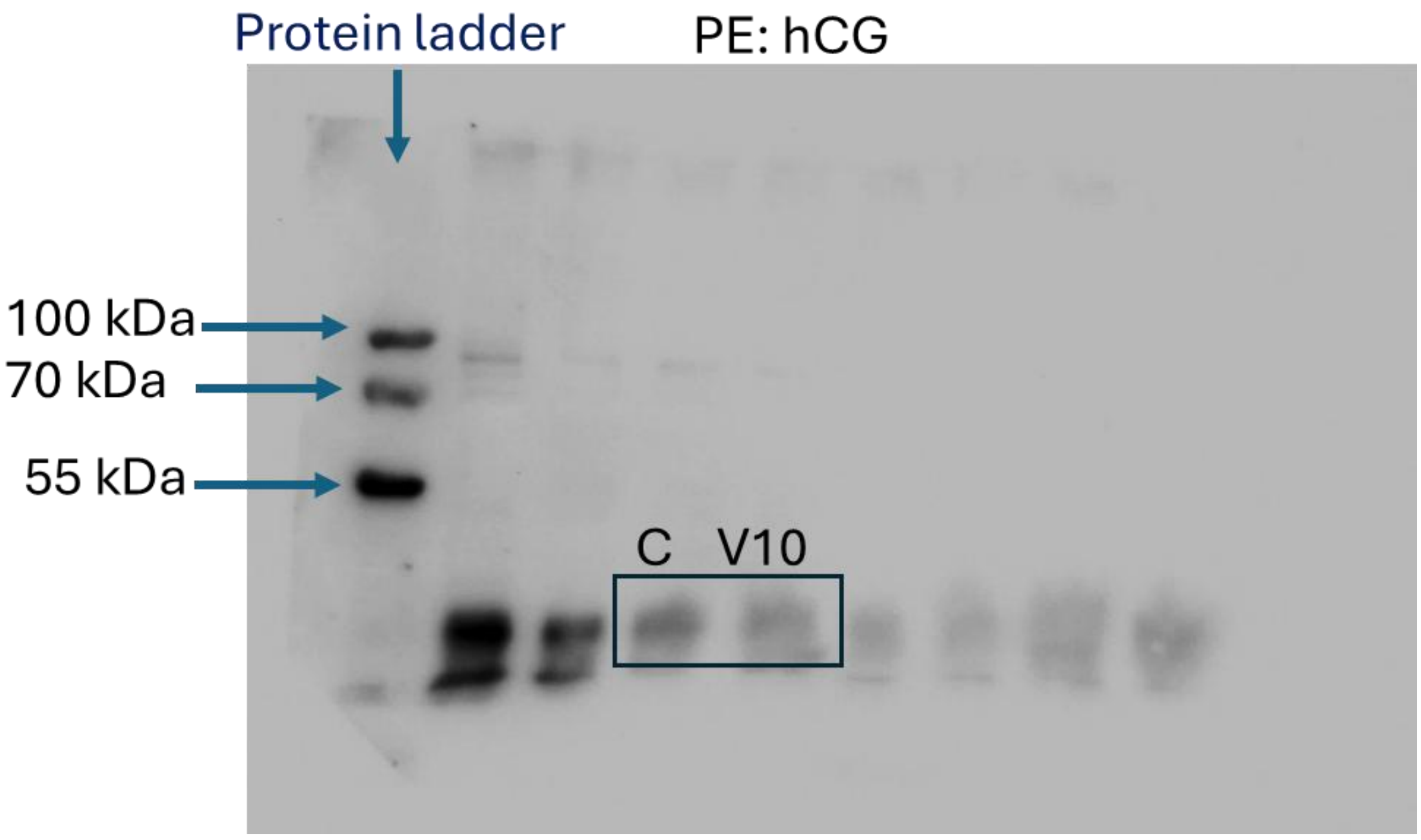

ACTB: 42 kDa

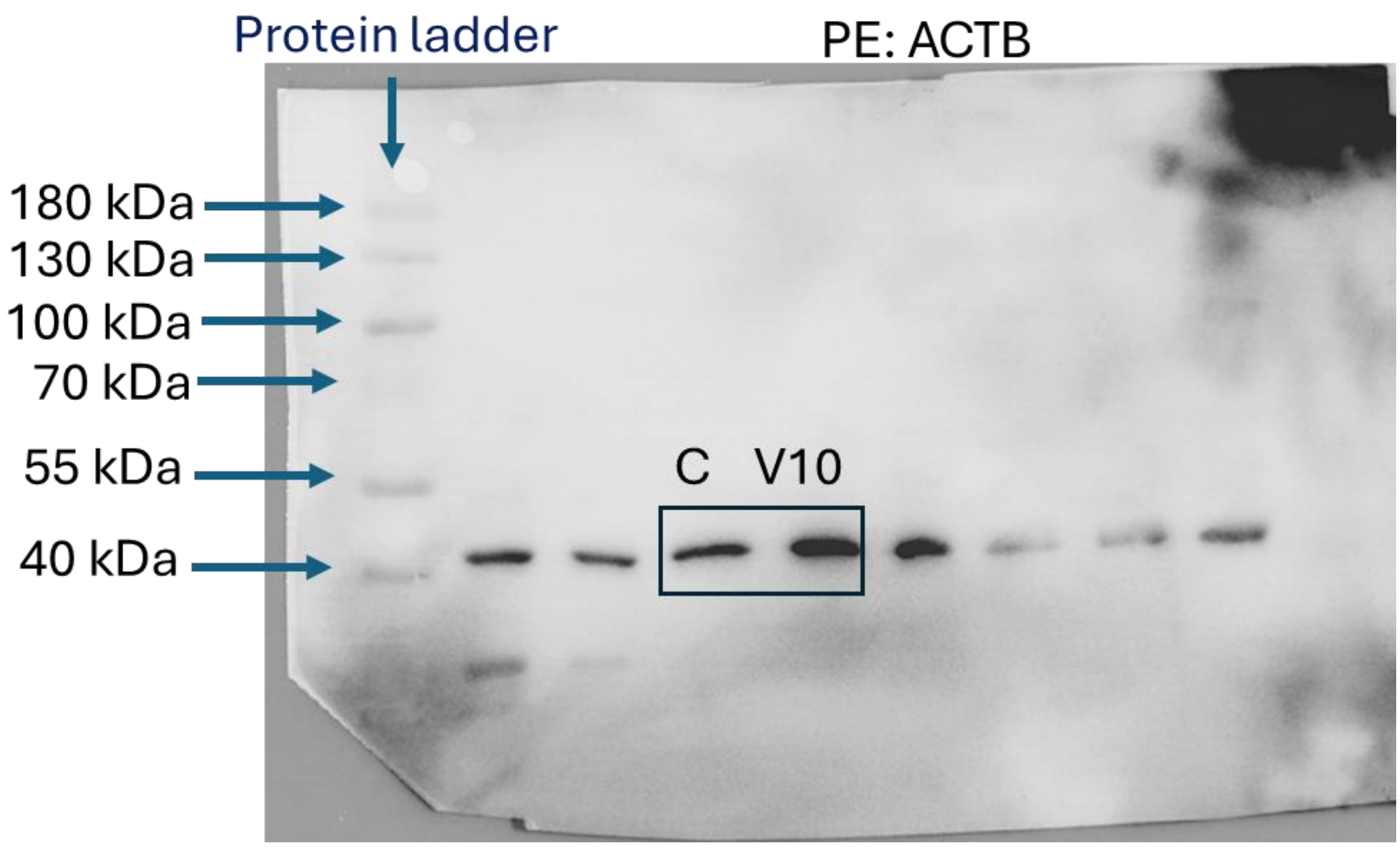

hCG: 22 kDa

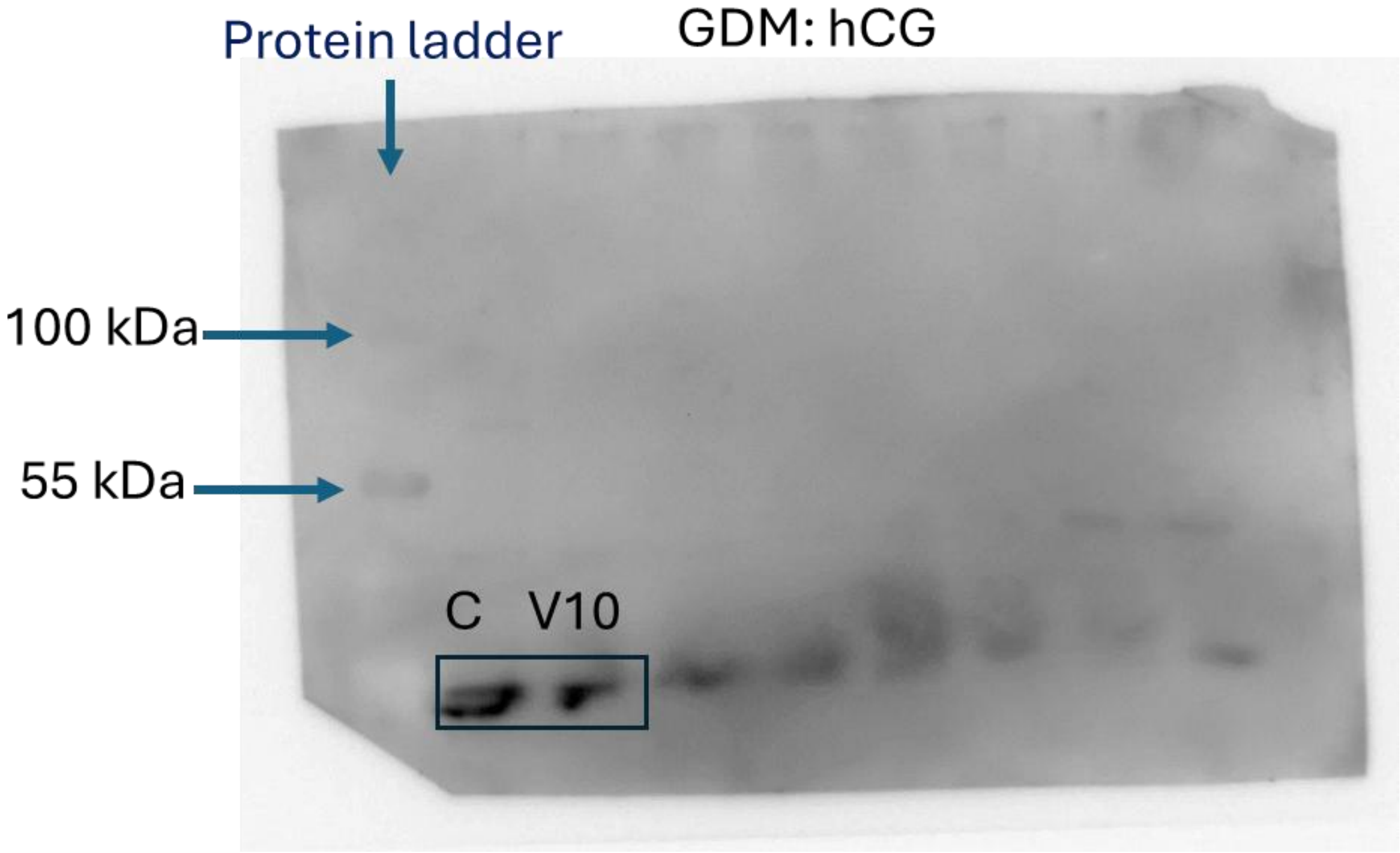

ACTB: 42 kDa

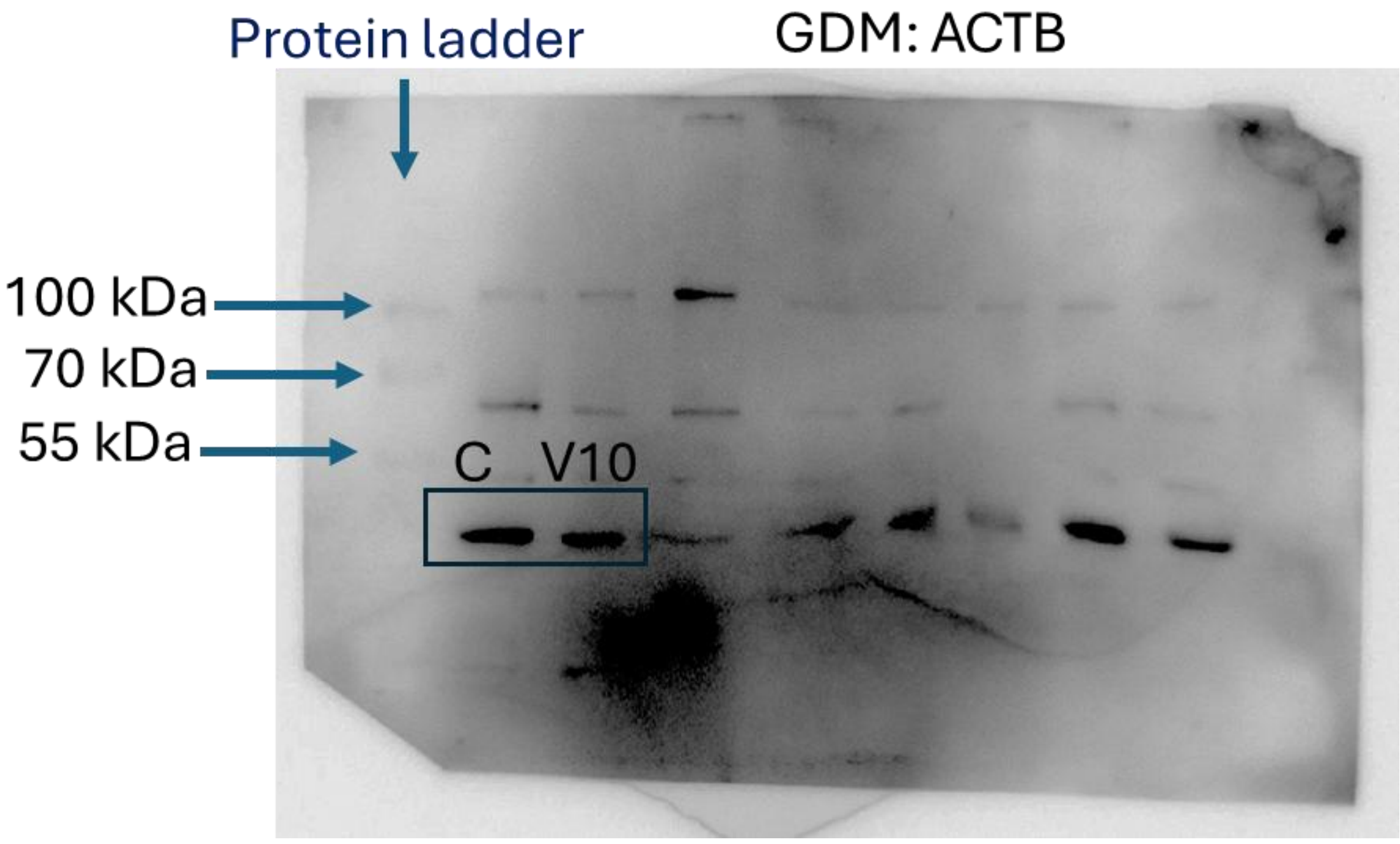

Fig 4c

hPL: 22 kDa

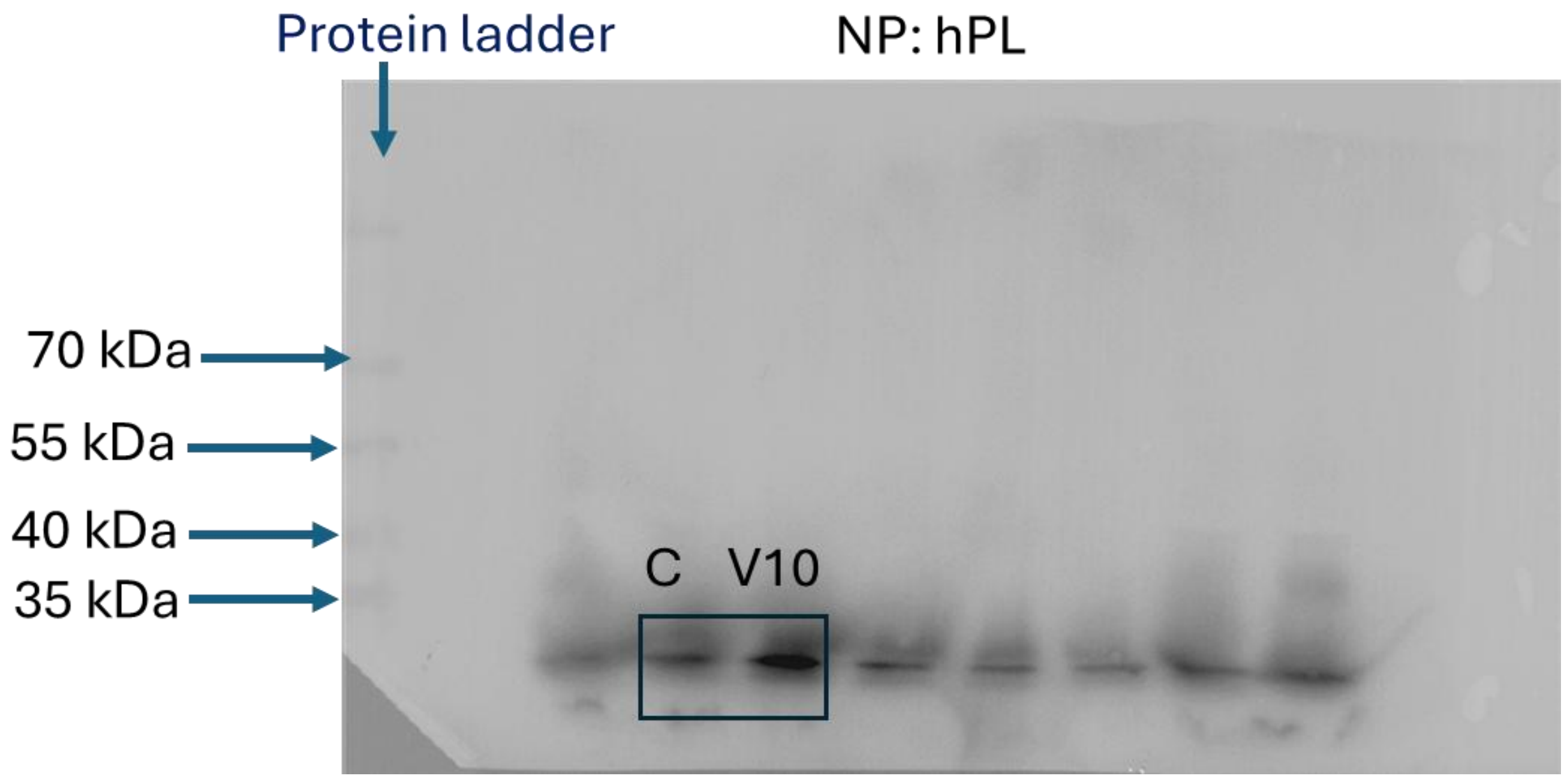

ACTB: 42 kDa

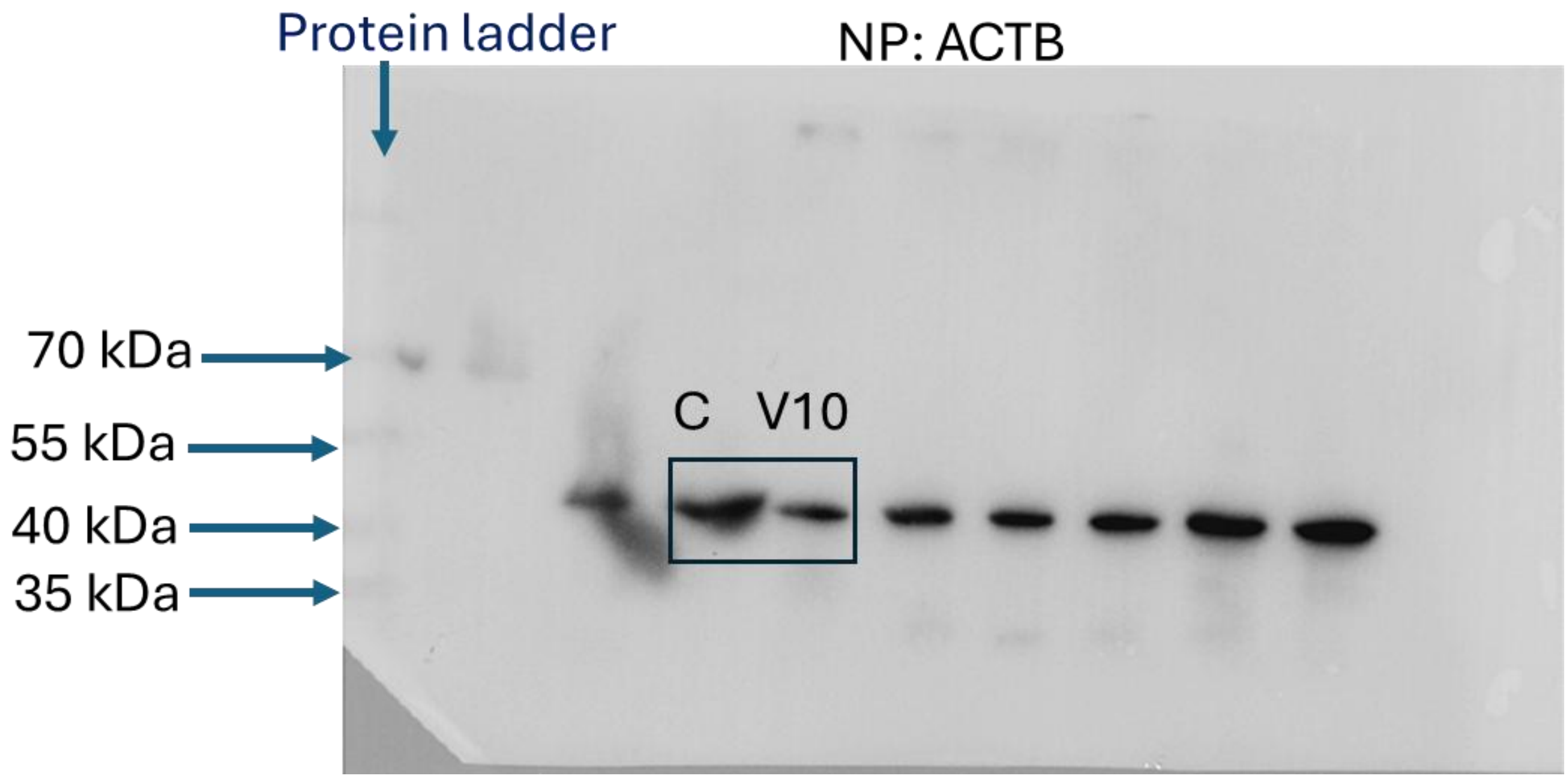

hPL: 22 kDa

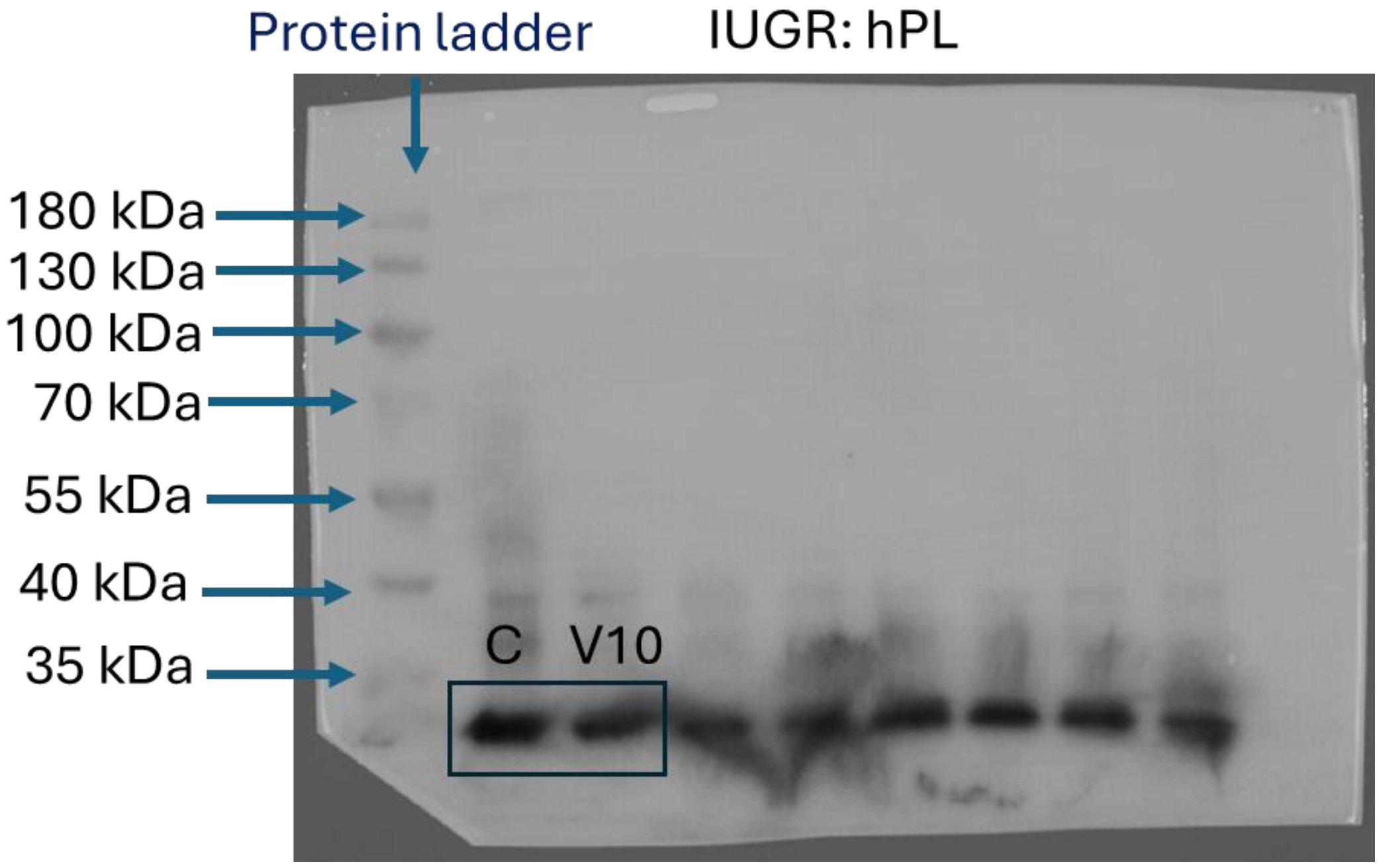

ACTB: 42 kDa

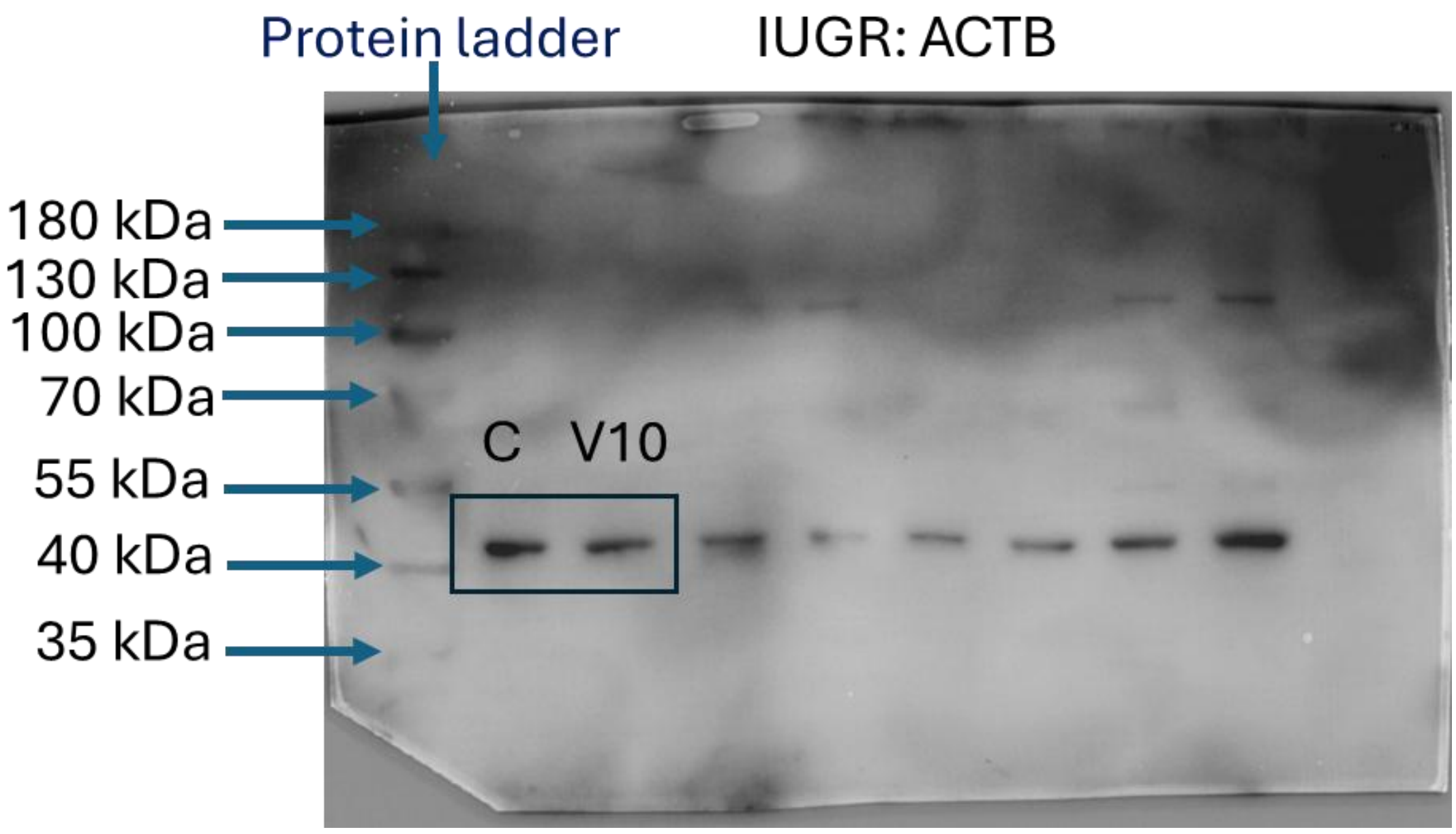

hPL: 22 kDa

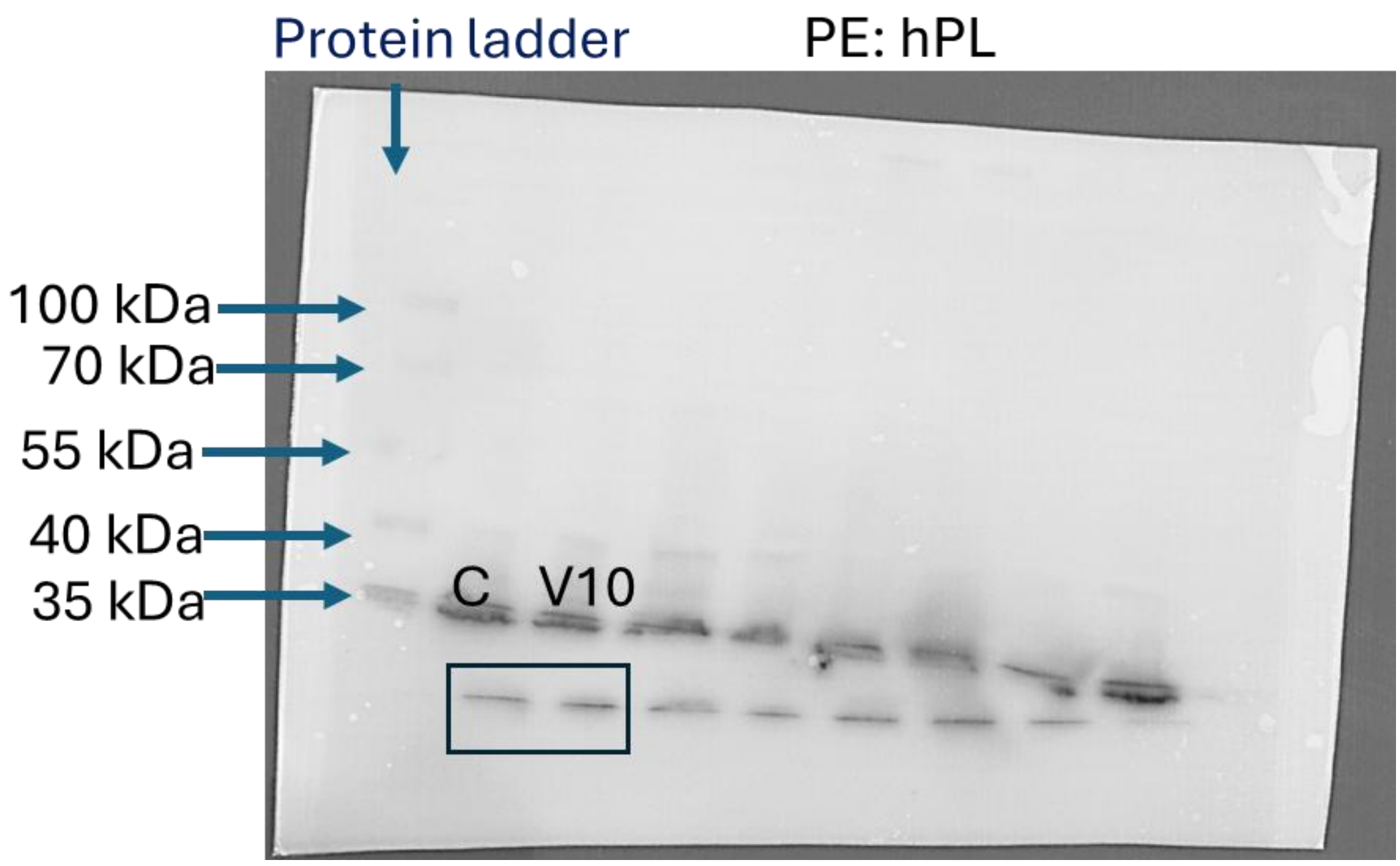

ACTB: 42 kDa

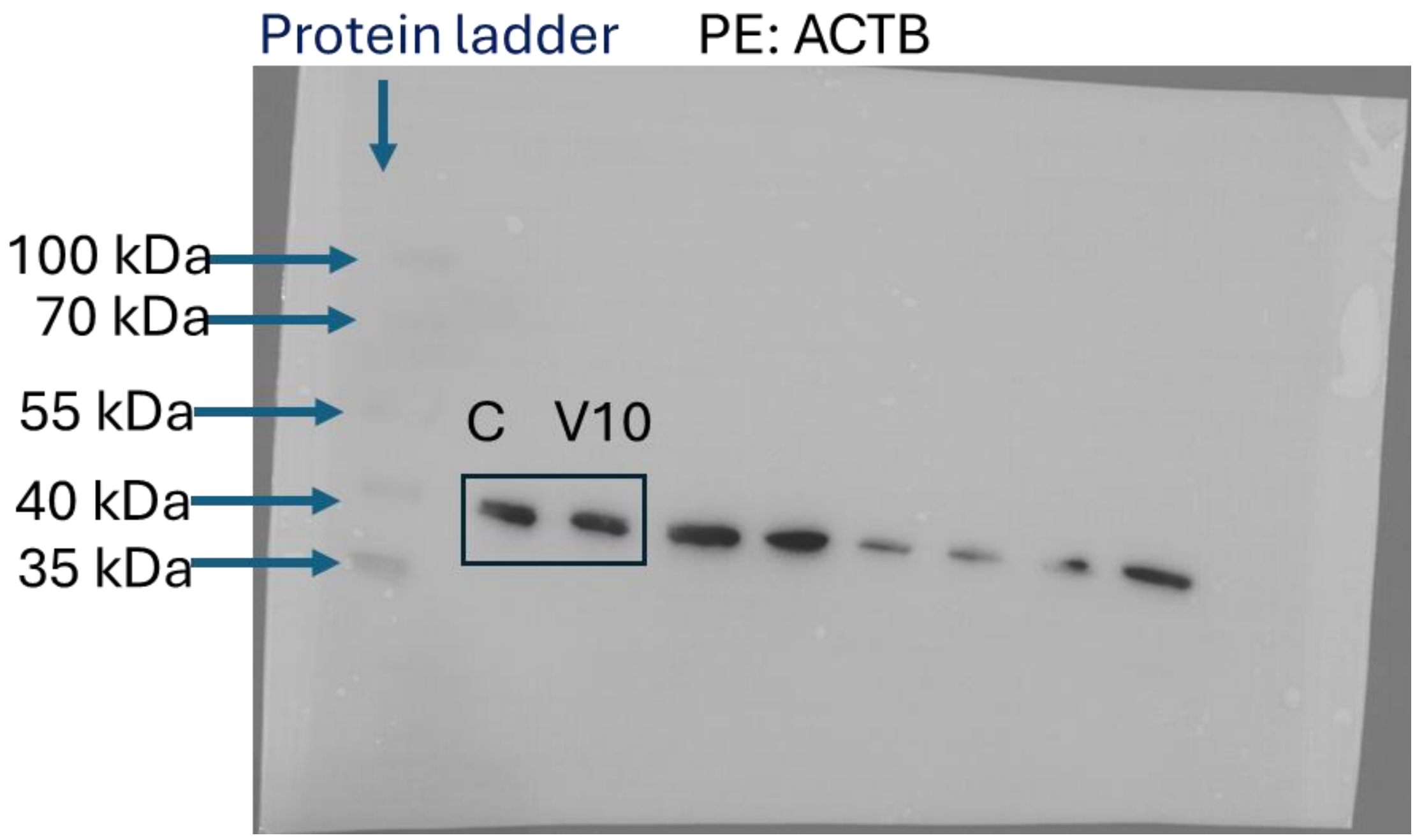

hPL: 22 kDa

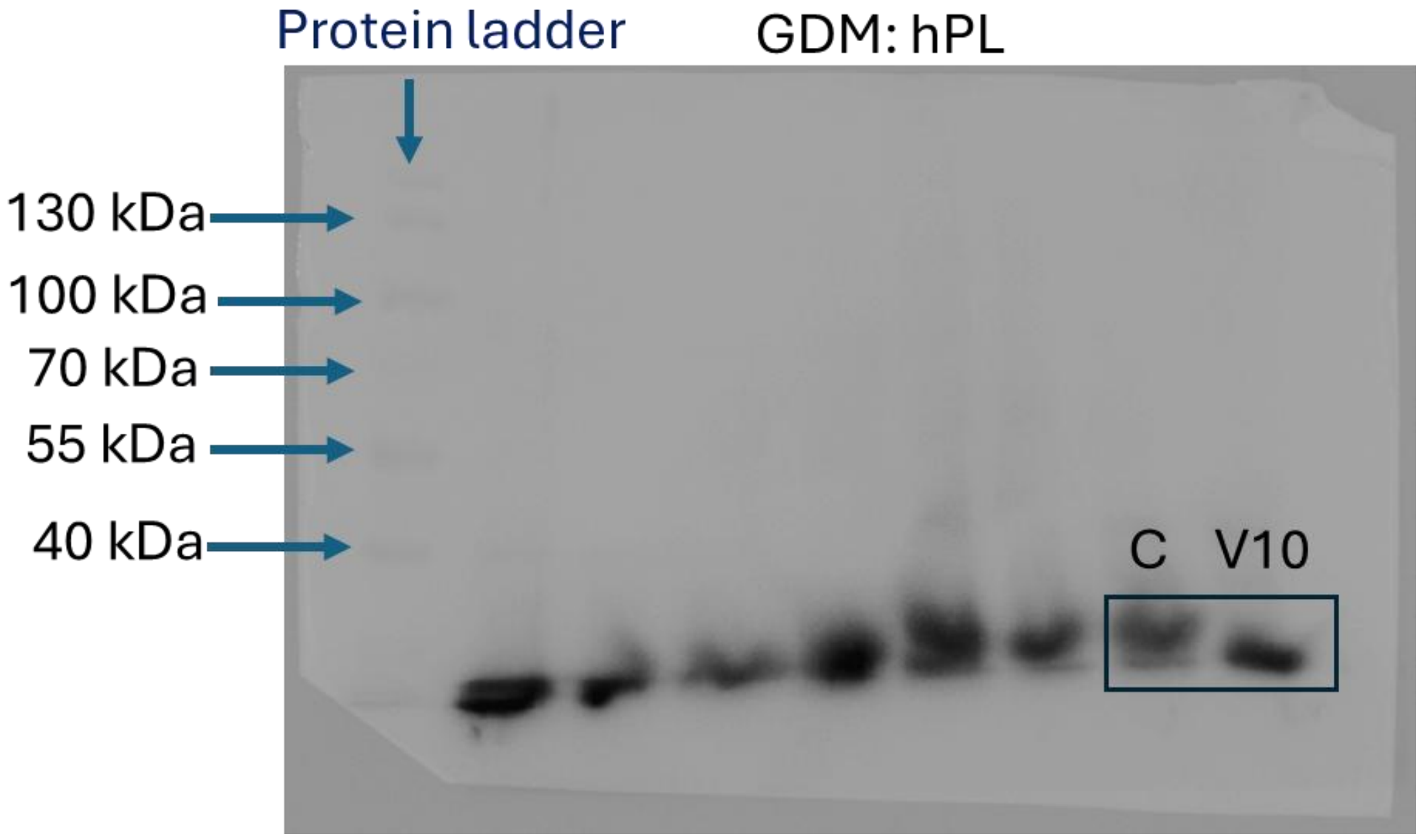

ACTB: 42 kDa

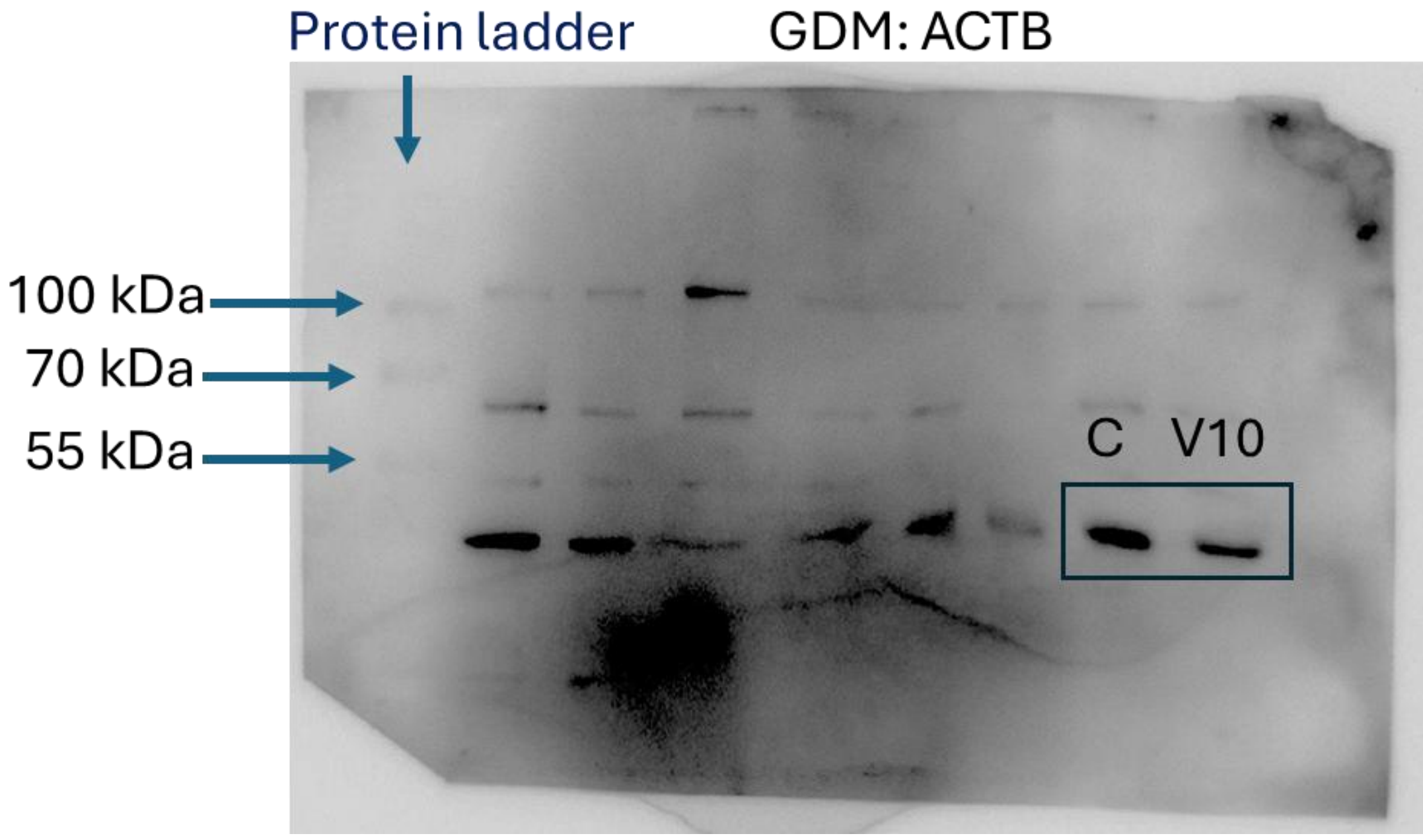

Fig 4e

GH2: 25 kDa

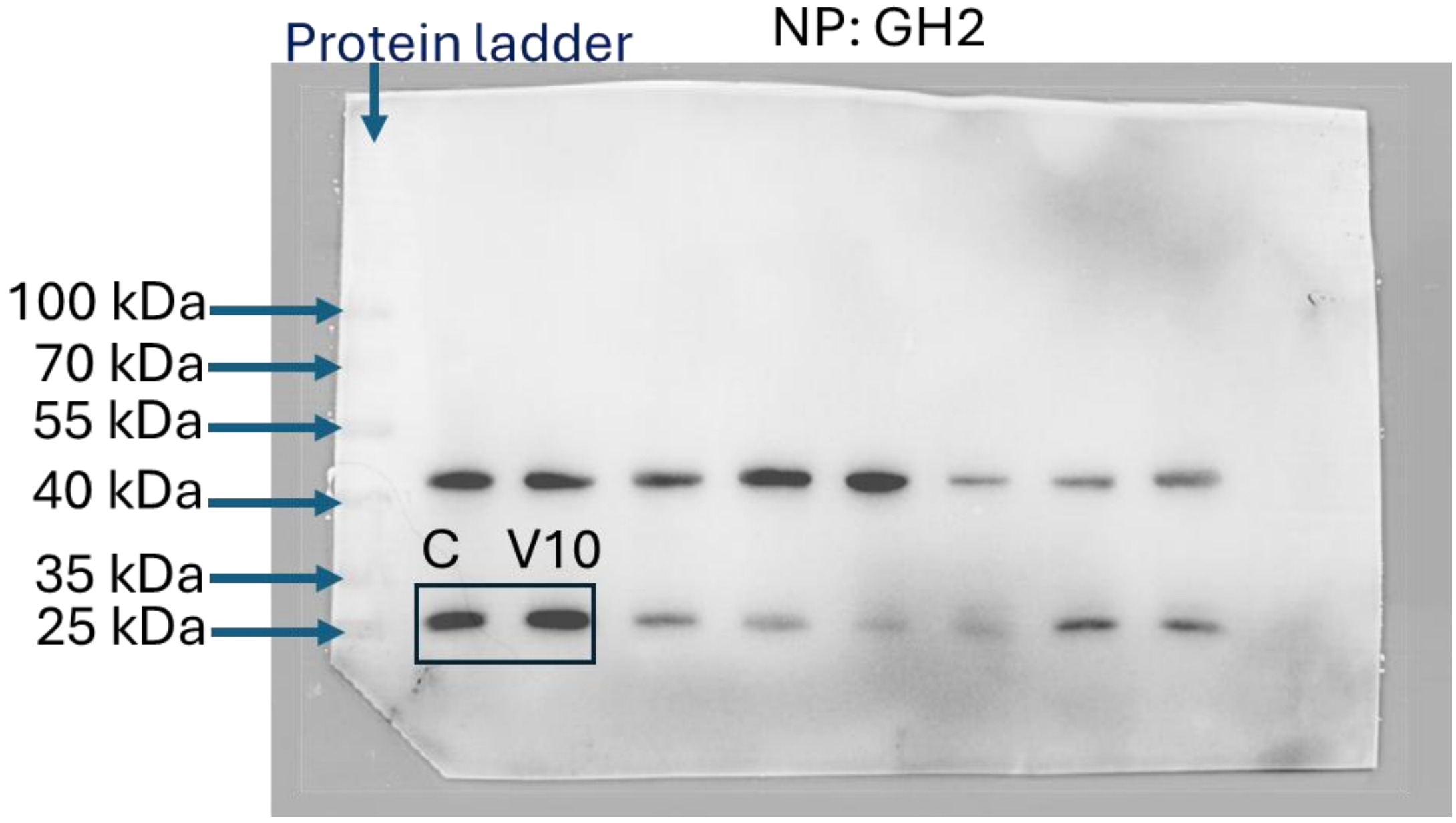

ACTB: 42 kDa

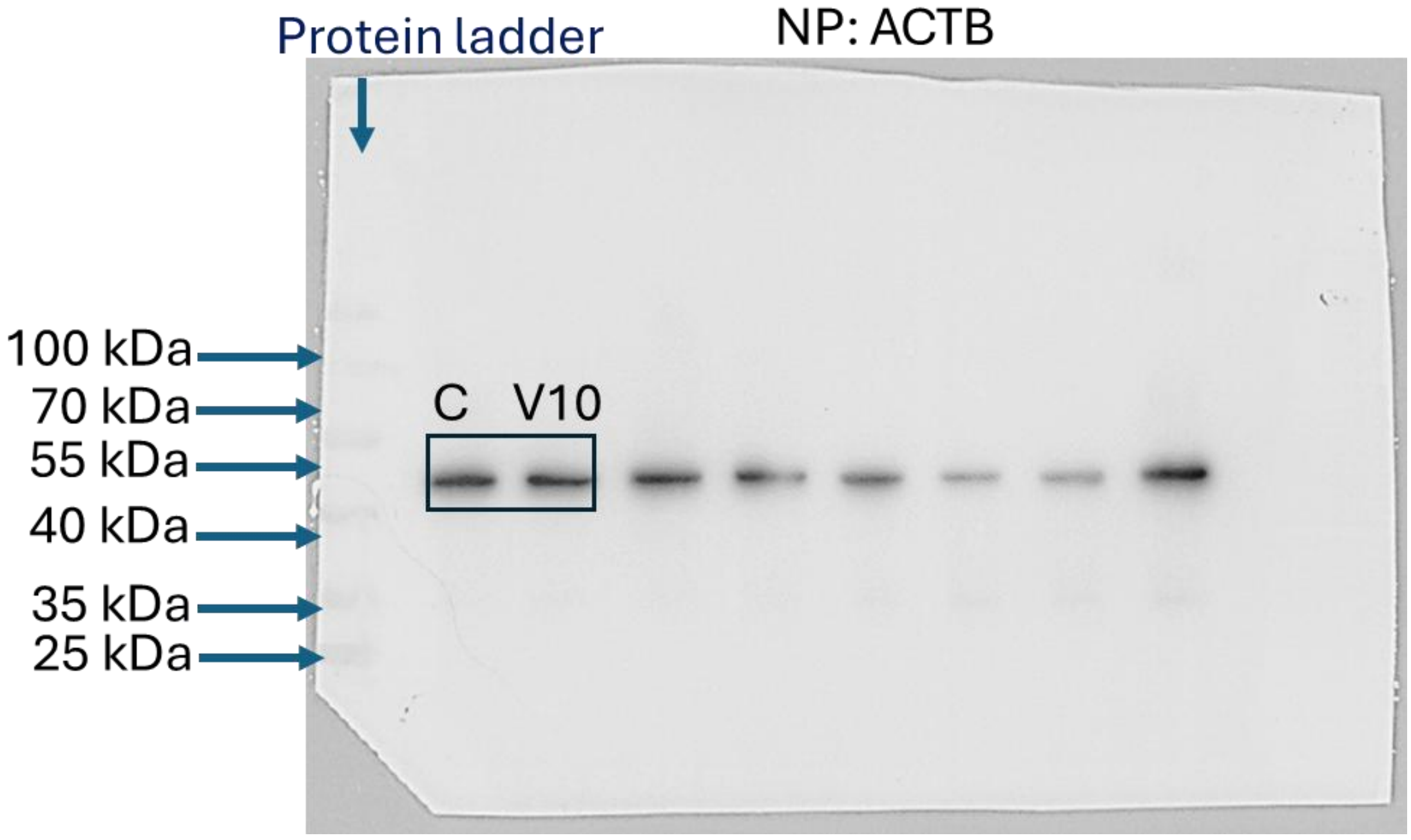

GH2: 25 kDa

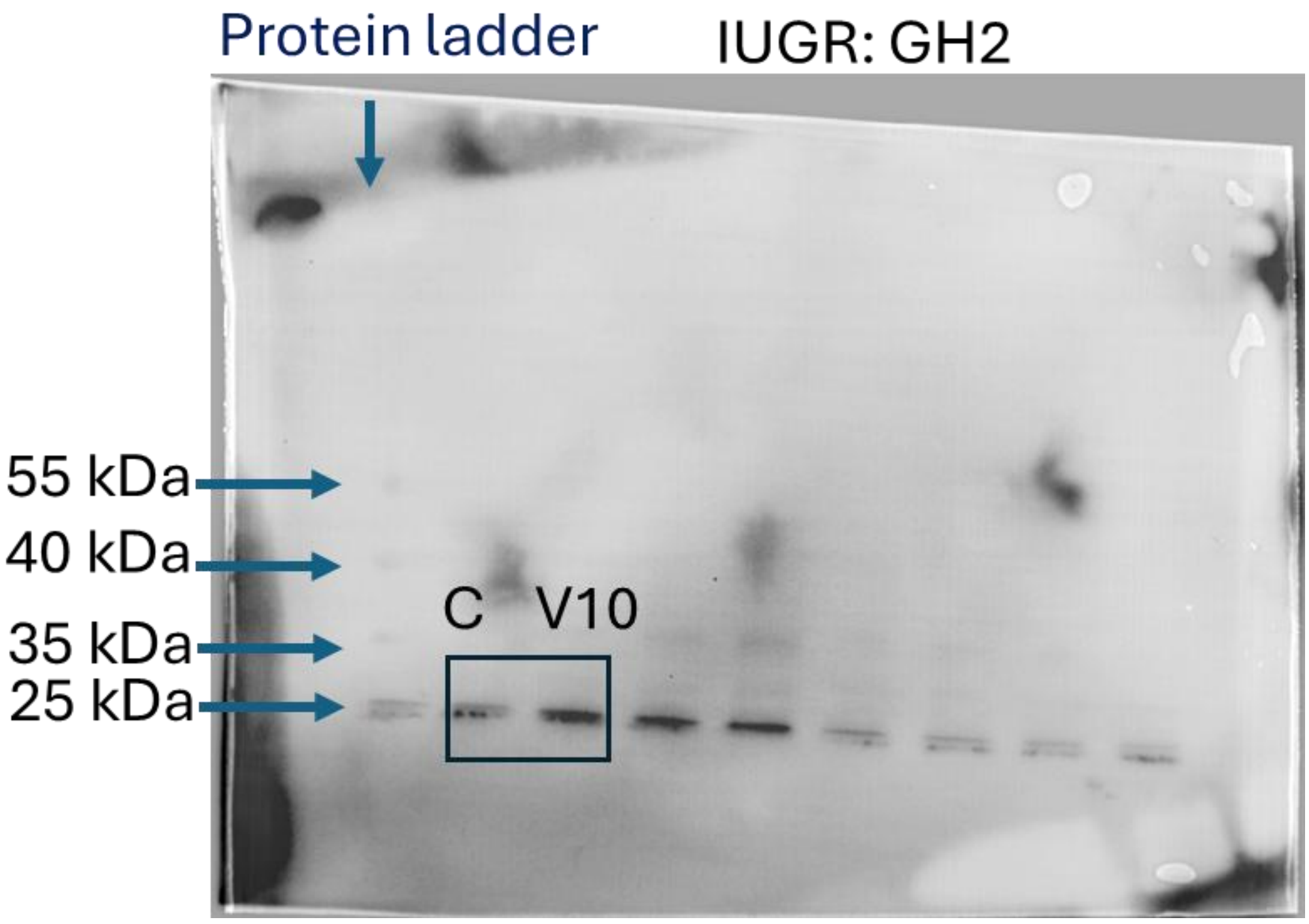

ACTB: 42 kDa

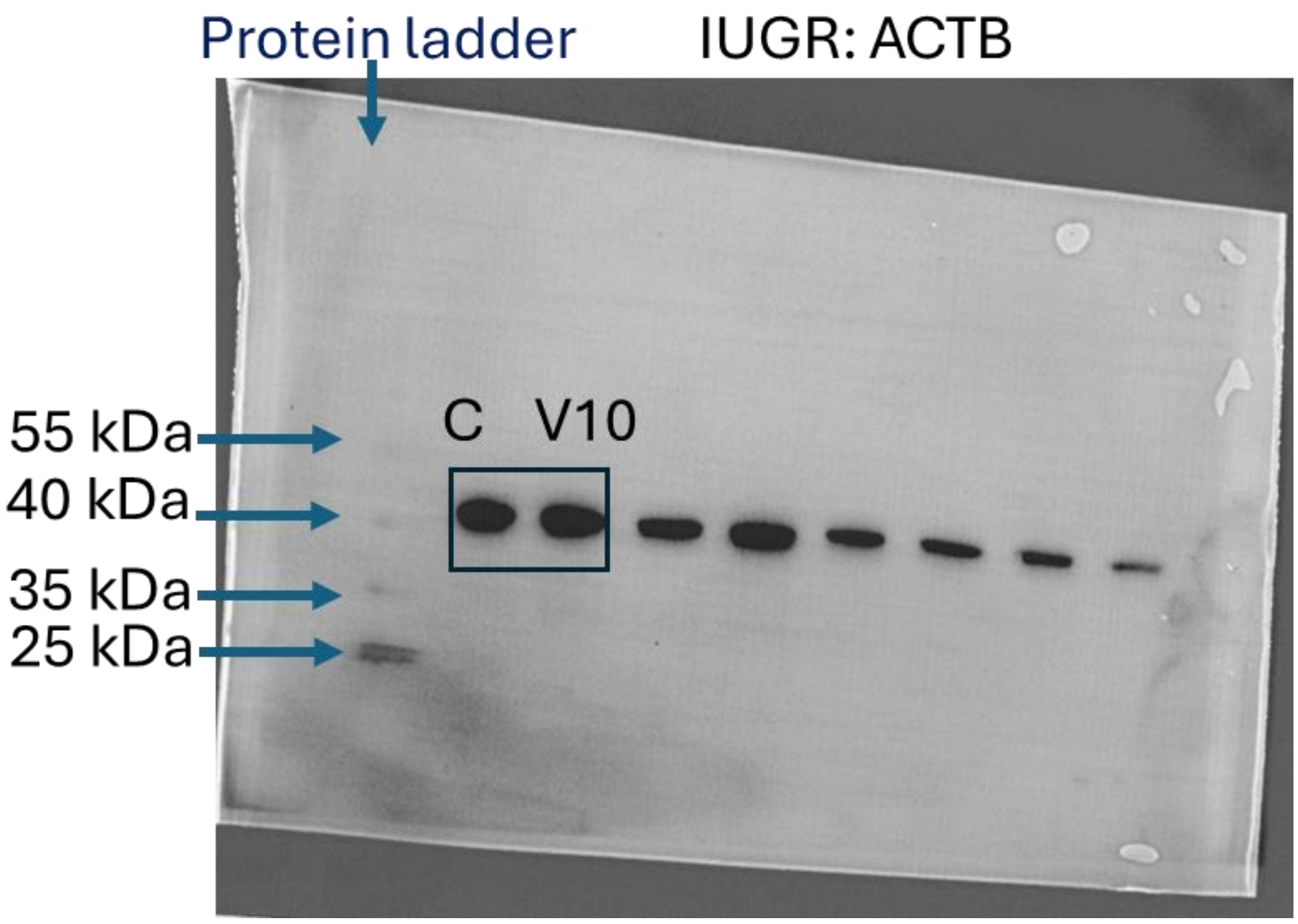

GH2: 25 kDa

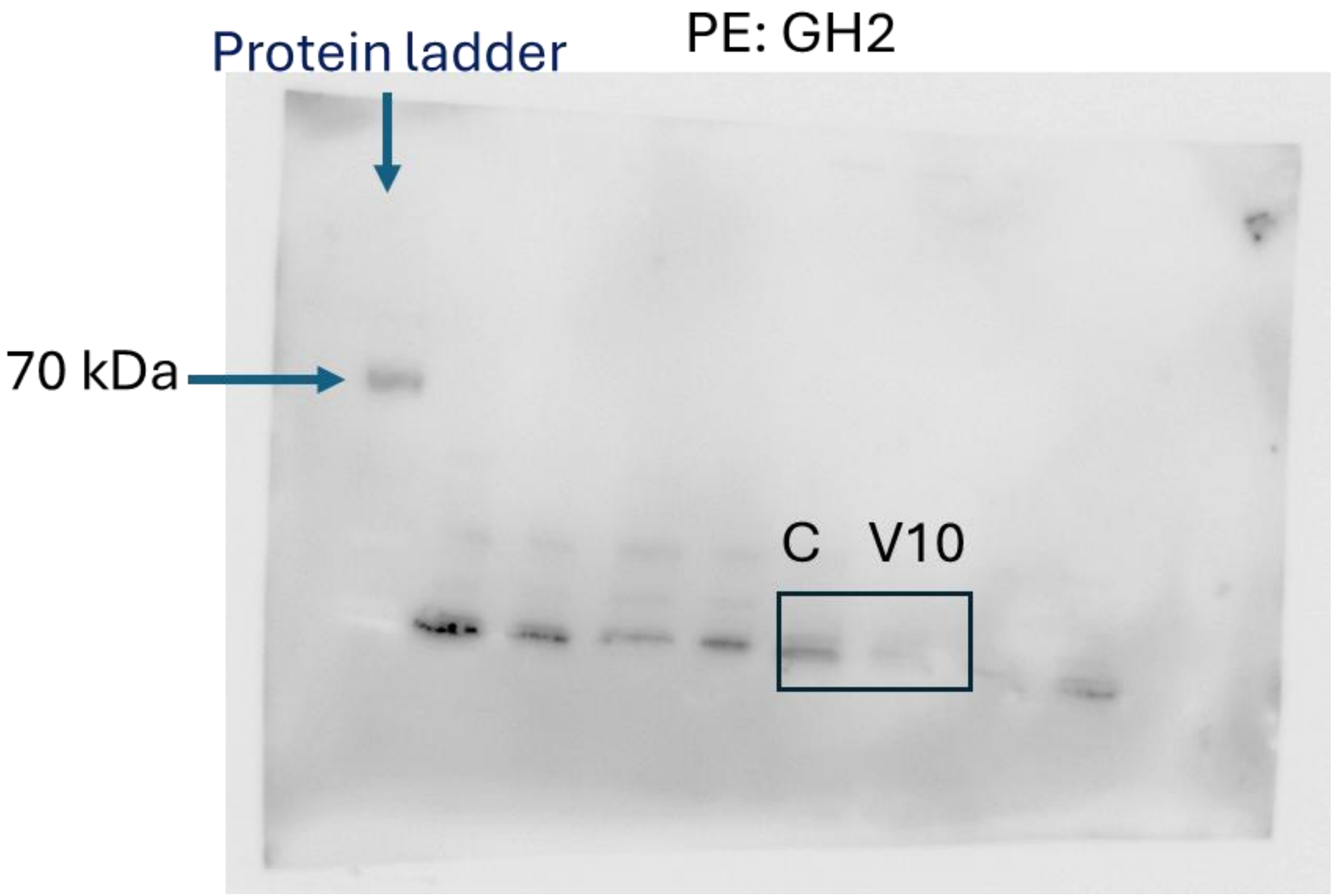

ACTB: 42 kDa

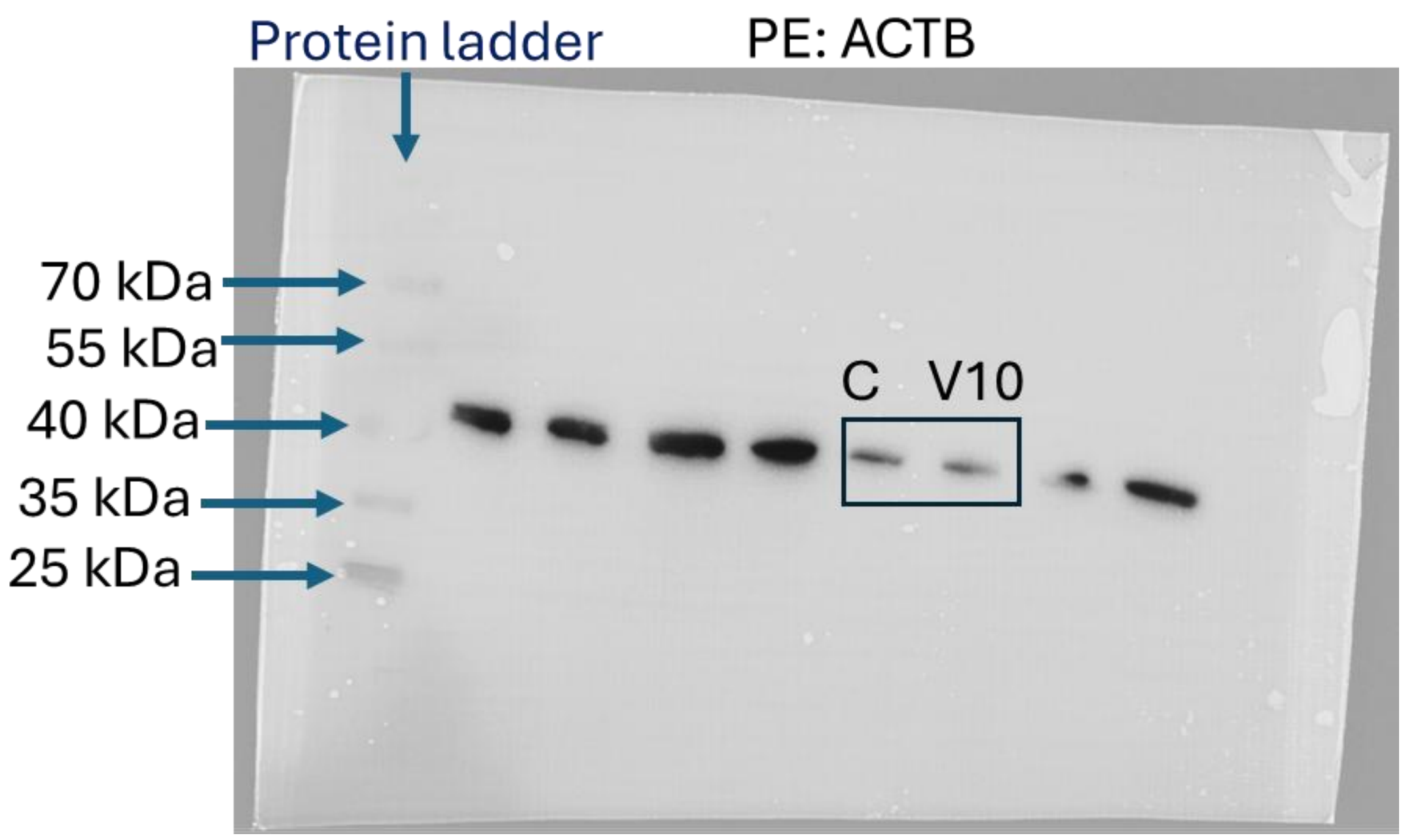

GH2: 25 kDa

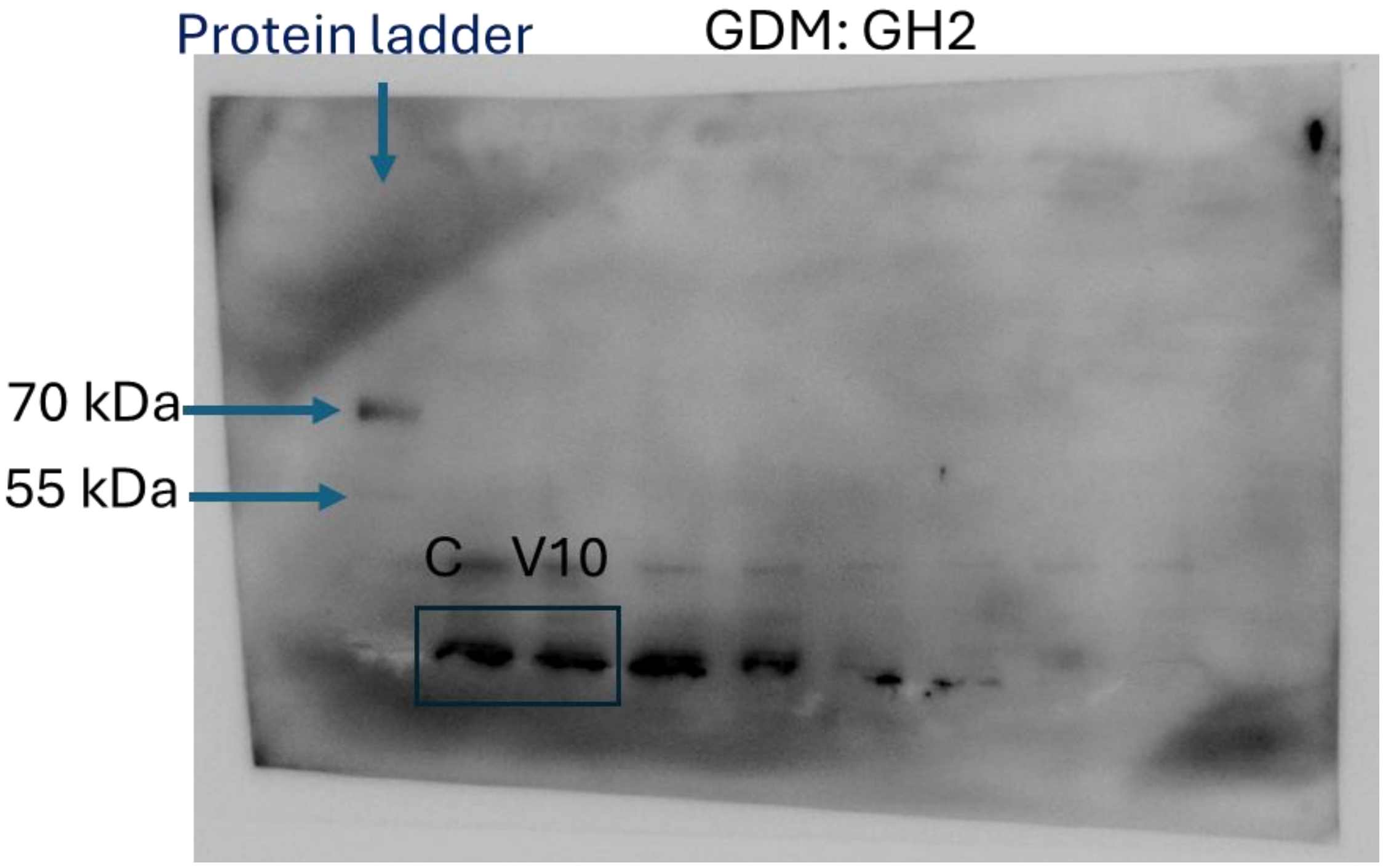

ACTB: 42 kDa

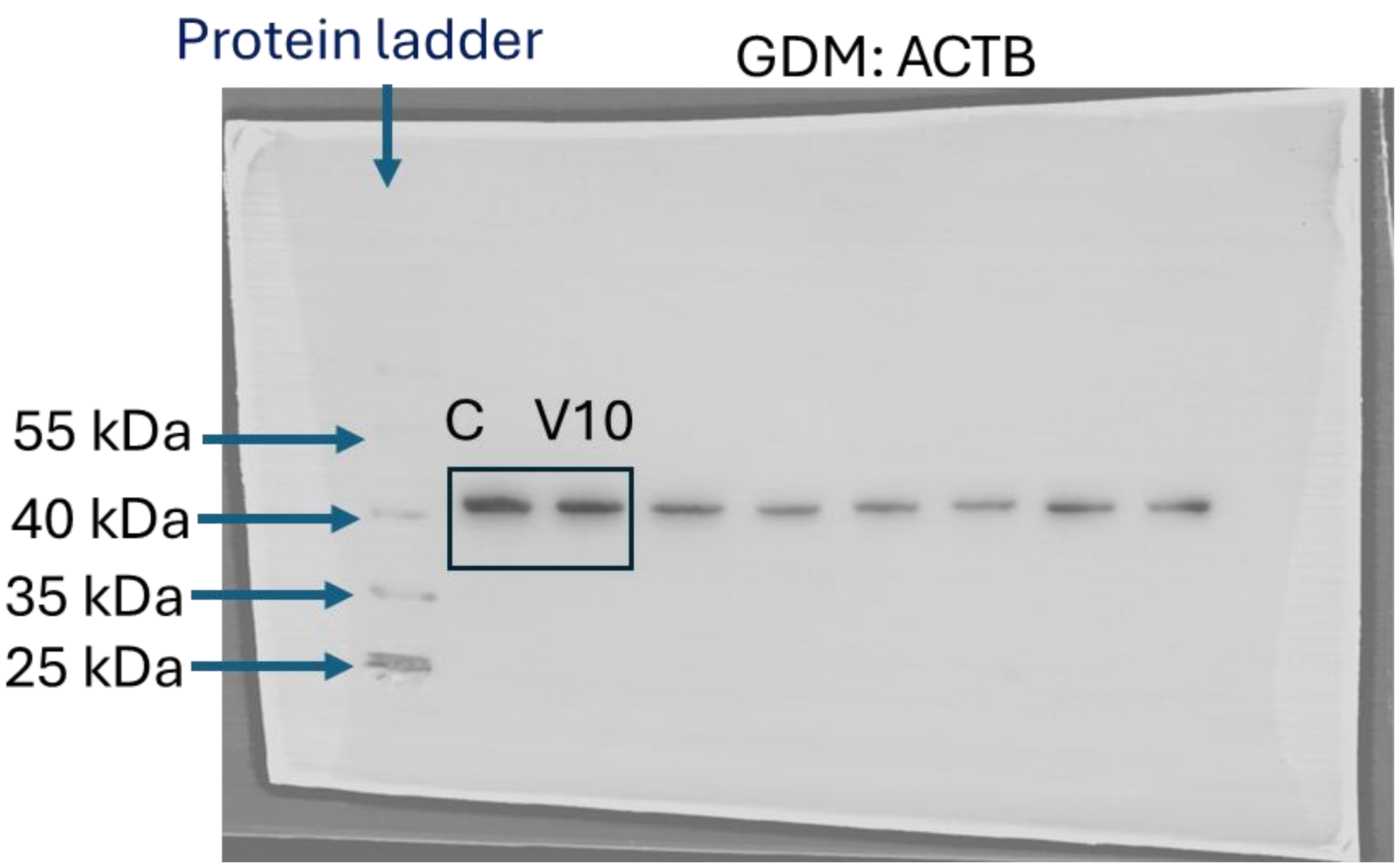

Representative original blots to protein expression of pPKA and tPKA in BeWo cells (Fig. 5a).

Fig. 5a

pPKA: 42 kDa

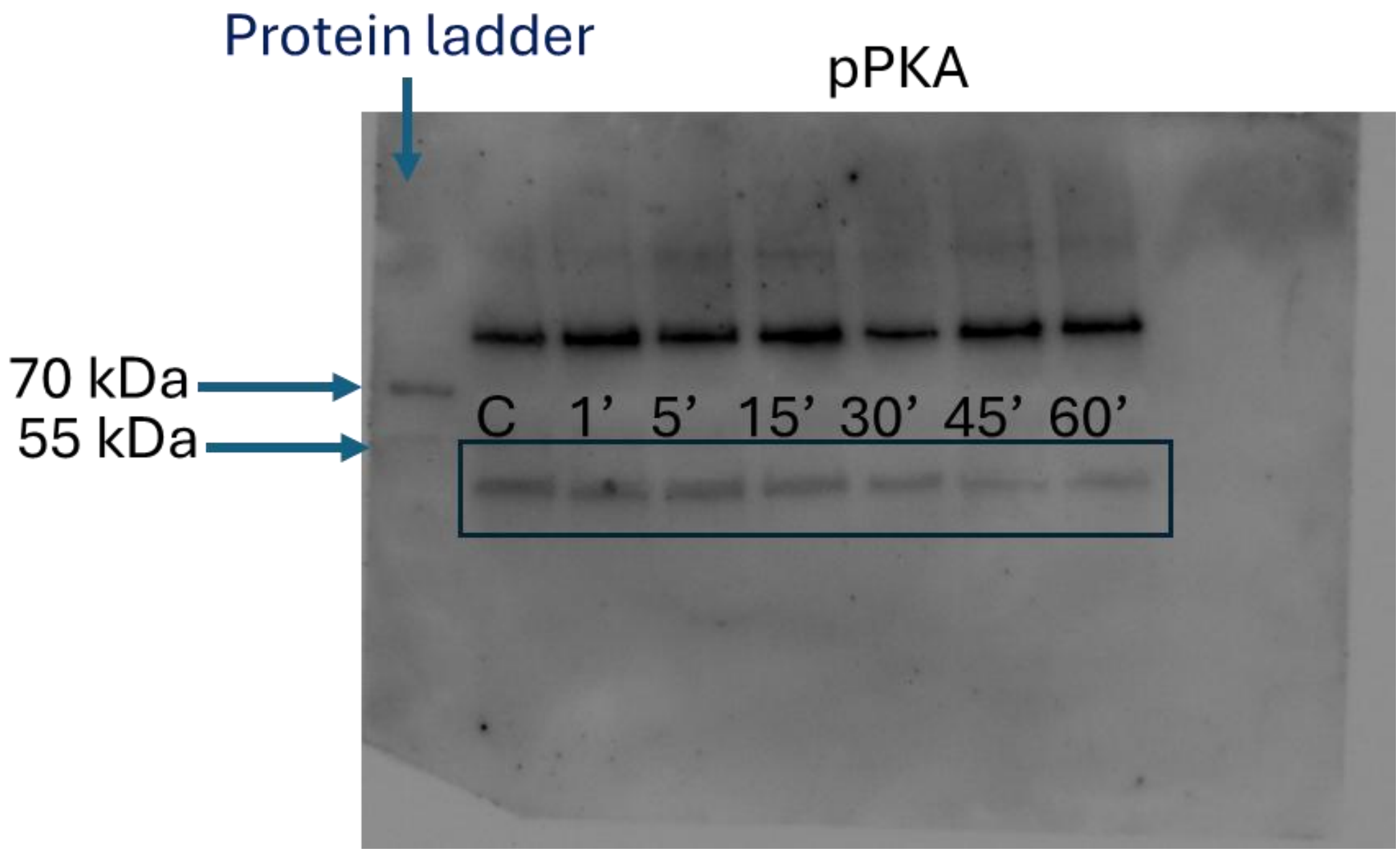

tPKA: 41 kDa

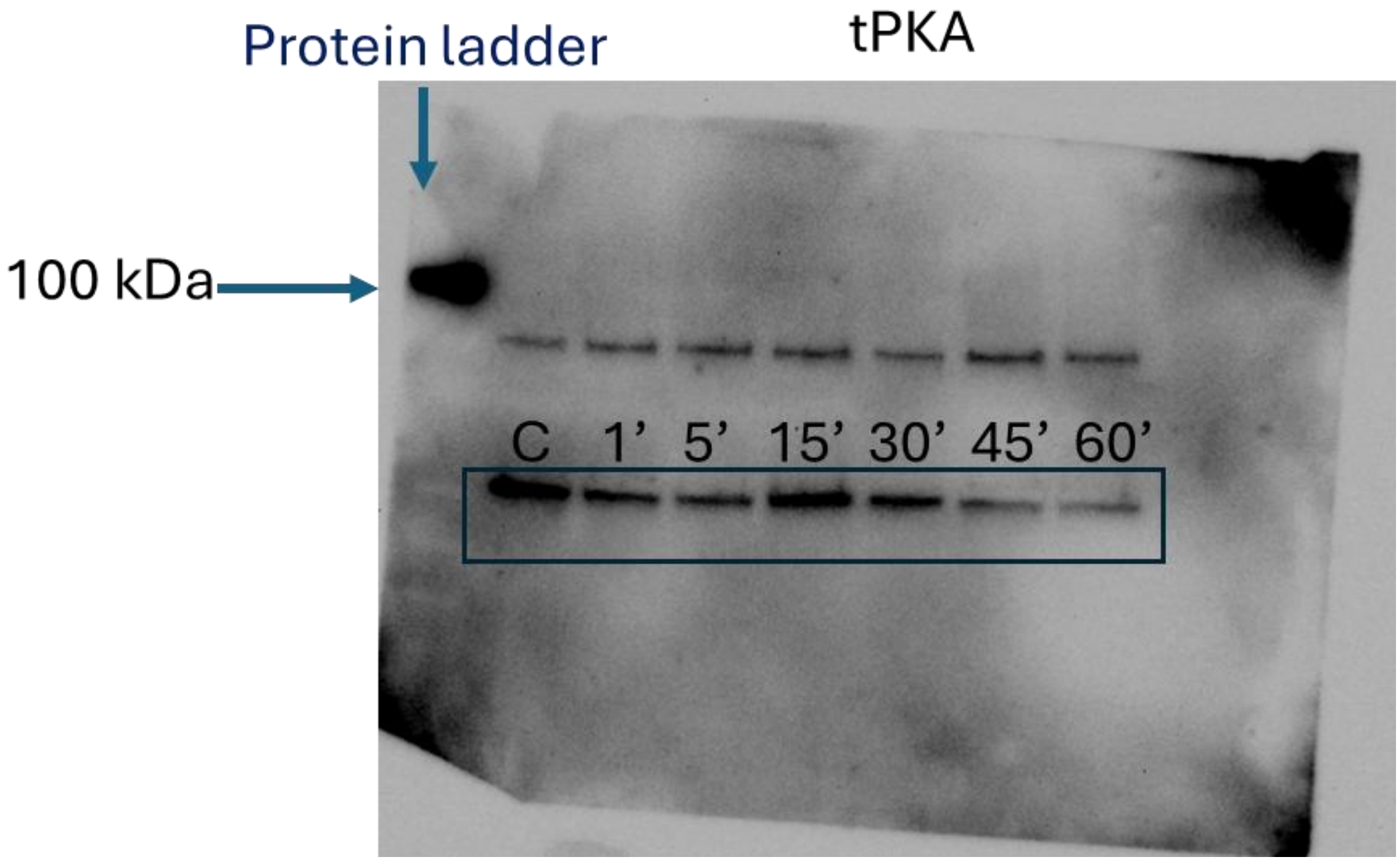

Figures legend:

[empty box]	lanes included in the final figure
C	control
V10	visfatin 10 ng/mL
NP	normal placenta
IUGR	placenta from intrauterine growth restriction
PE	placenta from preeclampsia
GDM	placenta from gestational diabetes mellitus
3β-HSD	3β-hydroxysteroid dehydrogenase
CYP19	aromatase
hCG	human chorionic gonadotropin
hPL	human placental lactogen
GH2	human placental growth hormone
pPKA	phosphorylated isoform of protein kinase A
tPKA	total isoform of protein kinase A
ACTB	β-actin

Images of the cultured villous explants (10× objective)

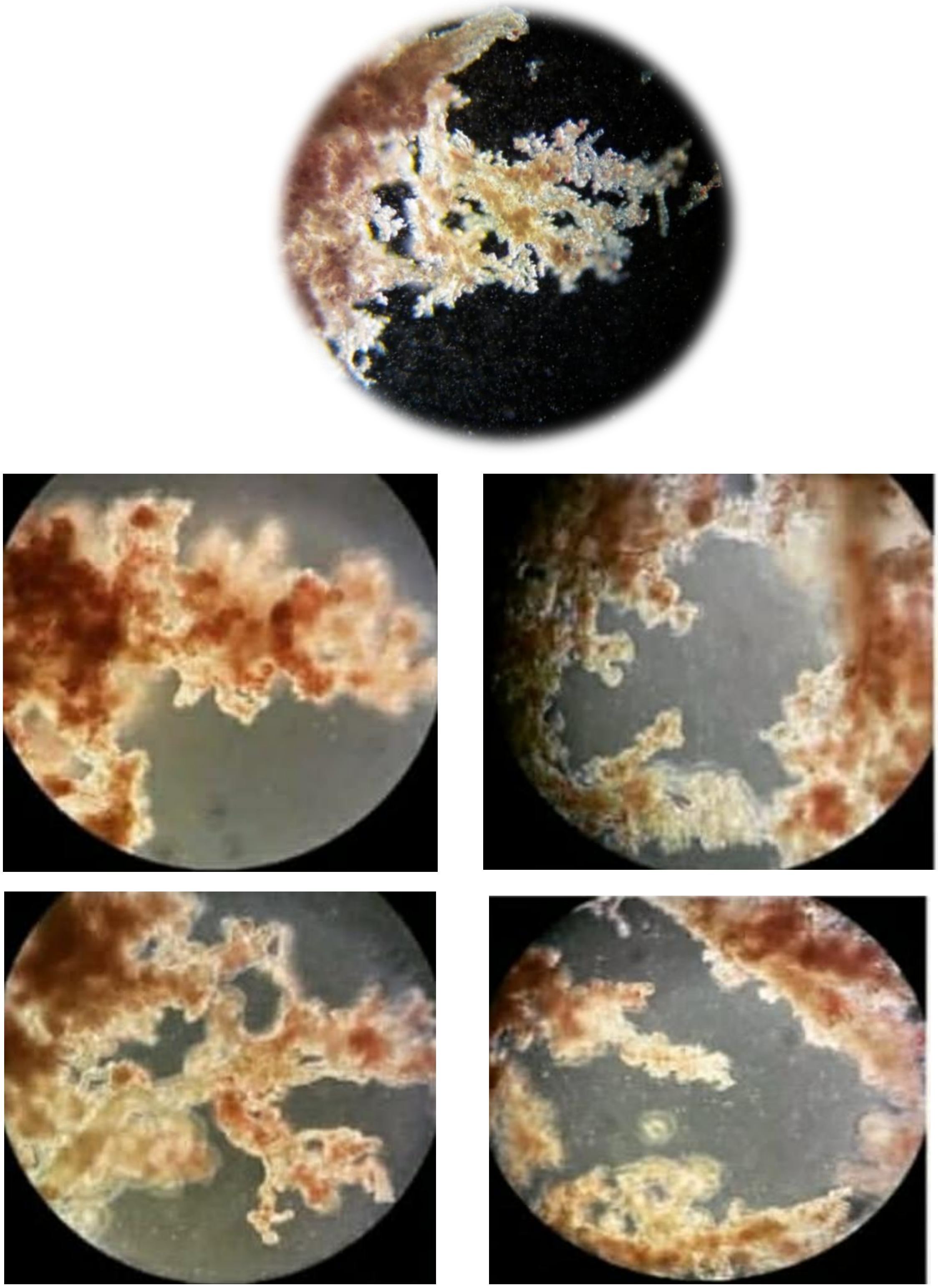

Supplement: Supplementary file 1 [file 41598_2026_52422_MOESM1_ESM.pdf]
